# The total antioxidant content of more than 3100 foods, beverages, spices, herbs and supplements used worldwide

**DOI:** 10.1186/1475-2891-9-3

**Published:** 2010-01-22

**Authors:** Monica H Carlsen, Bente L Halvorsen, Kari Holte, Siv K Bøhn, Steinar Dragland, Laura Sampson, Carol Willey, Haruki Senoo, Yuko Umezono, Chiho Sanada, Ingrid Barikmo, Nega Berhe, Walter C Willett, Katherine M Phillips, David R Jacobs, Rune Blomhoff

**Affiliations:** 1 Department of Nutrition, Institute of Basic Medical Sciences, University of Oslo, Oslo, Norway; 2 The Norwegian Institute for Agricultural and Environmental Research Bioforsk Øst Apelsvoll, Kapp, Norway; 3 Department of Nutrition, Harvard School of Public Health, Boston, Massachusetts, USA; 4 Department of Cell Biology and Morphology, Akita University Graduate School of Medicine, Akita City, Japan; 5 Faculty of Health, Nutrition and Management, Akershus University College, Lillestrøm, Norway; 6 The Biochemistry Department, Virginia Polytechnic Institute and State University, Blacksburg, VA, USA; 7 The Division of Epidemiology and Community Health, School of Public Health, University of Minnesota, Minneapolis, USA

## Abstract

**Background:**

A plant-based diet protects against chronic oxidative stress-related diseases. Dietary plants contain variable chemical families and amounts of antioxidants. It has been hypothesized that plant antioxidants may contribute to the beneficial health effects of dietary plants. Our objective was to develop a comprehensive food database consisting of the total antioxidant content of typical foods as well as other dietary items such as traditional medicine plants, herbs and spices and dietary supplements. This database is intended for use in a wide range of nutritional research, from in vitro and cell and animal studies, to clinical trials and nutritional epidemiological studies.

**Methods:**

We procured samples from countries worldwide and assayed the samples for their total antioxidant content using a modified version of the FRAP assay. Results and sample information (such as country of origin, product and/or brand name) were registered for each individual food sample and constitute the Antioxidant Food Table.

**Results:**

The results demonstrate that there are several thousand-fold differences in antioxidant content of foods. Spices, herbs and supplements include the most antioxidant rich products in our study, some exceptionally high. Berries, fruits, nuts, chocolate, vegetables and products thereof constitute common foods and beverages with high antioxidant values.

**Conclusions:**

This database is to our best knowledge the most comprehensive Antioxidant Food Database published and it shows that plant-based foods introduce significantly more antioxidants into human diet than non-plant foods. Because of the large variations observed between otherwise comparable food samples the study emphasizes the importance of using a comprehensive database combined with a detailed system for food registration in clinical and epidemiological studies. The present antioxidant database is therefore an essential research tool to further elucidate the potential health effects of phytochemical antioxidants in diet.

## Background

It is widely accepted that a plant-based diet with high intake of fruits, vegetables, and other nutrient-rich plant foods may reduce the risk of oxidative stress-related diseases [1-6]. Understanding the complex role of diet in such chronic diseases is challenging since a typical diet provides more than 25,000 bioactive food constituents [6], many of which may modify a multitude of processes that are related to these diseases. Because of the complexity of this relationship, it is likely that a comprehensive understanding of the role of these bioactive food components is needed to assess the role of dietary plants in human health and disease development. We suggest that both their numerous individual functions as well as their combined additive or synergistic effects are crucial to their health beneficial effects, thus a food-based research approach is likely to elucidate more health effects than those derived from each individual nutrient. Most bioactive food constituents are derived from plants; those so derived are collectively called phytochemicals. The large majority of these phytochemicals are redox active molecules and therefore defined as antioxidants. Antioxidants can eliminate free radicals and other reactive oxygen and nitrogen species, and these reactive species contribute to most chronic diseases. It is hypothesized that antioxidants originating from foods may work as antioxidants in their own right in vivo, as well as bring about beneficial health effects through other mechanisms, including acting as inducers of mechanisms related to antioxidant defense [7,8], longevity [9,10], cell maintenance and DNA repair [11].

Several assays have been used to assess the total antioxidant content of foods, e.g. the 6-hydroxy-2,5,7,8-tetramethylchroman-2-carboxylic acid (Trolox) equivalent antioxidant capacity (TEAC) assay [12], the ferric-reducing ability of plasma (FRAP) [13] and the oxygen radical absorbance capacity assay (ORAC) assay [14]. Based on careful considerations (see Blomhoff 2005 and Halvorsen et al 2002 for discussion [15,16]) we chose to use a modified version of the FRAP assay by Benzie and Strain [13] for total antioxidant analysis [16]. Most importantly, the modified FRAP assay is a simple, fast and inexpensive assay with little selectivity. Assay conditions, such as extraction solvents, were optimized regarding detection of both lipophilic and hydrophilic antioxidants [16]. The FRAP assay directly measures antioxidants with a reduction potential below the reduction potential of the Fe^3+^/Fe^2+ ^couple [16,17]. Thus, the FRAP assay does not measure glutathione. Most other assays have higher reduction potentials and measures glutathione and other thiols [18]. This may be an advantage when using the FRAP assay, because glutathione is found in high concentrations in foods but it is degraded in the intestine and poorly absorbed by humans [19]. A disadvantage of the FRAP assay is its inability to detect other small molecular weight thiols and sulfur containing molecules of e.g. garlic. Most assays for assessing total antioxidant capacity generally result in similar ranking of foods [20-23]. We have now performed a systematic measurement of the total antioxidant content of more than 3100 foods. This novel Antioxidant Food Table enables us to calculate total antioxidant content of complex diets, identify and rank potentially good sources of antioxidants, and provide the research community with comparable data on the relative antioxidant capacity of a wide range of foods.

There is not necessarily a direct relationship between the antioxidant content of a food sample consumed and the subsequent antioxidant activity in the target cell. Factors influencing the bioavailability of phytochemical antioxidants, include the food matrix, absorption and metabolism [24-27]. Also, the methods measuring total antioxidant capacity do not identify single antioxidant compounds, and they are therefore of limited use when investigating the mechanisms involved. This is however, not the scope of this article. With the present study, food samples with high antioxidant content are identified, but further investigation into each individual food and phytochemical antioxidant compound is needed to identify those which may have biological relevance and the mechanisms involved.

The aim of the present study was to screen foods to identify total antioxidant capacity of fruits, vegetables, beverages, spices and herbs in addition to common everyday foods. In nutritional epidemiologic and intervention studies, the Antioxidant Food Database may be utilized to identify and rank diets and subjects with regard to antioxidant intake and as a tool in planning dietary antioxidant interventions. The database will be available online at the University of Oslo's web site.

## Methods

### Reagents

TPTZ (2,4,6-tri-pyridyl-s-triazine) was obtained from Fluka Chemie AG (Deisenhofen, Switzerland), sodium acetate trihydrate and FeSO_4 _× 7 H_2_O from Riedel-deHaën AG (Seelze, Germany), acetic acid and hydrochloric acid from Merck (Darmstadt, Germany), FeCl_3 _× 6H_2_O from BDH Laboratory Supplies (Dorset, England). MilliQ water (Millipore, Bedford, MA) and methanol of HPLC-grade obtained from Merck was used for all extractions. 2-propanol (HPLC-grade) was obtained from Merck.

### Sample collection and sample preparation

The antioxidant measurements have been conducted over a period of eight years, from 2000 to 2008. The samples were procured from local stores and markets in Scandinavia, USA and Europe and from the African, Asian and South American continents. Many of the samples of plant material, like berries, mushrooms and herbs, were handpicked. Commercially procured food samples were stored according to the description on the packing and analyzed within four weeks. Handpicked samples were either stored at 4°C and analyzed within three days or frozen at -20°C and analyzed within four weeks. Products that needed preparation such as coffee, tea, processed vegetables etc. were prepared on the day of analysis. Furthermore, all samples were homogenized, dry samples were pulverized and solid samples were chopped in a food processor. After homogenizing, analytical aliquots were weighed. Included in the database are 1113 of the food samples obtained from the US Department of Agriculture National Food and Nutrient Analysis Program. They were collected, homogenized, and stored as previously described [17]. Three replicates were weighed out for each sample. All samples were extracted in water/methanol, except vegetable oils which were extracted in 2-propanol and some fat-rich samples which were extracted in water/2-propanol. The extracts were mixed, sonicated in ice water bath for 15 min, mixed once more and centrifuged in 1.5 mL tubes at 12.402 × g for 2 min at 4°C. The concentration of antioxidants was measured in triplicate of the supernatant of the centrifuged samples.

### Measurements of antioxidant content

The FRAP assay of Benzie and Strain [13] was used with minor modifications that allowed quantification of most water- and fat-soluble antioxidants [16,17]. A Technicon RA 1000 system (Technicon instruments corporation, New York, USA) was used for the measurements of absorption changes that appear when the TPTZ-Fe^3+ ^complex reduces to the TPTZ-Fe^2+ ^form in the presence of antioxidants. An intense blue color with absorption maximum at 593 nm develops. The measurements were performed at 600 nm after 4 min incubation. An aqueous solution of 500 μmol/L FeSO_4 _× 7 H_2_O was used for calibration of the instrument. Validation of the assay is described in Halvorsen et al. 2002 [17]. Briefly, the within-day repeatability measured as relative standard deviation (RSD) in standard solutions ranged from 0.4% to 6%. The between-day repeatability was < 3%. The variation in the values for replicate food items obtained from the same source were typically between 3 and 10 RSD%.

### Organization of the Antioxidant Food Table

The samples were classified into 24 different categories covering products from the plant kingdom, products from the animal kingdom and mixed food products. Information about sample processing (raw, cooked, dried etc), if any, was included, along with all sample specifications, i.e. product name, brand name, where the product/sample was procured and country of origin. The product information in the database was collected from the packing of the product, from supplier or purchaser. When this information was not available or the samples were handpicked, only country of origin is presented. Each sample is assigned to only one category. The classification was done according to information from the supplier or purchaser, or according to common traditional use of the food. Some foods may therefore be categorized otherwise in other food cultures. For products in the categories "Herbal/traditional plant medicine" and "Vitamin and dietary Supplements" some products may rightfully be classified as both an herbal medicine and a supplement, but are still assigned to only one category. All berries, fruits, and vegetables were fresh samples unless otherwise noted in the database. The Antioxidant Food Table contains 3139 samples. About 1300 of these samples have been published before [16,17,28] but for comparison and completeness we have included them in the present publication. All individual samples previously published are identified by a comment in the Antioxidant Food Table. The categories and products in the database are presented in alphabetic order. Information about brand names and product trademarks does not imply endorsement by the authors, and are reported as descriptive information for research applications only. The Antioxidant Food Table will in the future be available online as a searchable database. In addition to the products mentioned in this paper, other foods will in the future be analyzed and incorporated into the online version, which will be posted on the University of Oslo's web site.

## Results

Our results show large variations both between as well as within each food category; all of the food categories contain products almost devoid of antioxidants (Table 1). Please refer to Additional file 1, the Antioxidant Food Table, for the FRAP results on all 3139 products analyzed. The categories "Spices and herbs", "Herbal/traditional plant medicine" and "Vitamin and dietary supplements" include the most antioxidant rich products analyzed in the study. The categories "Berries and berry products", "Fruit and fruit juices", "Nuts and seeds", "Breakfast Cereals", "Chocolate and sweets", "Beverages" and "Vegetables and vegetable products" include most of the common foods and beverages which have medium to high antioxidant values (Table 1). We find that plant-based foods are generally higher in antioxidant content than animal-based and mixed food products, with median antioxidant values of 0.88, 0.10 and 0.31 mmol/100 g, respectively (Table 1). Furthermore, the 75^th ^percentile of plant-based foods is 4.11 mmol/100 g compared to 0.21 and 0.68 mmol/100 g for animal-based and mixed foods, respectively. The high mean value of plant-based foods is due to a minority of products with very high antioxidant values, found among the plant medicines, spices and herbs. In the following, summarized results from the 24 categories are presented.

**Table 1:** Statistical descriptives of the Antioxidant Food Table and individual categories.

	Antioxidant content in mmol/100 g							
	
	n	mean	median	min	max	25th percentile	75th percentile	90th percentile
Plant based foods ^a)^	1943	11.57	0.88	0.00	2897.11	0.27	4.11	24.30
Animal based foods ^b)^	211	0.18	0.10	0.00	1.00	0.05	0.21	0.46
Mixed foods ^c)^	854	0.91	0.31	0.00	18.52	0.14	0.68	1.50
								
Categories								
1 Berries and berry products	119	9.86	3.34	0.06	261.53	1.90	6.31	37.08
2 Beverages	283	8.30	0.60	0.00	1347.83	0.15	2.37	3.64
3 Breakfast cereals	90	1.09	0.89	0.16	4.84	0.53	1.24	1.95
4 Chocolates and sweets	80	4.93	2.33	0.05	14.98	0.82	8.98	13.23
5 Dairy products	86	0.14	0.06	0.00	0.78	0.04	0.14	0.44
6 Desserts and cakes	134	0.45	0.20	0.00	4.10	0.09	0.52	1.04
7 Egg	12	0.04	0.04	0.00	0.16	0.01	0.06	0.14
8 Fats and oils	38	0.51	0.39	0.19	1.66	0.30	0.50	1.40
9 Fish and seafood	32	0.11	0.08	0.03	0.65	0.07	0.12	0.21
10 Fruit and fruit juices	278	1.25	0.69	0.03	55.52	0.31	1.21	2.36
11 Grains and grain products	227	0.34	0.18	0.00	3.31	0.06	0.38	0.73
12 Herbal/traditional plant medicine	59	91.72	14.18	0.28	2897.11	5.66	39.67	120.18
13 Infant foods and beverages	52	0.77	0.12	0.02	18.52	0.06	0.43	1.17
14 Legumes	69	0.48	0.27	0.00	1.97	0.12	0.78	1.18
15 Meat and meat products	31	0.31	0.32	0.00	0.85	0.11	0.46	0.57
16 Miscellaneous ingredients, condiments	44	0.77	0.15	0.00	15.54	0.03	0.41	1.70
17 Mixed food entrees	189	0.19	0.16	0.03	0.73	0.11	0.23	0.38
18 Nuts and seeds	90	4.57	0.76	0.03	33.29	0.44	5.08	15.83
19 Poultry and poultry products	50	0.23	0.15	0.05	1.00	0.12	0.23	0.59
20 Snacks, biscuits	66	0.58	0.61	0.00	1.17	0.36	0.77	0.97
21 Soups, sauces gravies, dressing	251	0.63	0.41	0.00	4.67	0.25	0.68	1.27
22 Spices and herbs	425	29.02	11.30	0.08	465.32	4.16	35.25	74.97
23 Vegetables and vegetable products	303	0.80	0.31	0.00	48.07	0.17	0.68	1.50
24 Vitamin and dietary supplements	131	98.58	3.27	0.00	1052.44	0.62	62.16	316.93

^a) ^Categories 1, 2, 3, 10, 11, 12, 14, 18, 22, 23

^b) ^Categories 5, 7, 9, 15, 19

^c) ^Categories 4, 6, 8, 13, 16, 17, 20, 21

### Beverages

In the category "Beverages", 283 products were included, from coffee and tea to beer, wine and lemonades. Dry products like coffee beans and dried tea leaves and powders were also included. The highest antioxidant values in this category were found among the unprocessed tea leaves, tea powders and coffee beans. In Table 2 we present an excerpt of this category and of the analyses of fruit juices. Fifty-four different types of prepared coffee variants procured from 16 different manufacturers showed that the variation in coffees are large, ranging from a minimum of 0.89 mmol/100 g for one type of brewed coffee with milk to 16.33 mmol/100 g for one type of double espresso coffee, the highest antioxidant value of all prepared beverages analyzed in the present study. Other antioxidant rich beverages are red wine, which have a smaller variation of antioxidant content (1.78 to 3.66 mmol/100 g), pomegranate juice, prepared green tea (0.57 to 2.62 mmol/100 g), grape juice, prune juice and black tea (0.75 to 1.21 mmol/100 g) (Table 2). Beer, soft drinks and ginger ale contain the least antioxidants of the beverages in our study, with drinking water completely devoid of antioxidants.

**Table 2:** Excerpt of the analyses of beverages in the Antioxidant Food Table.

	Antioxidant content mmol/100 g^a)^	n	min	max
Apple juice	0.27	11	0.12	0.60
Black tea, prepared	1.0	5	0.75	1.21
Cocoa with milk	0.37	4	0.26	0.45
Coffee, prepared filter and boiled	2.5	31	1.24	4.20
Cranberry juice	0.92	5	0.75	1.01
Espresso, prepared	14.2	2	12.64	15.83
Grape juice	1.2	6	0.69	1.74
Green tea, prepared	1.5	17	0.57	2.62
Orange juice	0.64	16	0.47	0.81
Pomegranate juice	2.1	2	1.59	2.57
Prune juice	1.0	3	0.83	1.13
Red wine	2.5	27	1.78	3.66
Tomato juice	0.48	14	0.19	1.06

^a) ^Mean value when n > 1

### Breakfast cereals, grains, legumes, nuts and seeds

Most of the breakfast cereals have antioxidant content in the range of 0.5 to 2.25 mmol/100 g, while 4 single products are above this range. Among grains and grain products, buckwheat, millet and barley flours are the flours with the highest antioxidant values in our study (Table 3), while crisp bread and whole meal bread with fiber are the grain products containing most antioxidants. Beans and lentils have mean antioxidant values ranging from 0.1 to 1.97 mmol/100 g. Different types of rice have antioxidant values between 0.01 and 0.36 mmol/100 g.

**Table 3:** Excerpt of the analyses of nuts, legumes and grain products in the Antioxidant Food Table.

	Antioxidant content mmol/100 g^a)^	n	Min	Max
Barley, pearl and flour	1.0	4	0.74	1.19
Beans	0.8	25	0.11	1.97
Bread, with fiber/whole meal	0.5	3	0.41	0.63
Buckwheat, white flour	1.4	2	1.08	1.73
Buckwheat, whole meal flour	2.0	2	1.83	2.24
Chestnuts, with pellicle	4.7	1	-	-
Crisp bread, brown	1.1	3	0.93	1.13
Maize, white flour	0.6	3	0.32	0.88
Millet	1.3	1	-	-
Peanuts, roasted, with pellicle	2.0	1	-	-
Pecans, with pellicle	8.5	7	6.32	10.62
Pistachios	1.7	7	0.78	4.98
Sunflower seeds	6.4	2	5.39	7.50
Walnuts, with pellicle	21.9	13	13.13	33.29
Wheat bread, toasted	0.6	3	0.52	0.59
Whole wheat bread, toasted	1.0	2	0.93	1.00

mean value when n > 1

In the nuts and seeds category we analyzed 90 different products, with antioxidant contents varying from 0.03 mmol/100 g in poppy seeds to 33.3 mmol/100 g in walnuts, with pellicle and purchased with nut shell intact. Pecans with pellicle, sunflower seeds and chestnuts with pellicle, have mean antioxidant content in the range of 4.7 to 8.5 mmol/100 g (Table 3). Walnuts, chestnuts, peanuts, hazelnuts and almonds have higher values when analyzed with the pellicle intact compared to without pellicle.

### Chocolate

Various types of chocolate were analyzed, from milk chocolate to dark chocolate and baking cocoa. The variation of antioxidant content in chocolate ranged from 0.23 in white chocolate to 14.98 mmol/100 g in one individual dark chocolate sample. Mean antioxidant contents increased with increasing content of cocoa in the chocolate product (Pearson correlation r = 0.927, p < 0.001). Chocolate products with cocoa contents of 24-30%, 40-65% and 70-99% had mean antioxidant contents of 1.8, 7.2 and 10.9 mmol/100 g, respectively.

### Dairy products, desserts and cakes, eggs, fats and oils

The dairy category included 86 products and the majority of these products were low in antioxidant content, in the range of 0.0 to 0.8 mmol/100 g. Dairy products with added berries or chocolate and cheeses like Brie, Gorgonzola and Roquefort are the most antioxidant rich products in this category.

One hundred and thirty four products are included in the category "Desserts and cakes". In the upper range of this category we find dog rose soup and chocolate cookies. Eggs are almost devoid of antioxidants with the highest antioxidant values found in egg yolk (0.16 mmol/100 g).

Margarine, butter, canola, corn and soybean oil are the highest ranking products in the "Fats and oils" category. Almost half of the fats and oils have antioxidant content between 0.4 and 1.7 mmol/100 g.

### Berries, fruit and vegetables

In Table 4 we present an excerpt of the all the berries, fruits and vegetables analyzed. One hundred and nineteen berries and berry products were analyzed. The average antioxidant content of berries and berry products is relatively high with 25^th ^and 75^th ^percentiles being 1.90 to 6.31 mmol/100 g, respectively. There were 13 samples with especially high antioxidant capacity in this category, including dried amla (Indian gooseberry, 261.5 mmol/100 g), wild dried dog rose (*Rosa canina*) and products of dried dog rose with antioxidant contents in the range from 20.8 to 78.1 mmol/100 g. Dried wild bilberries (*Vaccinum Myrtillus*, native to Northern Europe), zereshk (red sour berries) from Iran and fresh dog rose (from Norway and Spain) have mean antioxidant contents of 48.3, 27.3 and 24.3 mmol/100 g, respectively. Other examples of antioxidant rich berries are fresh crowberries, bilberries, black currants, wild strawberries, blackberries, goji berries, sea buckthorn and cranberries. The least antioxidant rich berry products are some of the berry jams with mean values of approximately 0.5 mmol/100 g.

**Table 4:** Excerpt of the berries, fruit and vegetable analyses in the Antioxidant Food Table.

	Antioxidant content mmol/100 g^a)^	n	Min	Max
African baobab tree, leaves dry, crushed	48.1	1	-	-
Amla (Indian gooseberry), dried	261.5	1	-	-
Apples	0.4	15	0.1	1.22
Apples, dried	3.8	3	1.86	6.07
Apricots, dried	3.1	4	1.32	4.67
Artichoke	3.5	8	0.69	4.76
Bilberries, dried	48.3	1	-	-
Black olives	1.7	6	0.23	3.25
Blueberry jam	3.5	4	2.68	4.71
Broccoli, cooked	0.5	4	0.25	0.85
Chilli, red and green	2.4	3	2.08	2.92
Curly kale	2.8	4	1.62	4.09
Dates, dried	1.7	2	1.53	1.88
Dog rose, products of dried hip	69.4	3	54.30	75.84
Dog rose, wild, dried	78.1	1	-	-
Dog rose, wild, fresh	24.3	3	12.65	34.49
Fruit from the African baobab tree	10.8	1	-	-
Mango, dried	1.7	2	0.58	2.82
*Moringa Stenopetala*, dried leaves, stem	11.9	1	-	-
*Moringa Stenopetala*, fresh leaves, stem	3.7	1	-	-
Okra/gumbo from Mali, dry, flour	4.2	1	-	-
Oranges	0.9	3	0.83	1.08
Papaya	0.6	2	0.36	0.76
Plums, dried	3.2	1		
Pomegranate	1.8	6	0.88	2.26
Prunes	2.4	6	1.95	3.70
Strawberries	2.1	4	1.85	2.33
Zereshk, red sour berries	27.3	1	-	-

mean value when n > 1

A total of 278 fruits and fruit products and 303 vegetables and vegetable products were included in the database. In the analyzed vegetables, antioxidant content varied from 0.0 mmol/100 g in blanched celery to 48.1 mmol/100 g in dried and crushed leaves of the African baobab tree. In fruits, procured in 8 different countries, the antioxidant content varies from 0.02 mmol/100 g for watermelon to 55.5 mmol/100 g in the yellow pith of Spanish pomegranate. Examples of antioxidant rich fruits and vegetables were dried apples, flour made of okra, artichokes, lemon skin, dried plums, dried apricots, curly kale, red and green chili and prunes (Table 4). Examples of fruit and vegetables in the medium antioxidant range were dried dates, dried mango, black and green olives, red cabbage, red beets, paprika, guava and plums.

### Herbal/traditional plant medicine

This is the most antioxidant rich category in the present study and is also the category with largest variation between products. Half of the products have antioxidant values above the 90^th ^percentile of the complete Antioxidant Food Table and the mean and median values are 91.7 and 14.2 mmol/100 g, respectively. The 59 products included originate from India, Japan, Mexico and Peru. Sangre de Grado (Dragon's Blood) from Peru has the highest antioxidant content of all the products in the database (2897.1 mmol/100 g). Other antioxidant rich products are Triphala, Amalaki and Arjuna from India and Goshuyu-tou, a traditional kampo medicine from Japan, with antioxidant values in the range of 132.6 to 706.3 mmol/100 g. Only four products in this category have values less than 2.0 mmol/100 g.

### Infant food and beverages

The category includes 52 products, including European, Scandinavian and American products. The variation in antioxidant content in dinner and dessert products for infants varies from 0.02 to 1.25 mmol/100 g. Interestingly, human breast milk (49 samples from Norwegian mothers) has a mean content of 2.0 mmol/100 g. In addition, the category includes two Norwegian dog rose products for infants with antioxidant contents of 6.7 and 18.5 mmol/100 g.

### Spices and herbs

An excerpt of the 425 spices and herbs analyzed in our study are presented in Table 5. The study includes spices and herbs from 59 different manufacturers or countries. Twenty seven single products are in the range 100 to 465 mmol/100 g, but the variation is from 0.08 mmol/100 g in raw garlic paste procured in Japan, to 465 mmol/100 g in dried and ground clove purchased in Norway. Sorted by antioxidant content, clove has the highest mean antioxidant value, followed by peppermint, allspice, cinnamon, oregano, thyme, sage, rosemary, saffron and estragon, all dried and ground, with mean values ranging from 44 to 277 mmol/100 g. When analyzed in fresh samples compared to dried, oregano, rosemary and thyme have lower values, in the range of 2.2 to 5.6 mmol/100 g. This is also true for basil, chives, dill and parsley. In addition to common spices and culinary herbs, we have also analyzed other herbs, like birch leaves, wild marjoram and wood cranesbill among others. Details on all herbs can be found in Additional file 1, the Antioxidant Food Table.

**Table 5:** Excerpt of the spices and herbs analyzed in the Antioxidant Food Table.

	Antioxidant content mmol/100 g^a)^	n	Min	Max
Allspice, dried ground	100.4	2	99.28	100.40
Basil, dried	19.9	5	9.86	30.86
Bay leaves, dried	27.8	2	24.29	31.29
Cinnamon sticks and whole bark	26.5	3	6.84	40.14
Cinnamon, dried ground	77.0	7	17.65	139.89
Clove, dried, whole and ground	277.3	6	175.31	465.32
Dill, dried ground	20.2	3	15.94	24.47
Estragon, dried ground	43.8	3	43.22	44.75
Ginger, dried	20.3	5	11.31	24.37
Mint leaves, dried	116.4	2	71.95	160.82
Nutmeg, dried ground	26.4	5	15.83	43.52
Oregano, dried ground	63.2	9	40.30	96.64
Rosemary, dried ground	44.8	5	24.34	66.92
Saffron, dried ground	44.5	3	23.83	61.72
Saffron, dried whole stigma	17.5	3	7.02	24.83
Sage, dried ground	44.3	3	34.88	58.80
Thyme, dried ground	56.3	3	42.00	63.75

^a) ^mean value when n > 1

### Soups, sauces, gravies and dressings

In this broad category, we have analyzed 251 products and found that the products with highest antioxidant content are tomato based sauces, basil pesto, mustard paste, sun dried tomatoes and tomato paste/puree, in the range of 1.0 to 4.6 mmol/100 g.

### Vitamin and dietary supplements

The category "Vitamin and dietary supplements" includes 131 commercially available vitamin and dietary supplement products from USA, Norway, Mexico and Japan of which many have high antioxidant scores. Among them are supplements containing anthocyanins, vitamin C, green tea powder and multivitamins and multi-antioxidant tablets.

### Meat, poultry, fish and miscellaneous ingredients

The majority of the products in these categories were low in antioxidant content. Nevertheless, products like liver, bacon and some prepared chicken and beef products have antioxidant values between 0.5 and 1.0 mmol/100 g.

## Discussion

With this study we present a comprehensive survey of the total antioxidant capacity in foods. Earlier small-scale studies from other laboratories have included from a few up to a few hundred samples [20-22,29-31], and in 2007 the U.S. Department of Agriculture presented the Oxygen Radical Absorbance Capacity (ORAC) of Selected Foods report including 277 food samples [23]. These studies have been done using different antioxidant assays for measuring antioxidant capacity making it difficult to compare whole lists of foods, products and product categories. Still, a food that has a high total antioxidant capacity using one antioxidant assay will most likely also be high using another assay [20-22]. Consequently, the exact value will be different but the ranking of the products will be mainly the same whichever assay is used. In the present extensive study, the same validated method has been used on all samples, resulting in comparable measures, thus enabling us to present a complete picture of the relative antioxidant potential of the samples.

When classifying the samples into the three main classes the difference in antioxidant content between plant- and animal-based foods become apparent. The results here uncover that the antioxidant content of foods varies several thousand-fold and that antioxidant rich foods originate from the plant kingdom while meat, fish and other foods from the animal kingdom are low in antioxidants. Comparing the mean value of the 'Meat and meat products' category with plant based categories, fruits, nuts, chocolate and berries have from 5 to 33 times higher mean antioxidant content than the mean of meat products. Diets comprised mainly of animal-based foods are thus low in antioxidant content while diets based mainly on a variety of plant-based foods are antioxidant rich, due to the thousands of bioactive antioxidant phytochemicals found in plants which are conserved in many foods and beverages.

Most of the spices and herbs analyzed have particularly high antioxidant contents. Although spices and herbs contribute little weight on the dinner plate, they may still be important contributors to our antioxidant intake, especially in dietary cultures where spices and herbs are used regularly. We interpret the elevated concentration of antioxidants observed in several dried herbs compared to fresh samples, as a normal consequence of the drying process leaving most of the antioxidants intact in the dried end product. This tendency is also seen in some fruits and their dried counterparts. Thus, dried herbs and fruit are potentially excellent sources of antioxidants.

Herbal and traditional plant medicines emerged as many of the highest antioxidant-containing products in our study. We speculate that the high inherent antioxidant property of many plants is an important contributor to the herb's medicinal qualities. In our study we identified Sangre de Grado, the sap from the tree trunk of the species *Croton lechleri *sampled in Peru to have exceptional high antioxidant content. This sap has a long history of indigenous use in South America for wound healing and as an antifungal, antiseptic, antiviral and antihaemorrhagic medicine. Proanthocyanidins are major constituents of this sap [32] and studies have shown that Sangre de Grado limits the transcription of a wide range of pro-inflammatory cytokines and mediators and accelerates the healing of stomach ulcers [33,34] and promotes apoptosis in cancer cells [35]. Other extreme antioxidant rich herbal medicines are Triphala, an Indian Ayurvedic herbal formulation, shown to have anti-inflammatory activity [36], antibacterial and wound healing properties [37,38] and cancer chemopreventive potential [39]. Arjuna, another Auyrvedic formula, has been shown to have health beneficial activities [40,41] while Goshuyu-tou, a traditional Chinese kampo medicine has been shown to significantly reduce the extracellular concentration of NO in the LPS-stimulated Raw 264.7 cells [42].

With their high content of phytochemicals such as flavonoids, tannins, stilbenoids, phenolic acids and lignans [43-45] berries and berry products are potentially excellent antioxidant sources. The phytochemical content of berries varies with geographical growing condition, and between cultivars [46,47] explaining the variations found in our study. During the processing of berries to jams, total phenol content is reduced [48] resulting in lower antioxidant values in processed berry products than in fresh berries.

Nuts are a rich source of many important nutrients and some are also antioxidant-rich. The observed increase in antioxidant content in nuts with pellicle compared to nuts without pellicle is in good agreement with earlier studies showing the flavonoids of many nuts are found in the nut pellicle [49].

After water, tea and coffee are the two most consumed beverages in the world, although consumption patterns vary between countries. Because of the fairly high content of antioxidants and the frequent use, coffee and tea are important antioxidant sources in many diets. Several different compounds contribute to coffee's antioxidant content, e.g., caffeine, polyphenols, volatile aroma compounds and heterocyclic compounds, [25,50-52]. Many of these are efficiently absorbed, and plasma antioxidants increase after coffee intake [50,53]. In green tea, the major flavonoids present are the monomer catechins, epigallocatechin gallate, epigallocatechin, epicatechin gallate and epicatechin. In black tea the polymerized catechins theaflavin and thearubigen predominate in addition to quercetin and flavonols [54,55].

Interestingly, the antioxidant content in human breast milk is comparable to that in pomegranate juice, strawberries and coffee and on average higher than the antioxidant content observed in the commercially available infant formulas analyzed in our study. Breakfast cereals are also potential important sources of antioxidants; some of these products have antioxidant contents comparable to berries, which are fairly high, compared to other grain products and may be due to antioxidants added to the products in fortification process.

Chocolate have for several years been studied for its possible beneficial health effects [56]. Our results show a high correlation between the cocoa content and the antioxidant content, which is in agreement with earlier studies [30,57].

As demonstrated in the present study, the variation in the antioxidant values of otherwise comparable products is large. Like the content of any food component, antioxidant values will differ for a wide array of reasons, such as growing conditions, seasonal changes and genetically different cultivars [46,58], storage conditions [59-61] and differences in manufacturing procedures and processing [62-64]. Differences in unprocessed and processed plant food samples are also seen in our study where processed berry products like jam and syrup have approximately half the antioxidant capacity of fresh berries. On the other hand, processing may also enhance a foods potential as a good antioxidant source by increasing the amount of antioxidants released from the food matrix which otherwise would be less or not at all available for absorption [65]. Processing of tomato is one such example where lycopene from heat-processed tomato sauce is more bioavailable than unprocessed tomato [66]. The large variations in antioxidant capacity observed in the present study emphasize the importance of using a comprehensive antioxidant database combined with a detailed system for food registration in clinical and epidemiological studies.

Initial studies have been carried out to examine the association between intake of antioxidant rich foods and their health effects [67,70]. Some of these studies describe a beneficial effect on oxidative stress related chronic diseases, e.g. from intake of nuts [49,69], pomegranates [71-73], tomatoes [6], coffee [74], tea [54,75,76], red wine [77-79] and cocoa [56]. The highly reactive and bioactive phytochemical antioxidants are postulated to in part explain the protective effect of plant foods. An optimal mixture of different antioxidants with complementary mechanisms of action and different redox potentials is postulated to work in synergistic interactions. Still, it is not likely that all antioxidant-rich foods are good sources and that all antioxidants provided in the diet are bioactive. Bioavailability differs greatly from one phytochemical to another [26,27,80], so the most antioxidant rich foods in our diet are not necessarily those leading to the highest concentrations of active metabolites in target tissues. The antioxidants obtained from foods include many different molecular compounds and families with different chemical and biological properties that may affect absorption, transport and excretion, cellular uptake and metabolism, and eventually their effects on oxidative stress in various cellular compartments [24]. Biochemically active phytochemicals found in plant-based foods also have many powerful biological properties which are not necessarily correlated with their antioxidant capacity, including acting as inducers of antioxidant defense mechanisms in vivo or as gene expression modulators. Thus a food low in antioxidant content may have beneficial health effects due to other food components or phytochemicals executing bioactivity through other mechanisms.

## Conclusions

The Antioxidant Food Table is a valuable research contribution, expanding the research evidence base for plant-based nutritional research and may be utilized in epidemiological studies where reported food intakes can be assigned antioxidant values. It can also be used to test antioxidant effects and synergy in experimental animal and cell studies or in human clinical trials. The ultimate goal of this research is to combine these strategies in order to understand the role of dietary phytochemical antioxidants in the prevention of cancer, cardiovascular diseases, diabetes and other chronic diseases related to oxidative stress.

## Competing interests

R. Blomhoff is a shareholder in Vitas AS, D.R. Jacobs Jr is an unpaid member of the Scientific Advisory Council of the California Walnut Commission. The other authors declare that they have no competing interests.

## Authors' contributions

MHC took part in planning the study design, contributed to database management, sample procurement, drafting and writing of manuscript. BLH took part in planning the study design and was responsible for assay development and validation, sample analysis, and writing of manuscript, SKB took part in planning the study design and was the database creator and contributed to database management and writing of manuscript, SD, LS, CW, HS, IB, NB, WCW, KMP and DRJ contributed to sample procurement and writing of manuscript, KH, YU and CS contributed to sample procurement and analysis and writing of manuscript, RB was responsible for funding and study design and contributed to sample procurement and writing of manuscript. All authors read and approved the final manuscript.

## Supplementary Material

Additional file 1 **The Antioxidant Food Table, Carlsen et al. 2010**. the main results of the present study; the table includes all the 3139 products with product descriptions, details and antioxidant analysis results, categorized into 24 categories and arranged alphabetically within each category. Click here for file
