# Supplementary material for: The total antioxidant content of more than 3100 foods, beverages, spices, herbs and supplements used worldwide
Source: Nutr J. 2010 Jan 22;9:3. doi: 10.1186/1475-2891-9-3 (PMC2841576; doi:10.1186/1475-2891-9-3)
Supplement: Additional file 1 — The Antioxidant Food Table, Carlsen et al. 2010. the main results of the present study; the table includes all the 3139 products with product descriptions, details and antioxidant analysis results, categorized into 24 categories and arranged alphabetically within each category. [file 1475-2891-9-3-S1.PDF]

Additional file 1: The Antioxidant Food Table.

The total antioxidant content of more than 3,100 foods, beverages, spices, herbs and supplements used worldwide.

MH Carlsen, BL Halvorsen, K Holte, SK Bøhn, S Dragland, L Sampson, C Willey, H Senoo, Y Umezono, C Sanada, I Barikmo, N Behre, WC Willett, KM Phillips, DR Jacobs JR and R Blomhoff.

The 24 categories and all individual products are presented in alphabetic order.

Contents

Category 1 Berries and berry products 3

Category 2 Beverages 9

Category 3 Breakfast cereals 21

Category 4 Chocolate and sweets 24

Category 5 Dairy and dairy products 28

Category 6 Desserts and cakes 32

Category 7 Egg 38

Category 8 Fats and oils 39

Category 9 Fish and seafood 41

Category 10 Fruit and fruit juices 43

Category 11 Grains and grain products 53

Category 12 Herbal / traditional plant medicine 63

Category 13 Infant foods and beverages 67

Category 14 Legumes 69

Category 15 Meat and meat products 72

Category 16 Miscellanous ingredients 73

Category 17 Mixed food entrees 75

Category 18 Nuts and seeds 83

Category 19 Poultry and poultry products 87

Category 20 Snacks 89

Category 21 Soups, sauces, dressings and salsa 92

Category 22 Spices and herbs 102

Category 23 Vegetables.. 122

Category 24 Vitamin and dietary supplements... 133

Category 1 Berries and berry products

Product	Manufacturer / product label / country of origin	Procured in	Antioxidant content in mmol/100g	Comment
Amla berries, dried	India	India	261.53	3
Amla, Indian Gooseberries, whole, canned	Roopaks, Ajmal Khan, N. Dehli	India	13.27	3
Amla, syrup from canned Indian Gooseberries	Roopaks, Ajmal Khan, N. Dehli	India	29.70	3
Bilberries, wild	Norway	Norway	7.57	3
Bilberries, wild	Norway	Norway	8.55	4
Bilberries, wild, dried	The Norwegian Crop Research Institute, Norway	Norway	48.32	5
Blackberries, cultivated		USA	4.02	6
Blackberries, cultivated	Solabær, Sola, Norway	Norway	4.13	3
Blackberries, cultivated		Norway	6.14	3
Blackberries, cultivated	Belgium	Norway	3.84	3
Blackberries, cultivated, canned, drained	S&W Fine Food, USA	Norway	2.34	3
Blackberries, cultivated, frozen	Local grocery	USA	4.06	6
Blackberries, cultivated, frozen	Wholesaler	USA	3.89	6
Blackberries, cultivated, with sugar	Findus, Norway	Norway	4.76	3
Blackberries, Dessert Berries, without sugar, frozen	Norske Dessertbær, Norway (berries from Poland)	Norway	5.98	3
Blackberries, dried, "Loch Ness"	The Norwegian Crop Research Institute, Norway	Norway	37.08	5
Blackberries, wild	Norway	Norway	6.13	4
Blackcurrant, cultivated	Norway	Norway	5.49	3
Blackcurrant, cultivated, "Ben Tiran"	Norwegian University of Life Sciences	Norway	9.09	5
Blackcurrant, syrup (100%), with sugar (undiluted)	Lerum, Norway	Norway	2.38	3

Category 1 Berries and berry products continued

Product	Manufacturer / product label / country of origin	Procured in	Antioxidant content in mmol/100g	Comment
Blackcurrant, syrup (40%), with sugar (undiluted)	Stabburet, Norway	Norway	2.73	3
Blackcurrant, syrup (54%), with sugar (undiluted)	Røra fabrikker, Norway	Norway	2.98	3
Blackcurrant, syrup, without sugar (undiluted)	Helios, Norway	Norway	4.15	3
Blueberries	Poland	Norway	9.24	4
Blueberries, canned, heavy syrup, drained liquid	S&W	USA	2.53	6
Blueberries, canned, heavy syrup, drained solids	S&W	USA	2.79	6
Blueberries, canned, light syrup, drained liquid	Oregon	USA	1.65	6
Blueberries, canned, light syrup, drained solids	Oregon	USA	2.01	6
Blueberries, cultivated	Norway	Norway	1.92	4
Blueberries, cultivated		USA	1.85	6
Blueberries, cultivated		Norway	1.26	3
Blueberries, cultivated, "Aron"	Norwegian University of Life Sciences	Norway	3.79	5
Blueberries, cultivated, "Hardyblue"	Norwegian University of Life Sciences	Norway	3.96	5
Blueberries, Dessert Berries, without sugar, frozen	Norske Dessertbær, Norway (berries from Poland)	Norway	7.13	3
Blueberries, dried	USA, Eurocompany srl, Godo, Italy	Norway	1.32	3
Blueberry, jam	Heistad, Norway	Norway	2.68	3
Blueberry, jam	Eldorado, Norgesgruppen, Norway	Norway	2.88	3
Blueberry, jam, Naturlig lett	Nora, Stabburet, Norway	Norway	4.71	3
Blueberry, jam, Noras hjemmelagde	Nora, Stabburet, Norway	Norway	3.60	3
Blueberry, jam, Wild Blueberry Spread	Meridian Foods, UK	Norway	1.44	3
Blueberry, syrup (undiluted)	Hervik, Norway	Norway	2.41	3
Blueberry, syrup, without sugar (undiluted)	Helios, Norway	Norway	5.91	3

Category 1 Berries and berry products continued

Product	Manufacturer / product label / country of origin	Procured in	Antioxidant content in mmol/100g	Comment
Chokeberries, black, wild	Norway	Norway	13.48	4
Cloudberries, wild	Norway	Norway	2.53	4
Cloudberries, wild, frozen	Sweden	Norway	3.44	3
Cranberries, cultivated		USA	3.29	6
Cranberries, dried	NaturataSpielberger AG	Norway	2.03	3
Cranberries, dried	USA, Eurocompany srl, Godo, Italy	Norway	1.90	3
Cranberries, dried	Safeway, USA	USA	1.64	3
Cranberry craisins cherry flavor	Ocean Spray	USA	1.47	3
Cranberry, syrup, without sugar (undiluted)	Corona, Norway	Norway	1.93	3
Crowberries	Skjervøy, Norway	Norway	7.89	3
Crowberries	Norway	Norway	2.48	4
Crowberries	Norway	Norway	8.45	4
Crowberries, frozen	Norway	Norway	10.80	4
Crowberry, syrup (undiluted)	Skjervøy, Norway	Norway	2.05	3
Crowberry, syrup, juice of peel (undiluted)	Midnattsol Produkter, Norway	Norway	4.50	3
Crowberry, syrup, without sugar (undiluted)	Corona, Norway	Norway	2.95	3
Crowberry, syrup, without sugar (undiluted)	Midnattsol Produkter, Norway	Norway	5.90	3
Dog Rose	Norway	Norway	25.86	4
Dog Rose	Lørenskog, Norway	Norway	12.65	4
Dog Rose	Setcases in Spain	Spain	14.16	4
Dog Rose	Norway	Norway	34.49	4
Dog Rose, dried, whole	Helios, Norway	Norway	78.09	3
Dog Rose, powder	Chile	Norway	54.30	3
Dog Rose, powder, HybenMax	Fennomer Norge, Norway	Norway	20.82	3
Dog Rose, powder, Hyben-Vital	SunVita A/S, Denmark	Norway	28.49	3
Dog Roseshell, flour	Risenta, Finland	Norway	75.84	3
○Elderberries, black (berries from common elder), wild	Norway	Norway	5.24	4
Elderberries, cultivated	Norwegian University of Life Sciences	Norway	3.37	5

Category 1 Berries and berry products continued

Product	Manufacturer / product label / country of origin	Procured in	Antioxidant content in mmol/100g	Comment
Elderberry flowerdrink, concentrate		Norway	0.06	3
Elderberry, syrup, without sugar (undiluted)	Helios, Norway	Norway	6.31	5
Goji Berries, organic, dried	Natural Food Market Erewhon, grown in China	USA	4.31	
Gooseberries, cultivated	Norway	Norway	1.45	3
Juice, blackberry, juice/syrup from canned blackberries	Nora, Stabburet, Norway	Norway	1.27	3
Juice, cranberry	Tine, Norway	Norway	0.75	3
Juice, cranberry	Ocean Spray	USA	1.00	3
Juice, cranberry, juice cocktail	Roche Bros	USA	0.96	3
Juice, cranberry, juice cocktail	Stop&Shop	USA	0.86	3
Juice, cranberry, juice cocktail	Ocean Spray	USA	1.01	6
Juice, Mana Blå, grape, blueberry, aronia, cherry	Tine, Norway	Norway	2.00	3
Juice, Mana Gul, dog rose and orange	Tine, Norway	Norway	2.57	3
Juice, Mana Rød, cranberries, raspberries and grapes	Tine, Norway	Norway	0.67	3
Juice, raspberry	Bræmhults, Sweden	Norway	0.78	3
Juice, strawberry	Bræmhults, Sweden	Norway	0.43	3
Raspberries, cultivated		USA	2.33	6
Raspberries, cultivated	Norway	Norway	3.35	3
Raspberries, Dessert Berries, without sugar, frozen	Norske Dessertbær, Norway (berries from Poland)	Norway	3.46	3
Raspberries, wild	Malawi	Malawi	1.73	4
Raspberries, wild	Norway	Norway	3.97	4
Raspberries, wild	Fruitmaster, Netherlands	Norway	2.50	3
Raspberry, jam	Nora, Stabburet, Norway	Norway	1.26	3
Raspberry, jam,	Eldorado, Norgesgruppen, Norway	Norway	1.12	3

Category 1 Berries and berry products continued

Product	Manufacturer / product label / country of origin	Procured in	Antioxidant content in mmol/100g	Comment
Raspberry, jam, Bestemor Lerum Bringebærsyltetøy	Lerum Konserves, Norway	Norway	1.40	3
Raspberry, jam, light	Nora, Stabburet, Norway	Norway	1.27	3
Red whortleberries, cultivated, dried	The Norwegian Crop Research Institute, Norway	Norway	32.28	5
Red whortleberries, frozen	Poland	Norway	4.98	3
Red whortleberries, syrup, without sugar (undiluted)	Corona, Norway	Norway	2.34	3
Red whortleberries, wild	Norway	Norway	5.00	4
Redcurrant, cultivated	Norway	Norway	1.61	3
Redcurrant, Dessert Berries, without sugar, frozen	Norske Dessertbær, Norway (berries from Poland)	Norway	1.87	3
Rock bramble, frozen, wild	Norway	Norway	8.51	4
Rowanberries, dried	The Norwegian Crop Research Institute, Norway	Norway	19.13	5
Rowanberries, dried	Norsk Øko-Urt BA, Norway	Norway	10.13	5
Rowanberries, wild	Norway	Norway	2.36	4
Sea buckthorn, berries	Denmark	Denmark	2.21	4
Sea buckthorn, berries	Norway	Norway	4.58	4
Sour cherries, cultivated	Norway	Norway	7.14	3
Sour cherries, without stone, without sugar, frozen	Poland	Norway	3.30	3
Sour cherries, without stone, without sugar, frozen	Norway	Norway	6.07	4
Strawberries, Corona, cultivated	Norway	Norway	2.05	3
Strawberries, cultivated		USA	2.16	6
Strawberries, ”Honey,” cultivated	Norwegian University of Life Sciences	Norway	2.33	5
Strawberries, ”Senga Sengana”, cultivated	Norwegian University of Life Sciences	Norway	1.85	5
Strawberry, jam	Nora, Stabburet, Norway	Norway	0.64	3
Strawberry, jam, light	Nora, Stabburet, Norway	Norway	0.68	3
Sweet cherries, cultivated	Norway	Norway	1.42	3

Category 1 Berries and berry products continued

Product	Manufacturer / product label / country of origin	Procured in	Antioxidant content in mmol/100g	Comment
Wild strawberries	Norway	Norway	5.44	4
Zereshk (Barberries, dried)	Iran	Iran	27.30	3

Category 2 Beverages

Product	Manufacturer / product label / country of origin	Procured in	Antioxidant content in mmol/100g	Comment
Aquavit, Løiten Linie	Løiten Brænderis Destillation, Norway	Norway	0.12	3
Beer, Bavarian Weizen	Haandbryggeriet, Norway	Norway	0.27	3
Beer, Bayer	Frydenlund, Norway	Norway	0.25	3
Beer, Beck`s beer	Brauerei Beck & CO, Germany	Norway	0.13	3
Beer, Guinnes Draught	Guinness UDV, Irland	Norway	0.21	3
Beer, Lager Beer	Heineken, Netherlands	Norway	0.13	3
Beer, Leffe	Br.Abbaye de Leffe, Belgium	Norway	0.22	3
Beer, light	Bud Light	USA	0.08	6
Beer, light	Mixed brands	USA	0.10	6
Beer, light	Natural Light	USA	0.10	6
Beer, light	Miller Light	USA	0.09	6
Beer, light	Coors Light	USA	0.06	6
Beer, Limfjords Porter, double brown stout	Thisted Bryghus, Denmark	Norway	0.43	3
Beer, Lysholmer Spesial	E.C. Dahls bryggeri, Norway	Norway	0.18	3
Beer, Mack Bok-øl	Macks Ølbryggeri, Norway	Norway	0.43	3
Beer, Maredsous, Trippel 10	Belgisch Abdjbier, Belgium	Norway	0.42	3
Beer, Pale ale	Nøgne Ø, Norway	Norway	0.31	3
Beer, Pères Trappistes	Chimay, Belgium	Norway	0.33	3
Beer, Pils	Ringnes, Norway	Norway	0.19	3
Beer, Pils	Tuborg, Denmark	Norway	0.22	3
Beer, Porter	Nøgne Ø, Norway	Norway	0.46	3
Beer, Premium Lager	Grolsch	Norway	0.21	3
Beer, regular	Mixed brands	USA	0.14	6
Beer, regular	Busch	USA	0.12	6
Beer, regular	Miller High Life	USA	0.12	6
Beer, regular	Budweiser	USA	0.15	6

Category 2 Beverages continued

Product	Manufacturer / product label / country of origin	Procured in	Antioxidant content in mmol/100g	Comment
Beer, Saison Dupont, Biologique	Belgium	Norway	0.29	3
Beer, Samichlaus Bier (Santa Claus Beer), Lager Beer	Castle Brewery Eggenberg, Austria	Norway	0.46	3
Beer, without alcohol	Munkholm, Norway	Norway	0.11	3
Beer, Aass Gull	Aass Bryggerier, Norway	Norway	0.19	3
Blackcurrant, toddy, instant, Regia	Freia, Norway	Norway	2.64	3
Blackcurrant, toddy, instant, Regia, prepared	Freia, Norway	Norway	0.38	3
Blåbærsmust	Glanshammars	Sweden	3.28	3
Brandy, Napoleon V.S.O.P.	St.Remy Machecoul, France	Norway	0.22	3
Cider, Ãpple, 4,5vol%	Halmstad, Sweden	Norway	0.02	3
Cider, Pãron, 2,25vol%	Halmstad, Sweden	Norway	0.02	3
Cockburn`s Port	Cockburn`s Port, Portugal	Norway	0.60	3
Coffee beans, green	Solberg & Hansen, Norway	Norway	20.18	3
Coffee beans, green	Turkey	Norway	12.30	3
Coffee beans, raw/green	Minas	Norway	15.19	3
Coffee beans, roasted (black)	Turkey	Norway	22.29	3
Coffee beans, roasted, Monsooned Malabar AA	Solberg & Hansen, Norway	Norway	22.73	3
Coffee, Ali, dark burned ground, 100% Arabica, filter brewed	Joh. Johannson Kaffe, Norway	Norway	2.28	3
Coffee, Ali, ground, 100% Arabica, boiled	Joh. Johannson Kaffe, Norway	Norway	2.23	3
Coffee, Ali, ground, 100% Arabica, filter brewed	Joh. Johannson Kaffe, Norway	Norway	2.61	3
Coffee, Andes Bolivia, boiled	Hervik, Norway	Norway	2.62	3
Coffee, Andes Bolivia, filter brewed	Hervik, Norway	Norway	2.55	3
Coffee, Arabica green, filter brewed	CIRAD, France	France	1.55	5
Coffee, Arabica medium roasting, filter brewed	CIRAD, France	France	2.78	5

Category 2 Beverages continued

Product	Manufacturer / product label / country of origin	Procured in	Antioxidant content in mmol/100g	Comment
Coffee, Arabica mild roasting, filter brewed	CIRAD, France	France	2.45	5
Coffee, Arabica strong roasting, filter brewed	CIRAD, France	France	2.69	5
Coffee, boiled	Kaffehuset Friele, Norway	Norway	2.18	3
Coffee, Cafe Organico Arabica, instant, prepared	Simon Levelt by Haarlem	Norway	2.20	3
Coffee, Caffe Latte, double, prepared	Kaffebrenneriet, Norway	Norway	1.96	3
Coffee, Caffe Latte, double, prepared	Stockflehts, Norway	Norway	1.36	3
Coffee, Caffe Latte, single, prepared	Kaffebrenneriet, Norway	Norway	0.94	3
Coffee, Caffe Latte, single, prepared	Stockflehts, Norway	Norway	0.89	3
Coffee, Cappucino, double, prepared	Kaffebrenneriet, Norway	Norway	2.75	3
Coffee, Cappucino, double, prepared	Stockflehts, Norway	Norway	2.86	3
Coffee, Cappucino, single, prepared	Stockflehts, Norway	Norway	1.04	3
Coffee, Cappucino, single, prepared	Kaffebrenneriet, Norway	Norway	2.14	3
Coffee, Cirkel Coffee, boiled	Joh. Johannson Kaffe, Norway	Norway	1.79	3
Coffee, Cirkel Coffee, ground coffee, filter brewed	Joh. Johannson Kaffe, Norway	Norway	2.13	3
Coffee, Espresso Originale Italiano, classico, prepared	Cellini	Norway	2.70	3
Coffee, Espresso, double, prepared	Stockflehts, Norway	Norway	16.33	3
Coffee, Espresso, single, prepared	Stockflehts, Norway	Norway	15.83	3
Coffee, Espresso, single, prepared	Kaffebrenneriet, Norway	Norway	12.64	3
Coffee, Evergood, boiled	Joh. Johannson Kaffe, Norway	Norway	1.84	3
Coffee, Evergood, caffein free, ground, filter brewed	Joh. Johannson Kaffe, Norway	Norway	2.39	3
Coffee, Evergood, filter brewed	Joh. Johannson Kaffe, Norway	Norway	2.52	3

Category 2 Beverages continued

Product	Manufacturer / product label / country of origin	Procured in	Antioxidant content in mmol/100g	Comment
Coffee, Farmers Coffee (fairtrade Max Havelaar), filter brewed	Joh. Johannson Kaffe, Norway	Norway	2.58	3
Coffee, filter brewed	McDonald's	Norway	1.86	3
Coffee, Finca Rodomunho, boiled	Hervik, Norway	Norway	2.10	3
Coffee, Finca Rodomunho, filter brewed	Hervik, Norway	Norway	2.25	3
Coffee, Frokost, filter brewed	Kaffehuset Friele, Norway	Norway	3.03	3
Coffee, Iced coffee Cappucino, ready to drink	Tine, Norway	Norway	0.93	3
Coffee, Iced coffee Mocca, ready to drink	Tine, Norway	Norway	1.05	3
Coffee, instant, Nescafe Gull, prepared	Nestlé, Norway	Norway	1.67	3
Coffee, instant, prepared	Coop, Norway	Norway	1.60	3
Coffee, instant, Sombrero, prepared	Coop, Norway	Norway	1.68	3
Coffee, Lavazza, Caffe Espresso, 100% Arabica, prepared	Luigi Lavazza, Italy	Norway	2.83	3
Coffee, L'Or, 100% Arabica, boiled	Maison du Cafe, France	Norway	3.03	3
Coffee, L'Or, 100% Arabica, filter brewed	Maison du Cafe, France	Norway	3.34	3
Coffee, Macciato, double, prepared	Kaffebrenneriet, Norway	Norway	5.03	3
Coffee, Macciato, double, prepared	Stockflehts, Norway	Norway	11.30	3
Coffee, Macciato, single, prepared	Stockflehts, Norway	Norway	6.48	3
Coffee, Macciato, single, prepared	Kaffebrenneriet, Norway	Norway	5.32	3
Coffee, prepared	Kaffebrenneriet, Norway	Norway	4.20	3
Coffee, prepared	Stockflehts, Norway	Norway	3.03	3
Coffee, prepared	Burger King	USA	1.26	6
Coffee, prepared	Wendy's	USA	1.24	6
Coffee, Robusta green, filter brewed	CIRAD, France	France	2.99	5

Category 2 Beverages continued

Product	Manufacturer / product label / country of origin	Procured in	Antioxidant content in mmol/100g	Comment
Coffee, Robusta medium roasting, filter brewed	CIRAD, France	France	3.09	5
Coffee, Robusta mild roasting, filter brewed	CIRAD, France	France	3.60	5
Coffee, Robusta strong roasting, filter brewed	CIRAD, France	France	2.25	5
Coffee, yellow Coop, filter brewed	Coop, Norway	Norway	3.11	3
Cognac, V.S.Martell, Fine Cognac	France	Norway	0.32	3
Cognac, X.O.Braastad, FineChampagne	France	Norway	0.45	3
Cordial, lemon flavored, Fun light, Sitron, undiluted	Stabburet, Norway	Norway	0.40	3
Cordial, orange flavored, Fun Light Appelsin, undiluted	Stabburet, Norway	Norway	0.45	3
Cordial, peach flavored, Fun light, Peach Passion, undiluted	Stabburet, Norway	Norway	0.36	3
Cordial, raspebbery flavored, Fun light, Bringebær, undiluted	Stabburet, Norway	Norway	0.62	3
Cordial, strawberry flavored, Fun light Jordbær, undiluted	Stabburet, Norway	Norway	0.45	3
Cordial, wild berries flavored, Fun light Wild Berries, undiluted	Stabburet, Norway	Norway	0.15	3
Diet Mountain Dew Superb		USA	0.00	3
Energy drink	Red Bull	USA	0.00	6
Energy drink, sugar free	Red Bull	USA	0.00	6
Gingerale	E.C. Dahls bryggeri, Norway	Norway	0.01	3
Gingerale, american type	Ringnes, Norway	Norway	0.00	3
Instant cocoa, Choco-Mocca, powder	Confecta, Norway	Norway	5.83	3
Instant cocoa, Choco-Mocca, prepared	Confecta, Norway	Norway	0.33	3
Instant cocoa, Regia express, light, powder	Freia, Norway	Norway	4.96	3
Instant cocoa, Regia express, light, prepared	Freia, Norway	Norway	0.42	3

Category 2 Beverages continued

Product	Manufacturer / product label / country of origin	Procured in	Antioxidant content in mmol/100g	Comment
Instant cocoa, Regia express, original, powder	Freia, Norway	Norway	3.09	3
Instant cocoa, Regia express, original, prepared	Freia, Norway	Norway	0.45	3
Instant cocoa, Rett i Koppen, powder	Toro, Norway	Norway	1.61	3
Instant cocoa, Rett i Koppen, prepared	Toro, Norway	Norway	0.26	3
Juice drinks, 10% juce, boppin' strawberry flavor	Hi C	USA	0.72	6
Juice drinks, 10% juice, splash cooler flavor	Capri Sun	USA	0.03	6
Juice drinks, 10% juice, strawberry kiwi flavor	Capri Sun	USA	0.05	6
Juice drinks,10% ,strawberry flavor	Capri Sun	USA	0.06	6
Juice drinks,10% juice, blazin' blueberry flavor	Hi C	USA	0.73	6
Juice drinks,10% juice, mountain cooler flavor	Capri Sun	USA	0.02	6
Latino beverages, guanabana nectar	Goya	USA	0.21	6
Latino beverages, guanabana nectar	Other Brand	USA	0.28	6
Latino beverages, guava (guayaba) nectar	Other Brand	USA	0.87	6
Latino beverages, mango nectar	Goya	USA	0.20	6
Latino beverages, mango nectar	Other Brand	USA	0.60	6
Latino beverages, tamarind (tamarindo) nectar	Other Brand	USA	0.36	6
Latino beverages, tamarind (tamarindo) nectar	Goya	USA	0.16	6
Lemonade mix, pink, sweetened with artificial sweetener	Crystal Light	USA	0.00	6
Lemonade mix, sweetened with artificial sweetener	Crystal Light	USA	0.00	6
Lemonade powder mix, pink, sweetened with sugar	Country Time	USA	0.77	6

Category 2 Beverages continued

Product	Manufacturer / product label / country of origin	Procured in	Antioxidant content in mmol/100g	Comment
Lemonade powder mix, sweetened with sugar	Kool Aid	USA	0.61	6
Lemonade powder mix, sweetened with sugar	Country Time	USA	0.85	6
Lemonade powder mix, unsweetened	Kool Aid	USA	12.75	6
Lemonade, lemon	Nora, Stabburet, Norway	Norway	0.14	3
Lemonade, mixed wild berries	Nora, Stabburet, Norway	Norway	0.33	3
Lemonade, pink, frozen concentrate	Other Brand	USA	0.46	6
Lemonade, pink, frozen concentrate	Minute Maid	USA	0.43	6
Lemonade, regular, frozen concentrate	Store Brand	USA	0.45	6
Lemonade, regular, frozen concentrate	Minute Maid	USA	0.37	6
Liquor of crowberries, Frost	Arcus	Norway	0.42	3
Malt beer	Ringnes, Norway	Norway	0.26	3
Nectar, Multivitamine	Carrefour	USA	0.59	3
Non-carbonated bottled drinking water	Aquafina	USA	0.00	6
Non-carbonated bottled drinking water	Evian	USA	0.00	6
Non-carbonated bottled drinking water	Store Brand	USA	0.00	6
Non-carbonated bottled drinking water	Dannon	USA	0.00	6
Non-carbonated bottled drinking water	Calistoga	USA	0.00	6
Non-carbonated bottled drinking water	Naya	USA	0.00	6
Non-carbonated bottled drinking water	Crystal Geyser	USA	0.00	6
Non-carbonated bottled drinking water	Dasani	USA	0.00	6
Non-carbonated flavored bottled drinking water, elements enhanced water energy, lemon	Snappple	USA	0.07	6

Category 2 Beverages continued

Product	Manufacturer / product label / country of origin	Procured in	Antioxidant content in mmol/100g	Comment
Non-carbonated flavored bottled drinking water, essential multi vitamin, watermelon flavor	Aquafina	USA	0.02	6
Non-carbonated flavored bottled drinking water, fitness H2O, natural lemon flavor	Propel	USA	0.11	6
Non-carbonated flavored bottled drinking water, fluoride to go	Dannon	USA	0.00	6
Non-carbonated flavored bottled drinking water, fruit2o water, natural strawberry flavor	Very fine	USA	0.00	6
Non-carbonated flavored bottled drinking water, fruit2o, natural raspberry flavor	Very fine	USA	0.00	6
Prune drink	Nestlé	Norway	1.79	3
Ricoffy, instant coffee & chicory, Nescafe, powder	Nestlé, South Africa	Malawi	51.86	3
Soft drink, citrus fruit, Urge	Coca Cola Drikker	Norway	0.08	3
Soft drink, cola, diet	Burger King	USA	0.05	6
Soft drink, cola, diet	Pepsi	USA	0.04	6
Soft drink, cola, diet	Coke	USA	0.04	6
Soft drink, cola, diet	Wendy's	USA	0.03	6
Soft drink, cola, diet	McDonald's	USA	0.02	6
Soft drink, cola, diet	Pepsi One	USA	0.06	6
Soft drink, cola, regular	Pepsi	USA	0.04	6
Soft drink, cola, regular	Pepsi Co Nordic Norway AS	Norway	0.04	3
Soft drink, cola, regular	Coca-Cola Company	USA	0.05	6
Soft drink, cola, regular	Wendy's	USA	0.02	6
Soft drink, cola, regular	Coca Cola Drikker	Norway	0.04	3
Soft drink, cola, regular	Burger King	USA	0.03	6
Soft drink, cola, regular	McDonald's	USA	0.00	6
Soft drink, Fanta	Coca Cola Drikker	Norway	0.08	3
Soft drink, lemon-lime, light, 7 UP	Ringnes, Norway	Norway	0.00	3
Soft drink, lemon-lime, regular, Sprite	Coca Cola Company	USA	0.00	6

Category 2 Beverages continued

Product	Manufacturer / product label / country of origin	Procured in	Antioxidant content in mmol/100g	Comment
Soft drink, lemon-lime, Sprite	Coca-Cola Drikker	Norway	0.00	3
Soft drink, orange, Solo	Ringnes, Norway	Norway	0.13	3
Soft drink, white soda, regular	McDonald's	USA	1.27	6
Spirulina Cocktail	Bræmhults, Sweden	Norway	0.38	3
Sports drink mix, orange flavor	Gatorade	USA	0.01	6
Sports drink, lemon-lime flavor	Powerade	USA	0.01	6
Sports drink, orange flavor	Gatorade	USA	0.00	6
Tang, Jamaica, drinking powder	Mexico	Mexico	2.05	3
Tang, Naranja, drinking powder	Mexico	Mexico	0.54	3
Tang, Tamarino, drinking powder	Mexico	Mexico	1.12	3
Tea, black, Ceylon Breakfast, prepared	Solberg & Hansen, Norway	Norway	0.75	3
Tea, black, Ceylon, prepared		New Zealand	1.21	3
Tea, black, Darjeeling Tiger Hill, prepared		Norway	1.12	3
Tea, black, Earl Grey, prepared	Twinings, England	Norway	0.95	3
Tea, black, Earl Grey, prepared		Norway	0.82	3
Tea, Camomile flowers, prepared	Dihlma	New Zealand	0.10	3
Tea, Combe Tea, dried	The Foods Company Ltd, Malawi	Malawi	57.57	3
Tea, Darjeeling, leaves, dried	Roopaks, Ajmal Khan, N. Dehli	India	2.93	3
Tea, dog rose, from dried dogrose, prepared	Helios, Norway	Norway	0.20	3
Tea, dog rose, from extract, prepared	Weiders Farmasøytiske A/S, Norway	Norway	2.49	3
Tea, dog rose, herb tea, prepared		Norway	0.31	3
Tea, Flor de Jamaica, prepared	Mexico	Mexico	6.99	3

Category 2 Beverages continued

Product	Manufacturer / product label / country of origin	Procured in	Antioxidant content in mmol/100g	Comment
Tea, fruit, Mango, prepared	Twinings, England	Norway	0.98	3
Tea, fruit, wild berries, prepared	Solberg & Hansen, Norway	Norway	0.49	3
Tea, green, (pink) powder	NPS, Japan	Norway	1347.83	3
Tea, green, Assam TGFOP, prepared	Solberg & Hansen, Norway	Norway	1.49	3
Tea, green, dried		Norway	24.31	3
Tea, green, Earl Grey, prepared	Twinings, England	Norway	1.43	3
Tea, green, Emperors Garden, prepared	Tetly Australia PtyLtd	New Zealand	1.36	3
Tea, green, Green Java, prepared	Twinings, England	Norway	2.62	3
Tea, green, Green Label, dried	Lipton	India	6.77	3
Tea, green, Gunpowder, prepared	China	Norway	1.31	3
Tea, green, Gunpowder, prepared	Solberg & Hansen, Norway	Norway	1.19	3
Tea, green, Jasmin, prepared	Lipton	Norway	1.27	3
Tea, green, Jasmine, prepared	Twinings, England	Norway	1.93	3
Tea, green, Jasmine, prepared	Dilhma	New Zealand	2.11	3
Tea, green, Java Green, prepared	Twinings, England	Norway	2.31	3
Tea, green, leaves, dried, Kashmir Kahawa	Ajmal Khan, N. Dehli	India	10.13	3
Tea, green, Ling ching, prepared	Solberg & Hansen, Norway	Norway	1.26	3
Tea, green, mint, prepared	Lipton	Norway	0.88	3
Tea, green, Monkey, prepared	China	Norway	0.57	3
Tea, green, Sencha, prepared	Solberg & Hansen, Norway	Norway	1.24	3
Tea, green, Spesial Chun Mee, prepared	Solberg & Hansen, Norway	Norway	1.43	3
Tea, herb, Mate-brown, prepared		Norway	0.27	3
Tea, iced tea, brewed, unsweetened	Wendy's	USA	0.37	6
Tea, iced tea, peach	Eldorado	Norway	0.46	3

Category 2 Beverages continued

Product	Manufacturer / product label / country of origin	Procured in	Antioxidant content in mmol/100g	Comment
Tea, iced tea, ready to drink, brisk lemon iced tea	Lipton	USA	0.05	6
Tea, iced tea, ready to drink, iced tea with lemon flavor	Arizona	USA	0.16	6
Tea, iced tea, ready to drink, natural lemon iced tea	Nestea	USA	0.05	6
Tea, instant, dry powder, unsweetened	Nestea	USA	165.86	6
Tea, instant, dry powder, with lemon and sugar, prepared	Lipton	USA	0.94	6
Tea, leaves, fresh	Malawi	Malawi	26.55	4
Tea, Peppermint, ground and whole fresh leaves, prepared		Norway	0.43	3
Tea, Rabs Classic Malawi Tea, dried	Rab Processors Ltd, Malawi	Malawi	57.72	3
Tea, Roiboos, Aspalathus linearis, prepared	Freshpak, South Africa	South Africa	0.42	
Tea, Rooibos, prepared	South-Africa	Norway	0.61	3
Tea, Sermoni, green tea, prepared		Norway	2.45	3
Tea, Sermoni, powder		Norway	155.42	3
Vanilla shake, national	Burger King	USA	0.08	6
Whisky, Finest Scotch Whisky	The Famouse Grouse, Gloug & Son, Scotland	Norway	0.10	3
White soda, regular	Wendy's	USA	0.00	6
White soda, regular	Burger King	USA	0.00	6
Wine, Danish Cherry-wine		England	0.23	3
Wine, red, Amigo		Norway	2.15	3
Wine, red, Arrow 2000	USA	Norway	2.44	3
Wine, red, Banrock Station Shiraz		Norway	1.97	3
Wine, red, Baro`n de Oña toija 1996	Spain	Norway	2.03	3
Wine, red, Boheme		Norway	2.06	3
Wine, red, Canaletto 2000	Italy	Norway	3.05	3
Wine, red, Canepa 2000	Chile	Norway	2.82	3
Wine, red, Capella	Puglia, Italy	Norway	2.49	3
Wine, red, Cato Negro		Norway	2.49	3

Category 2 Beverages continued

Product	Manufacturer / product label / country of origin	Procured in	Antioxidant content in mmol/100g	Comment
Wine, red, Chanti Collisensi 2003		Norway	3.08	3
Wine, red, Chapelle Du Bois		Norway	1.94	3
Wine, red, Chateau Coufran1999	France	Norway	2.62	3
Wine, red, Chianti Classico 2000	Italy	Norway	2.68	3
Wine, red, Côte de Nuits-Villages1998	France	Norway	1.78	3
Wine, red, Crozes Hermitage 1999	France	Norway	2.24	3
Wine, red, Fortino	Italy	Norway	2.55	3
Wine, red, J.P.Chenet		Norway	2.83	3
Wine, red, Kimberly 2001	South-Africa	Norway	2.33	3
Wine, red, La Boiselière		Norway	1.82	3
Wine, red, La Buvette	France	Norway	2.37	3
Wine, red, Merlot		USA	2.13	6
Wine, red, Mezzamonde Negramoro		Norway	2.90	3
Wine, red, Montepulciano 2001	Italy	Norway	3.66	3
Wine, red, Syrah, bag-in-box	France	Norway	2.10	3
Wine, red, Terriero 2001	Italy	Norway	2.41	3
Wine, red, Vidigal Reserva		Norway	2.69	3
Wine, red, Vino Maipo		Norway	2.70	3
Wine, white, Black Tower		Norway	0.19	3
Wine, white, Blue Nun		Norway	0.37	3
Wine, white, Caliterra 2000	Chile	Norway	0.30	3
Wine, white, Chardonnay		USA	0.16	6
Wine, white, Dr. L. Riesling		Norway	0.25	3
Wine, white, Dunvar		Norway	0.44	3
Wine, white, Liebfraumilch Silbervin		Norway	0.50	3
Wine, white, Milestone 2001	Australia	Norway	0.47	3
Wine, white, Moscato		Norway	0.42	3
Wine, white, Moselland		Norway	0.38	3
Wine, white, Moselland Ars Vitis		Norway	0.40	3
Wine, white, Müller Reiler Wom Heissen Stein		Norway	0.30	3
Wine, white, Tarquet		Norway	0.50	3
Wine, white, Viogner		Norway	0.56	3

Category 3 Breakfast cereals

Product	Manufacturer / product label / country of origin	Procured in	Antioxidant content in mmol/100g	Comment
100% Bran	Post	USA	1.20	3
100% Natural Granola, Oats, Honey, & Raisins	Quaker	USA	0.16	6
All-Bran	Kellogg's	USA	1.68	3
All-Bran	Kellogg's	USA	1.56	6
All-Bran Plus	Kellogg's Company of Great Britain Ltd. For nordic Kellogg's Norway	Norway	1.68	3
All-Bran Regular	Kellogg's Company of Great Britain Ltd. For nordic Kellogg's Norway	Norway	1.96	3
Bran Flakes	Post	USA	1.21	3
Bran Flakes	Ralston	USA	4.29	6
Cap'n Crunch Peanut Butter Cereal	Quaker	USA	0.53	6
Cheerios	General Mills, USA	USA	1.12	6
Cheerios	General Mills, USA	USA	1.09	3
Cheerios	Nestlé, Norway	Norway	0.97	3
Cocoa Krispies	Kellogg's	USA	1.56	6
Cocos-frokost, chocolate flavor	Kellogg's GmbH. For Nordic Kellogg`s Norway	Norway	0.88	3
Complete	Kellogg's	USA	4.84	3
Corn and Rice, Crispix	Kellogg's	USA	1.13	6
Corn and Rice, Crispy Doubles	Giant Eagle	USA	0.72	6
Corn and Rice, Crispy Hexagons	Shop 'N Save	USA	0.88	6
Corn and Rice, Crispy Hexagons	Price Chopper	USA	0.78	6
Corn Chef	Corn Chef	USA	1.18	3
Corn Flakes	Ralston	USA	1.23	6
Corn Flakes	Toasties	USA	0.85	3
Corn Flakes	Kellogg's	USA	1.28	6
Corn Flakes	Kellogg's GmbH, Germany	Norway	0.98	3
Corn Flakes	Landlord, Germany	Norway	0.70	3

Category 3 Breakfast cereal continued

Product	Manufacturer / product label / country of origin	Procured in	Antioxidant content in mmol/100g	Comment
Corn Flakes, ecological	Germany	Norway	0.62	3
Corn Flakes, Honey Crunch	Kellogg's GmbH. For Kellogg's Norway	Norway	0.66	3
Corn Squares, Corn Biscuits	Ralston	USA	0.46	6
Corn Squares, Toasted Corn	Price Chopper	USA	0.50	6
Crispix	Kellogg's	USA	0.74	3
Crusli	Quaker Oats B.V., Netherlands	USA	0.52	3
Crusli, Solfrokost	Quaker Oats B.V., Netherlands	USA	0.59	3
Energy Mix	Quaker Oats B.V., Netherlands	USA	0.45	3
Fiber One	General Mills, USA	USA	2.17	3
Fitness	Nestlé, Norway	Norway	0.99	3
Fitness & Fruits	Nestlé, Norway	Norway	0.53	3
Froot Loops	Kellogg's	USA	0.90	6
Frosted Flakes	Kellogg's	USA	0.89	3
Frosted Mine Wheets	Kellogg's	USA	0.23	3
Grape Nuts Flakes		USA	0.88	3
Grear grains, Pecan Crunch	Post	USA	1.09	3
Honey Bunckes of Oats	Post	USA	0.34	3
Honey Nut Cheerios	General Mills, USA	USA	1.04	3
Just Right	Kellogg's	USA	0.58	3
Kix	GMI	USA	0.55	3
Life	Quaker	USA	0.36	3
Loops, Multi Grain	Kellogg's GmbH. For Kellogg's Norway	Norway	0.53	3
Low fat Granola	Kellogg's	USA	0.53	3
Mueslix	Kellogg's	USA	0.64	3
Multi-Grain Chex		USA	1.97	3
Müsli Whole Grain	Euro Shopper, Germany	Norway	0.41	3
Müsli, Frokost	Landlord, Germany	Norway	0.48	3
Müsli, Frokost	Nordkronen, Norway	Norway	0.66	3
Nabisco Shredded	Nabisco	USA	0.44	3
Oat Circle	Ralston	USA	0.84	6
Oat squares	Nakskov Mil FoodsA/S Denmark for Quaker Oats Scandinavia	Norway	1.14	3
Oat, puffed	Hakon, Norway	Norway	2.11	3

Category 3 Breakfast cereal continued

Product	Manufacturer / product label / country of origin	Procured in	Antioxidant content in mmol/100g	Comment
Oat, puffed	Norgesmøllen DA, Norway	Norway	1.89	3
Oat, puffed	Gaute Næringsmiddelindustri, Norway	Norway	1.79	3
Old Glory	Kellogg's	USA	1.05	3
Original shredded wheat	Post	USA	0.23	6
Paisin Bran	Post	USA	0.73	3
Product 19	Kellogg's	USA	4.01	3
Puffed Rice	Quaker	USA	0.43	3
Puffed Rice	Cub Foods	USA	0.29	3
Puffed Wheat	Cub Foods	USA	1.26	3
Raisin Bran	Kellogg's	USA	1.16	3
Raisin Bran	Post	USA	0.77	6
Raisin Bran	Malt O`meal	USA	0.75	3
Rice Chex		USA	0.37	3
Rice Crisp	Kellogg's	USA	0.86	6
Rice Crisp	Ralston	USA	1.02	6
Rice Krispies	Kellogg's	USA	0.88	3
Rice Puffs	Cub Foods	USA	0.24	3
Rice, puffed	Hakon, Norway	Norway	0.30	3
Shredded Wheat	Kraft	USA	0.24	3
Shredded Wheat	Post	USA	0.22	3
Special	Kellogg's Company of Great Britain Ltd. For nordic Kellogg`s Norway	Norway	0.90	3
Special K	Kellogg's	USA	1.56	3
Special K	Kellogg's	Norway	1.83	3
Squares	Quaker	USA	1.03	3
Squares, with cinnamon	Quaker	USA	1.01	3
Total	General Mills, USA	USA	2.51	3
Uncle Sam Cereal	US Mills	USA	1.12	6
Weetabix	Weetabix Ltd, England	USA	1.30	3
Weetos, with chockolate flavor	Weetabix Ltd, England	USA	0.80	3
Wheat Chex	General Mills, USA	USA	1.17	3
Wheat Puffs	Cub Foods	USA	1.64	3
Wheaties	General Mills, USA	USA	1.67	3
Whole Grain Total	General Mills, USA	USA	3.41	6

Category 4 Chocolate and sweets

Product	Manufacturer / product label / country of origin	Procured in	Antioxidant content in mmol/100g	Comment
Cacao, for baking, powder, Regia	Freia, Norway	Norway	13.74	3
Candy bars	Tootsie Rolls	USA	1.40	6
Candy bars, Snickers		USA	0.73	6
Candy bars, Wafer Bar	Kit Kat	USA	0.72	6
Caramel, creem, Smørbukk	Nidar, Norway	Norway	1.31	3
Caramel, lemon, FOX	Malaco	Norway	0.11	3
Chocolate	Plamil Foods Limited , England	Norway	3.08	3
Chocolate butter, Sjokade	Sunda, Norway	Norway	1.15	3
Chocolate butter, Sjokella	Nestlé, Norway	Norway	0.73	3
Chocolate, Cacao Gastronomie, 100%	Valrhona	Norway	11.22	3
Chocolate, chocolate covered cacao nibs, 53% cocoa	Chocolate Santander, Colombia	Norway	13.29	3
Chocolate, chocolate covered coffe beans, 53% cocoa	Chocolate Santander, Colombia	Norway	12.62	3
Chocolate, chocolate discs, 70% cocoa	Chocolate Santander, Colombia	Norway	14.98	3
Chocolate, dark chocolate, Colombian coffee bits, 70% cocoa	Chocolate Santander, Colombia	Norway	14.79	3
Chocolate, dark chocolate, Columbian singel origin, 70% cocoa	Chocolate Santander, Colombia	Norway	13.56	3
Chocolate, dark chocolate,Columbian singel origin with cacao nibs, 70% cocoa	Chocolate Santander, Colombia	Norway	14.47	3
Chocolate, dark, 70% cocoa	Freia, Norway	Norway	7.83	3
Chocolate, dark, 70% cocoa	Lindt & Sprungli, France	Norway	13.44	3
Chocolate, dark, Bocca Dark	Nidar, Norway	Norway	7.67	3

Category 4 Chocolate and sweets continued

Product	Manufacturer / product label / country of origin	Procured in	Antioxidant content in mmol/100g	Comment
Chocolate, dark, for baking, Mørk Kokesjokolade	Hakon, Norway	Norway	9.04	3
Chocolate, dark, Lindt Excellence extra fine, 85% cocoa	Lindt & Sprüngli, France	Norway	13.58	3
Chocolate, dark, Lindt Exellence 99% cocoa	Lindt & Sprüngli, France	Norway	12.09	3
Chocolate, dark, Noir Amer, Guanaja, 70% cocoa	Valrhona	Norway	10.74	3
Chocolate, dark, Noir Dark, Lindt Excellence, 70% cocoa	Lindt & Sprüngli, France	Norway	8.38	3
Chocolate, dark, Noir de Domiane, Ampamakia, 64% cocoa	Valrhona	Norway	10.33	3
Chocolate, dark, Noir de Domiane, Chuao, 65% cocoa	Valrhona	Norway	12.26	3
Chocolate, dark, Noir de Domiane, Gran Couva, 64% cocoa	Valrhona	Norway	7.64	3
Chocolate, dark, Noir, 72% cocoa	Villars	Norway	11.00	3
Chocolate, dark, Sensation Brut Noir de Noir, 86% cocoa	Côte d'Or	Norway	11.67	3
Chocolate, dark, Sensation intense Noir de Noir, 70% cocoa	Côte d'Or	Norway	9.03	3
Chocolate, dark, Superior Dark 72% cocoa	Confecta, Norway	Norway	11.35	3
Chocolate, dark, Toblerone	Kraft Foods, Switzerland	Norway	1.71	3
Chocolate, Dronning	Freia, Norway	Norway	4.73	3
Chocolate, for baking, Lys Kokesjokolade	Freia, Norway	Norway	3.59	3
Chocolate, for baking, unsweetened	Bakers, Norway	USA	7.28	6
Chocolate, for baking, unsweetened	Hershey's	USA	10.47	6
Chocolate, Kvikk Lunsj	Freia, Norway	Norway	1.48	3
Chocolate, milk chocolate	Euro Shopper	Norway	1.44	3
Chocolate, milk chocolate candy	Cadbury	USA	1.95	6

Category 4 Chocolate and sweets continued

Product	Manufacturer / product label / country of origin	Procured in	Antioxidant content in mmol/100g	Comment
Chocolate, milk chocolate candy	Brach's Stars	USA	1.25	6
Chocolate, milk chocolate candy	Hershey's	USA	1.55	6
Chocolate, milk chocolate candy, Hershey Kisses	Hershey's	USA	1.50	6
Chocolate, milk chocolate peanut butter cups, miniatures	Reese's	USA	1.07	1, 6
Chocolate, milk chocolate, Freia Melkesjokolade	Freia, Norway	Norway	5.06	3
Chocolate, Milky Way	Masterfoods, Norway	Norway	0.73	3
Chocolate, Mokkabønner	Nidar, Norway	Norway	6.23	3
Chocolate, New Energy	Nidar, Norway	Norway	1.20	3
Chocolate, Noir Nestlé Dessert	Nestlé, Norway	Norway	7.87	3
Chocolate, peanut butter cup miniatures, sugar-free	Reese's	USA	1.08	1, 6
Chocolate, Selskapssjokolade	Freia, Norway	Norway	4.94	3
Chocolate, semi-dark, Colombian Singel Origin, 53% cocoa	Chocolate Santander, Colombia	Norway	7.40	3
Chocolate, Snickers	Masterfoods, Norway	Norway	0.80	3
Chocolate, sugar-free, chocolate candy	Hershey's	USA	2.57	6
Chocolate, sugar-free, dark chocolate candy	Hershey's	USA	4.19	6
Chocolate, sweet authentic mexican chocolate	Ibarra, Mexico	Norway	8.83	3
Chocolate, Voll-Nuss (with whole hazelnuts)	Ritter Sport, Germany	Norway	0.62	3
Chocolate, white, with coconut	Lindt & Sprungli, France	Norway	0.23	3
Chocolate, with hazelnuts, Bocca Dark	Nidar, Norway	Norway	5.31	3
Chocolate, with pieces of hazelnuts, Firkløver	Freia, Norway	Norway	1.20	3
Chocolate, with whole hazelnuts, Helnøtt	Freia, Norway	Norway	0.82	3

Category 4 Chocolate and sweets continued

Product	Manufacturer / product label / country of origin	Procured in	Antioxidant content in mmol/100g	Comment
Coconut, sweetened, flaked	Store Brand	USA	0.09	6
Cononut, sweetened, flaked	Other Brand	USA	0.09	6
Cononut, sweetened, flaked, Angel Flake	Bakers, Norway	USA	0.10	6
Granola bars, 100% natural crunchy oats and honey	Nature Valley	USA	0.83	6
Granola bars, chewy, chocolate chip	Sunbelt	USA	0.76	6
Granola bars, chewy, chocolate chip	Quaker	USA	0.36	6
Granola bars, chewy, chocolate chip	Store Brand	USA	0.60	6
Granola bars, chewy, oats and honey	Sunbelt	USA	0.40	6
Granola bars, crunchy	Store Brand	USA	0.78	6
Liquorice candy, Godt og blandet	Malaco	Norway	4.71	3
Liquorice candy, NOX	Malaco	Norway	1.25	3
Liquorice candy, Skipper lakrisbåter	Nidar, Norway	Norway	0.69	3
Marzipan	Rainbow, Denmark	Norway	0.05	3
Marzipan, luxury quality	Odense, Denmark	Norway	0.05	3
Milk chocolate peanut butter cups	Reese's	USA	0.96	1, 6
Nut and chocolate butter, Nugatti	Sunda, Norway	Norway	1.10	3
Power bar, chocolate flavor		USA	2.76	6
Slim-Fast Meal options, Chewy Granola, chocolate chip, artificially flavored	Slim-Fast Foods co, USA	Norway	1.96	3
Slim-Fast Meal options, milk chocolate peanut flavored, meal on-the-go	Slim-Fast Foods co, USA	Norway	2.10	3
Slim-Fast Meal options, oatmeal raisin, natural & artificially flavored,meal on-the-go	Slim-Fast Foods co, USA	Norway	3.55	3

Category 5 Dairy and dairy products

Product	Manufacturer / product label / country of origin	Procured in	Antioxidant content in mmol/100g	Comment
Buttermilk, skimmed	Tine, Norway	Norway	0.05	3
Buttermilk, special 1.5% fat, Biola	Tine, Norway	Norway	0.05	3
Buttermilk, special 1.5% fat, Cultura	Tine, Norway	Norway	0.04	3
Cheese, american cheese/pasteurized process cheese	Kraft Singles	USA	0.06	6
Cheese, american cheese/pasteurized process cheese food	Kraft Deluxe	USA	0.04	6
Cheese, american cheese/pasteurized process cheese food	Store Brand	USA	0.06	6
Cheese, american/skim, white, sliced, commodity	Land O Lakes	USA	0.03	6
Cheese, american/skim, yellow, sliced	Schreiber	USA	0.04	6
Cheese, american/skim, yellow, sliced, commodity	Land O Lakes	USA	0.04	6
Cheese, Brie Coeur de Lion	France	Norway	0.22	1, 3
Cheese, brown goat cheese	Tine, Norway	Norway	0.78	3
Cheese, Cheddar, chunk	Store Brand	USA	0.06	1, 6
Cheese, Cheddar, chunk	Kraft	USA	0.09	1, 6
Cheese, Gorgonzola dolce	Italy	Norway	0.54	1, 3
Cheese, Mozzarella, low moisture part skim	Store Brand	USA	0.06	1, 6
Cheese, Mozzarella, low moisture part skim	Kraft	USA	0.04	1, 6
Cheese, Mozzarella, whole milk	Precious	USA	0.12	1, 6
Cheese, Mozzarella, whole milk	Store Brand	USA	0.06	1, 6
Cheese, Mozzarella, whole milk	Polly-O	USA	0.10	1, 6
Cheese, Mozzarella, whole milk	Sorrento	USA	0.11	1, 6

Category 5 Diary and diary products continued

Product	Manufacturer / product label / country of origin	Procured in	Antioxidant content in mmol/100g	Comment
Cheese, Parmesan, grated	Kraft	USA	0.06	6
Cheese, Parmesan, grated	Store Brand	USA	0.10	6
Cheese, Philadelphia, original	Kraft	Norway	0.19	3
Cheese, processed, commodity	Land O Lakes	USA	0.06	6
Cheese, processed, white, sliced	AMPI	USA	0.03	6
Cheese, processed, yellow, sliced, commodity	Bongards	USA	0.04	6
Cheese, processed, yellow, sliced, commodity	Land O Lakes	USA	0.05	6
Cheese, Roquefort	France	Norway	0.43	1, 3
Cheese, St Agur	France	Norway	0.65	1, 3
Cheese, Stilton ring	England	Norway	0.54	1, 3
Cheese, Swiss Cheese, slices	Store Brand	USA	0.08	1, 6
Cheese, Swiss Cheese, slices	Kraft	USA	0.07	1, 6
Cheese, white, Norvegia	Tine, Norway	Norway	0.10	3
Cottage Cheese	Tine, Norway	Norway	0.05	3
Cream, 22% fat	Tine, Norway	Norway	0.06	3
Cream, 35% fat	Tine, Norway	Norway	0.07	3
Goatmilk	Tine, Norway	Norway	0.04	3
Ice cream	Burger King	USA	0.00	6
Ice cream, chocolate, regular fat	Store Brand	USA	0.50	6
Ice cream, chocolate, regular fat	Breyers	USA	0.76	6
Ice cream, chocolate, regular fat, grand chocolate flavor	Edy's/Dreyer's	USA	0.71	6
Ice cream, regular fat, natural vanilla	Edy's/Dreyer's	USA	0.05	6
Ice cream, regular fat, natural vanilla	Breyers	USA	0.06	6
Ice cream, vanilla	Nestlé, Norway	Norway	0.22	3
Ice cream, vanilla	Møvenpick, Switzerland	Norway	0.32	3
Ice cream, vanilla, regular fat	Other Brand	USA	0.07	6
Ice cream, vanilla, regular fat	Store Brand	USA	0.06	6
Ice cream, with strawberry	Hennig Olsen, Norway	Norway	0.21	3
Milk, 1%	USA	USA	0.05	6
Milk, 2%	USA	USA	0.04	6

Category 5 Diary and diary products continued

Product	Manufacturer / product label / country of origin	Procured in	Antioxidant content in mmol/100g	Comment
Milk, chocolate, 2% reduced fat	Hershey's	USA	0.17	6
Milk, chocolate, 2%, reduced fat	Store Brand	USA	0.14	6
Milk, extra semi-skimmed	Tine, Norway	Norway	0.04	3
Milk, Kefir	Tine, Norway	Norway	0.05	3
Milk, semi-skimmed	Tine, Norway	Norway	0.04	3
Milk, semi-skimmed, ecological	Tine, Norway	Norway	0.04	3
Milk, semi-skimmed, lactose reduced	Tine, Norway	Norway	0.05	3
Milk, semi-skimmed, Q-Melk	Gausdal Meieriet for Kavli, Norway	Norway	0.05	3
Milk, skim	USA	USA	0.04	6
Milk, skimmed	Tine, Norway	Norway	0.04	3
Milk, skimmed	MATFORSK, Norway	Norway	0.03	5
Milk, skimmed fermented	MATFORSK, Norway	Norway	0.05	5
Milk, skimmed fermented with blackcurrants	MATFORSK, Norway	Norway	0.27	5
Milk, skimmed fermented with blueberries	MATFORSK, Norway	Norway	0.42	5
Milk, skimmed with blueberries	MATFORSK, Norway	Norway	0.35	5
Milk, skimmed, Q -Melk	Gausdal Meieriet for Kavli, Norway	Norway	0.05	3
Milk, skimmed, with blackcurrants	MATFORSK, Norway	Norway	0.20	5
Milk, whole, H-Melk	Tine, Norway	Norway	0.04	3
Milk, whole, Q-Melk	Gausdal Meieriet for Kavli, Norway	Norway	0.04	3
Sour cream	Breakstone	USA	0.01	6
Sour cream	Store Brand	USA	0.06	3
Sour cream	Tine, Norway	Norway	0.15	3
Sour cream	Other Brand	USA	0.03	6
Sour cream, low fat	Tine, Norway	Norway	0.09	3
Vanilla milkshake	McDonald's	USA	0.12	6
Wendy's Ice	Wendy's	USA	0.00	6
Yogurt	Store Brand	USA	0.04	6
Yogurt, 99% fat free, strawberry	Yoplait	USA	0.11	6
Yogurt, frozen, chocolate	Other Brand	USA	0.45	6

Category 5 Diary and diary products continued

Product	Manufacturer / product label / country of origin	Procured in	Antioxidant content in mmol/100g	Comment
Yogurt, frozen, fat free, vanilla	Edy's/Dreyer’s	USA	0.06	6
Yogurt, frozen, vanilla	Other Brand	USA	0.05	6
Yogurt, mixed wild berries	Tine, Norway	Norway	0.25	3
Yogurt, original	Tine, Norway	Norway	0.06	3
Yogurt, prunes	Tine, Norway	Norway	0.11	3
Yogurt, strawberries	Tine, Norway	Norway	0.08	3
Yogurt, strawberry, fruit on the bottom	Dannon	USA	0.13	6

Category 6 Desserts and cakes

Product	Manufacturer / product label / country of origin	Procured in	Antioxidant content in mmol/100g	Comment
Apple pie	Berthas	Norway	0.19	3
Brownies	7 eleven	Norway	0.60	3
Bun, with cinnamon	ICA	Norway	0.50	3
Bun, with cinnamon and vanilla cream	ICA	Norway	0.37	3
Bun, with currants	Bakers, Norway	Norway	0.46	3
Cake frosting, chocolate, ready-to-spread, creamy chocolate	Pillsbury	USA	0.80	6
Cake frosting, chocolate, ready-to-spread, creamy homestyle classic chocolate	Duncan Hines	USA	0.92	6
Cake frosting, chocolate, ready-to-spread, creamy homestyle milk chocolate	Duncan Hines	USA	1.01	6
Cake frosting, chocolate, ready-to-spread, creamy milk chocolate	Pillsbury	USA	1.09	6
Cake frosting, chocolate, ready-to-spread, rich & creamy	Betty Crocker	USA	0.81	6
Cake frosting, vanilla, ready-to-spread, creamy	Pillsbury	USA	0.09	6
Cake frosting, vanilla, ready-to-spread, creamy homestyle	Duncan Hines	USA	0.04	6
Cake frosting, vanilla, ready-to-spread, rich & creamy	Betty Crocker	USA	0.15	6
Cake mix, chocolate devils food cake mix, dry, Super Moist	Betty Crocker	USA	0.69	6
Cake mix, chocolate devils food cake mix, dry, Moist Deluxe	Duncan Hines	USA	0.90	6
Cake mix, chocolate devils food cake mix, dry, Moist Supreme	Pillsbury	USA	0.98	6

Category 6 Desserts and cakes continued

Product	Manufacturer / product label / country of origin	Procured in	Antioxidant content in mmol/100g	Comment
Cake Mix, chocolate devils food cake mix, prepared, moist deluxe	Duncan Hines	USA	0.38	6
Cake mix, chocolate devils food cake mix, prepared, super moist	Betty Crocker	USA	0.43	6
Chocolate cake	Studentkafeen A/S, Norway	Norway	0.38	3
Chocolate cake, filled	Baker Nordby, Norway	Norway	0.80	3
Chocolate cake, filled, dark, Sachertorte	Take away bakery	Norway	0.71	3
Chocolate devils food cake mix, moist supreme, prepared	Pillsbury	USA	0.61	6
Chocolate muffin, with chocolate chips	7 eleven	Norway	0.93	3
Cini-Minis	Burger King	USA	0.61	6
Cookies, chocolate chip	Chunky Chips Ahoy	USA	2.23	6
Cookies, chocolate chip	Entenmann's	USA	1.38	6
Cookies, chocolate chip	Store Brand	USA	1.29	6
Cookies, chocolate chip	Chips Ahoy	USA	1.72	6
Cookies, chocolate chip	7 eleven	Norway	0.60	3
Cookies, chocolate chip	Chips Deluxe	USA	1.00	6
Cookies, chocolate chip cookies, with hazelnuts	The horizon Biscuit Company Limited, England	Norway	0.88	3
Cookies, chocolate with vanilla creme filling	Oreo	USA	1.84	6
Cookies, chocolate with vanilla creme filling	Store Brand	USA	1.56	6
Cookies, chocolate with vanilla creme filling, double stuf	Oreo	USA	1.54	6
Cupcakes, chocolate	Hostess	USA	1.15	6
Cupcakes, chocolate	Little Debbie	USA	0.97	6
Doughnuts with candy sprinkles	7 eleven	Norway	0.26	3
Doughnuts, cake, chocolate covered	Store Brand	USA	0.29	6

Category 6 Desserts and cakes continued

Product	Manufacturer / product label / country of origin	Procured in	Antioxidant content in mmol/100g	Comment
Doughnuts, cake, chocolate covered	Hostess Donettes	USA	0.87	6
Doughnuts, cake, plain	Store Bakery or prepackaged	USA	0.14	6
Doughnuts, chocolate-glazed	7 eleven	Norway	0.45	3
Doughnuts, glazed, plain	Store Brand	USA	0.18	6
Doughnuts, glazed, plain	Krispy Kreme	USA	0.16	6
Doughnuts, mini-cake-type with powdered sugar	Hostess Donettes	USA	0.11	6
Doughnuts, original glazed	7 eleven	Norway	0.32	3
Frosty dairy dessert	Wendy's	USA	0.16	6
Fruit dessert	Nora, Stabburet, Norway	Norway	0.29	3
Fruit dessert, red	Nora, Stabburet, Norway	Norway	0.38	3
Ice-type novelties, containing fruit juice	Popsicle Scribblers	USA	0.10	6
Ice-type novelties, regular, cherry flavor	Popsicle Ice Bar	USA	0.01	6
Ice-type novelties, regular, grape flavor	Popsicle Ice Bar	USA	0.01	6
Ice-type novelties, regular, orange flavor	Popsicle Ice Bar	USA	0.01	6
Ice-type novelties, sugar free, cherry flavor	Popsicle Ice Bar	USA	0.09	6
Ice-type novelties, sugar free, grape flavor	Popsicle Ice Bar	USA	0.09	6
Ice-type novelties, sugar free, orange flavor	Popsicle Ice Bar	USA	0.09	6
Jello, snack, strawberry flavor	Hunts Juicy Gels	USA	0.03	6
Jello, snack, strawberry flavor	Jell-O	USA	0.00	6
Jello, snack, strawberry/orange flavor	Jell-O	USA	0.01	6
Jello, snack, strawberry/orange flavor	Hunts Juicy Gels	USA	0.01	6
Jello, with gooseberry flavor, powder, prepared	Freia, Norway	Norway	0.00	3
Jello, with strawberry flavor	Tine, Norway	Norway	0.05	3

Category 6 Desserts and cakes continued

Product	Manufacturer / product label / country of origin	Procured in	Antioxidant content in mmol/100g	Comment
Muffin with blueberries	7 eleven	Norway	0.46	3
Muffin with blueberries	Store Brand	USA	0.46	6
Muffin with blueberries, Mini Muffins	Hostess	USA	0.37	6
Pancakes, buttermilk frozen, microwave	Hungry Jack	USA	0.17	6
Pancakes, buttermilk, frozen, microwaved	Aunt Jemima	USA	0.07	6
Pancakes, buttermilk, frozen, microwaved	Eggo	USA	0.10	6
Pancakes, buttermilk, frozen, toasted	Eggo	USA	0.13	6
Pancakes, buttermilk, frozen, toasted	Hungry Jack	USA	0.27	6
Pancakes, buttermilk, frozen, uncooked	Hungry Jack	USA	0.23	6
Pancakes, buttermilk, frozen, uncooked	Eggo	USA	0.14	6
Pie crust, frozen deep dish, regular fat	Store Brand	USA	0.05	6
Pie crust, frozen deep dish, regular fat	Marie Callender's	USA	0.01	6
Pie crust, frozen deep dish, regular fat	Pilsbury Pet-Ritz	USA	0.05	6
Pie crust, frozen deep dish, regular fat, baked	Store Brand	USA	0.15	6
Pie crust, frozen deep dish, regular fat, baked	Pilsbury Pet-Ritz	USA	0.21	6
Pie crust, frozen deep dish, regular fat, baked	Marie Callender's	USA	0.17	6
Pie crust, frozen, regular fat	Store Brand	USA	0.08	6
Pie crust, frozen, regular fat, baked	Store Brand	USA	0.31	6
Prune dessert	Nora, Stabburet, Norway	Norway	0.67	3
Pudding mix, chocolate, cook & serve	Jell-O	USA	2.24	6
Pudding mix, chocolate, sugar free, cook & serve	Jell-O	USA	4.10	6
Pudding mix, chocolate, sugar-free, fat-free instant	Jell-O	USA	2.74	6

Category 6 Desserts and cakes continued

Product	Manufacturer / product label / country of origin	Procured in	Antioxidant content in mmol/100g	Comment
Pudding mix, vanilla, sugar-free, fat-free instant	Jell-O	USA	0.37	6
Pudding mix, vanilla, cook & serve	Jell-O	USA	0.14	6
Pudding mix, vanilla, instant	Jell-O	USA	0.13	6
Pudding mix, vanilla, sugar free, cook & serve	Jell-O	USA	0.75	6
Pudding, caramel, Piano, ready-to-eat	Tine, Norway	Norway	0.03	3
Pudding, chocolate, Piano, ready-to-eat	Tine, Norway	Norway	0.35	3
Pudding, handi snacks pudding, vanilla flavor	Kraft	USA	0.05	6
Pudding, handi snacks, chocolate flavor	Kraft	USA	0.41	6
Pudding, refrigerated fat free, snack pack, tapioca flavor	Jell-O	USA	0.08	6
Pudding, refrigerated, snack pack, chocolate flavor	Jell-O	USA	0.40	6
Pudding, refrigerated, snack pack, chocolate flavor	Swiss Miss	USA	0.31	6
Pudding, refrigerated, snack pack, vanilla flavor	Jell-O	USA	0.02	6
Pudding, refrigerated, tapioca flavor	Swiss Miss	USA	0.04	6
Pudding, refrigerated, vanilla flavor	Swiss Miss	USA	0.06	6
Pudding, snack pack, chocolate flavor	Hunt's	USA	0.26	6
Pudding, snack pack, tapioca flavor	Hunt's	USA	0.03	6
Pudding, snack pack, vanilla flavor	Hunt's	USA	0.06	6
Soup, dog rose	Ekströms	Norway	2.79	3
Toaster pastries, strawberry, frosted	Store Brand	USA	0.18	6
Toaster pastries, strawberry, frosted	Kellogg's Pop Tarts	USA	0.15	6
Toaster pastries, strawberry, frosted, toasted	Store Brand	USA	0.28	6

Category 6 Desserts and cakes continued

Product	Manufacturer / product label / country of origin	Procured in	Antioxidant content in mmol/100g	Comment
Toaster pastries, strawberry, frosted, toasted	Kellogg's Pop Tarts	USA	0.28	6
Toaster pastries, strawberry, plain (not frosted)	Store Brand	USA	0.17	6
Toaster pastries, strawberry, plain (not frosted)	Kellogg's Pop Tarts	USA	0.16	6
Toaster pastries, strawberry, plain, toasted (not frosted)	Store Brand	USA	0.31	6
Waffels	Baker Nordby, Norway	Norway	0.21	3
Waffles, buttermilk, frozen	Aunt Jemima	USA	0.12	6
Waffles, buttermilk, frozen	Eggo	USA	0.08	6
Waffles, buttermilk, frozen, microwaved	Eggo	USA	0.07	6
Waffles, buttermilk, frozen, microwaved	Aunt Jemima	USA	0.12	6
Waffles, buttermilk, frozen, toasted	Downyflake	USA	0.16	6
Waffles, buttermilk, frozen, toasted	Store Brand	USA	0.20	6
Waffles, buttermilk, frozen, toasted	Eggo	USA	0.11	6
Waffles, buttermilk, frozen, toasted	Hungry Jack	USA	0.20	6
Waffles, regular, frozen	Eggo	USA	0.07	6
Waffles, regular, frozen	Store Brand	USA	0.10	6
Waffles, regular, frozen microwaved	Eggo	USA	0.06	6
Waffles, regular, frozen, microwaved	Store Brand	USA	0.09	6
Waffles, regular, frozen, toasted	Store Brand	USA	0.17	6
Waffles, regular, frozen, toasted	Aunt Jemima	USA	0.17	6
Waffles, regular, frozen, toasted	Downyflake	USA	0.16	6
Waffles, regular, frozen, toasted	Eggo	USA	0.11	6

Category 7 Egg

Product	Manufacturer / product label / country of origin	Procured in	Antioxidant content in mmol/100g	Comment
Egg, beaters	Beatrice Foods	USA	0.00	3
Egg, scrambled, with milk	Prior, Norway	Norway	0.08	3
Egg, whites	Prior, Norway	Norway	0.00	3
Egg, whites		USA	0.01	6
Egg, whole	Prior, Norway	Norway	0.06	3
Egg, whole	Store Brand	USA	0.04	6
Egg, whole		USA	0.02	6
Egg, whole, fried		USA	0.05	6
Egg, whole, hard cooked		USA	0.04	6
Egg, yolk	Prior, Norway	Norway	0.16	3
Egg, yolk	Norgården	Norway	0.05	3
Egg, yolk		USA	0.02	6

Category 8 Fats and oils

Product	Manufacturer / product label / country of origin	Procured in	Antioxidant content in mmol/100g	Comment
Butter	Tine, Norway	Norway	0.73	1, 3
Butter, Tine smør, Ekte Meierismør	Tine, Norway	Norway	0.36	1, 3
Canola and corn oil blend	Store Brand	USA	0.29	2, 6
Canola and corn oil blend	Mazola Right Blend	USA	0.19	2, 6
Canola oil	Store Brand	USA	0.39	2, 6
Canola oil	Crisco	USA	0.48	2, 6
Canola oil	Mazola	USA	0.46	2, 6
Canola oil	Wesson	USA	0.51	2, 6
Canola oil, cold-pressed	AFB Askim Frukt- og Bærpresseri, Norway	Norway	0.44	2, 3
Corn oil	Mazola	USA	0.34	2, 6
Corn oil	Store Brand	USA	0.33	2, 6
Corn oil	Euro Shopper	Norway	0.49	2, 3
Margarine, Brelett Oliven, light	Fjordland, Norway	Norway	0.44	1, 3
Margarine, Brelett, light	Fjordland, Norway	Norway	0.52	1, 3
Margarine, Bremykt	Fjordland, Norway	Norway	0.40	1, 3
Margarine, Melange	Mills, Norway	Norway	1.50	1, 3
Margarine, Per	A/S Margarinfabrikken, Norway	Norway	1.66	1, 3
Margarine, Soft Flora	Mills, Norway	Norway	1.38	1, 3
Margarine, Soft light	Mills, Norway	Norway	1.00	1, 3
Margarine, Soya	A/S Margarinfabrikken, Norway	Norway	1.53	1, 3
Olive oil	Store Brand	USA	0.23	2, 6
Olive oil, extra virgin	Bertolli	USA	0.25	2, 6
Olive oil, extra virgin	Eldorado	Norway	0.41	1, 3
Olive oil, extra virgin	Gaea, Greece	Norway	0.29	2, 3
Olive oil, extra virgin	Ybarra, Spain	Norway	0.36	2, 3
Olive oil, extra virgin	Pompeian	USA	0.31	2, 6
Olive oil, extra virgin, Fraticello	Pietro Coricelli, Italy	Norway	0.19	2, 3

Category 8 Fats and oils continued

Product	Manufacturer / product label / country of origin	Procured in	Antioxidant content in mmol/100g	Comment
Olive oil, extra virgin, Kalamata D.O.P.	Gaea, Greece	Norway	0.31	2, 3
Olive oil, extra virgin, Sitia-crete D.O.P.	Gaea, Greece	Norway	0.28	2, 3
Olive oil, Kalamata extra virgin, golden selection	Iliada, Greece	Norway	0.29	2, 3
Olive oil, Kalamata extra virgin, organic farming	Iliada, Greece	Norway	0.25	2, 3
Soy bean oil	Mills, Norway	Norway	0.47	2, 3
Soy bean oil	Mazola Vegetable Oil	USA	0.35	2, 6
Soy bean oil	Store Brand	USA	0.53	2, 6
Soy bean oil	Wesson Vegetable Oil	USA	0.43	2, 6
Sunflower oil	Euro Shopper	Norway	0.33	2, 3
Vegetable oil	Store Brand	USA	0.34	2, 6
Vegetable oil	Crisco	USA	0.41	2, 6

Category 9 Fish and seafood

Product	Manufacturer / product label / country of origin	Procured in	Antioxidant content in mmol/100g	Comment
Crab, canned	Bumble Bee	USA	0.12	6
Fish sticks, breaded, frozen, baked, Crunchy Fish Sticks	Van De Kamp's	USA	0.08	6
Fish sticks, breaded, frozen, baked, Crunchy Golden Fish Sticks	Gortons	USA	0.07	6
Fish sticks, breaded, frozen, baked, Select Cuts Crunchy Fish Sticks	Mrs Paul's	USA	0.08	6
Fish sticks, breaded, frozen, Crunchy Fish Sticks	Van De Kamp's	USA	0.07	6
Fish sticks, breaded, frozen, Crunchy Golden Fish Sticks	Gorton's	USA	0.06	6
Fish sticks, breaded, frozen, Select Cuts Crunchy Fish Sticks	Mrs Paul's	USA	0.07	6
Kapenta, dried	Wopanada McHere, Malawi	Malawi	0.65	3
Mackerel, fried	Norway	Norway	0.12	3
Mackerel, raw	Norway	Norway	0.11	3
Orange roughy, fillets	USA	USA	0.03	6
Orange roughy, fillets, baked	USA	USA	0.04	6
Pollock burger, fried	Fiskern Maritime Produkter, Norway	Norway	0.20	3
Pollock filet, raw	Norway	Norway	0.04	3
Prawns, peeled, cooked	Reinhartsen Seafood, Denmark	Norway	0.24	3
Salmon, pink, canned with skin and bones	Other Brand	USA	0.08	6
Salmon, pink, canned with skin and bones	Bumble Bee	USA	0.07	6
Salmon, pink, canned with skin and bones	Chicken of the Sea	USA	0.07	6
Salmon, pink, canned with skin and bones	Store Brand	USA	0.07	6
Salmon, raw	Norway	Norway	0.03	3

Category 9 Fish and seafood continued

Product	Manufacturer / product label / country of origin	Procured in	Antioxidant content in mmol/100g	Comment
Salmon, Red/Sockeye, canned with skin and bones	Bumble Bee	USA	0.10	6
Salmon, Red/Sockeye, canned with skin and bones	Chicken of the Sea	USA	0.08	6
Salmon, Red/Sockeye, canned with skin and bones	Other Brand	USA	0.06	6
Salmon, Red/Sockeye, canned with skin and bones	Store Brand	USA	0.13	6
Shrimp, canned, cooked	Orleans or Bumblebee	USA	0.04	6
Tilapia, fillets, baked	USA	USA	0.14	6
Tilapia, fillets, raw	USA	USA	0.08	6
Tuna, canned, chunk, light, in water	Bumble Bee	USA	0.10	6
Tuna, canned, chunk, light, in water	Star Kist	USA	0.09	6
Tuna, canned, chunk, light, in water	Store Brand	USA	0.12	6
Tuna, canned, in oil	Diva	Norway	0.21	3
Tuna, canned, in water	Chicken of the Sea	USA	0.09	6

Category 10 Fruit and fruit juices

Product	Manufacturer / product label / country of origin	Procured in	Antioxidant content in mmol/100g	Comment
Apples, Composite of Red Delicious, Golden Delicious, Granny Smith, Gala, & Fuji,		USA	0.31	6
Apples, dried	Eldorado, Germany	Norway	1.86	3
Apples, dried	Fruit Zone, Horizon Food, UK	Norway	3.49	3
Apples, dried (Tasmanian dried apples)	Australia	New Zealand	6.07	3
Apples, Fuji		USA	0.22	6
Apples, Gala		USA	0.25	6
Apples, Gala	Italy	Norway	0.22	3
Apples, Golden Delicious	New Zealand	Norway	0.15	3
Apples, Golden Delicious		USA	0.26	6
Apples, Golden Delicious, without peel		USA	0.10	6
Apples, Granny Smith		USA	0.54	6
Apples, Granny Smith	Le Crunch	Norway	0.51	3
Apples, green, Greenstar	Fruitmaster, Netherlands	Norway	1.22	3
Apples, red, Fuji	China	Norway	0.40	3
Apples, red, Pink Lady	VOG Terlano, Italy	Norway	0.35	3
Apples, red, Pinova	Coop, Italy	Norway	0.57	3
Apples, red, Red Delicious		USA	0.40	6
Apples, red, Red Delicious, ecological	Friland, Italy	Norway	0.48	3
Apples, red, Red Delicious, without peel		USA	0.08	6
Apricots		Norway	0.52	3
Apricots, canned, drained	Diva	Norway	0.14	3
Apricots, dried	India	India	1.32	3
Apricots, dried	Sunsweet, Turkey	Norway	3.23	3
Apricots, dried	Buyers Choice	New Zealand	4.67	3
Apricots, dried	Diva	Norway	3.23	3
Banana		USA	0.34	6
Banana	Del Monte	Norway	0.27	3
Banana	Mali	Mali	0.08	3

Category 10 Fruit and fruit juices continued

Product	Manufacturer / product label / country of origin	Procured in	Antioxidant content in mmol/100g	Comment
Cherries		USA	0.35	6
Cherries, sour, canned, heavy syrup, total can contents	Del Monte	USA	1.65	6
Cherries, sour, canned, water pack, drained liquid	Oregon and Kroger	USA	1.66	6
Cherries, sour, canned, water pack, drained liquid	Kroger and Red Tart	USA	1.72	6
Cherries, sour, canned, water pack, drained solids	Oregon and Kroger	USA	2.01	6
Cherries, sour, canned, water pack, drained solids	Kroger and Red Tart	USA	2.04	6
Clementines		USA	0.74	6
Clementines		Norway	0.99	3
Clementines	Gamma, Spania	Norway	0.75	3
Clementines	Cevita	Norway	0.95	3
Currant (raisins of Korinth)		Norway	0.67	3
Dates		Norway	1.04	3
Dates	Mali	Mali	0.95	3
Dates, Deglet Noor		USA	0.72	6
Dates, dried	Mali	Mali	1.53	3
Dates, dried, Chuhare	India	India	1.88	3
Dates, Medjool		USA	0.56	6
Figs	Turkey	Norway	0.73	3
Figs		USA	0.78	6
Figs, dried	Sunsweet, Turkey	Norway	0.75	3
Figs, dried	Smyrna, Turkey	Norway	0.76	3
Figs, dried	Dessert Maid, Australia	New Zealand	0.42	3
Figs, dried	India	India	1.08	3
Figs, dried, Calimyrna	Sun Maid, USA	Norway	1.83	3
Figs, dried, Mission	Sun Maid, USA	Norway	1.31	3
Fruit from the African Baobab tree	Malawi	Malawi	10.84	4
Fruit salad, traditional, in natural juice	Golden Cicle, Australia	New Zealand	0.36	3
Glacè cherries	Home Brand	New Zealand	0.14	3
Grapefruit, red		USA	0.59	6
Grapefruit, red	Dole	Norway	0.83	3
Grapefruit, yellow	Jaffa	Norway	0.82	3

Category 10 Fruit and fruit juices continued

Product	Manufacturer / product label / country of origin	Procured in	Antioxidant content in mmol/100g	Comment
Grapes, blue	Chiquita, Chile	Norway	0.69	3
Grapes, blue	Carmel, Israel	Norway	2.42	3
Grapes, blue	Del Monte	Norway	0.90	3
Grapes, blue, Don Mario	Italy	Norway	0.78	3
Grapes, blue, Salvi	Puglia, Italy	Norway	0.80	3
Grapes, green		USA	0.13	6
Grapes, green	Italy	Norway	0.41	3
Grapes, green, Mario de Cristo	Italy	Norway	0.30	3
Grapes, green, Salvi	Italy	Norway	0.24	3
Grapes, green, without stone	Turkey	Norway	0.18	3
Grapes, red		USA	0.32	6
Grapes, red	Italy	Norway	0.47	3
Guava		Norway	1.21	3
Guava, wild	Malawi	Malawi	1.18	4
Honeydew		USA	0.12	6
Ice, sorbet, mango	Møvenpick, Switzerland	Norway	0.11	3
Jam, apple	Nora, Stabburet, Norway	Norway	0.86	3
Jam, apple	Lerum, Norway	Norway	0.77	3
Japanese plum pulp, paste		Japan	0.45	3
Juice from canned apricots	Hervik, Norway	Norway	0.14	3
Juice, 4 frukter (4 fruits)	Nora, Stabburet, Norway	Norway	0.49	3
Juice, apple	Hakon, Norway	Norway	0.12	3
Juice, apple	Fellesmeieriet, Norway	Norway	0.17	3
Juice, apple	Very fine	USA	0.36	3
Juice, apple	Roche Bros	USA	0.20	3
Juice, apple	Meierienes, Norway	Norway	0.19	3
Juice, apple	Mott's	USA	0.41	3
Juice, apple	Lerum, Norway	Norway	0.17	3
Juice, apple	Ølen safteri, Norway	Norway	0.60	3
Juice, apple	Nora, Stabburet, Norway	Norway	0.16	3
Juice, apple, calcium enriched, with added vitamin C	Minute Maid	USA	0.72	6

Category 10 Fruit and fruit juices continued

Product	Manufacturer / product label / country of origin	Procured in	Antioxidant content in mmol/100g	Comment
Juice, apple, God Frokost	Nora, Stabburet, Norway	Norway	0.16	3
Juice, apple, premium	Meierienes, Norway	Norway	0.47	3
Juice, apple, with added vitamin C	Juicy Juice	USA	0.71	6
Juice, apple, with added vitamin C	Mott's	USA	0.34	6
Juice, apple, with added vitamin C	Dole	USA	0.59	6
Juice, apple, with dietary fibre	Nora, Stabburet, Norway	Norway	0.22	3
Juice, Cranapple	Ocean Spray	USA	0.71	6
Juice, fruits with pomegranate	Nora, Stabburet, Norway	Norway	0.72	3
Juice, grape	Fellesmeieriet, Norway	Norway	1.50	3
Juice, grape and grape blends	Welch's	USA	1.62	6
Juice, grape and grape blends, sweetened	Minute Maid	USA	0.87	6
Juice, grape and grape blends, unsweetened	Mott's	USA	0.69	6
Juice, grape and grape blends, unsweetened	Juicy Juice	USA	0.87	6
Juice, grape, purple	Welch's	USA	1.74	3
Juice, grapefruit	Roche Bros	USA	1.06	3
Juice, grapefruit	Mills, Norway	Norway	0.67	3
Juice, grapefruit, red, with pulp	Nora, Stabburet, Norway	Norway	0.55	3
Juice, lemon	Mali	Mali	0.33	3
Juice, mango and pineapple	Bræmhults, Sweden	Norway	0.34	3
Juice, orange	Farmer’s Fairtrade	Norway	0.60	3
Juice, orange	Bræmhults, Sweden	Norway	0.81	3
Juice, orange	Delights	Norway	0.47	3
Juice, orange	Mills, Norway	Norway	0.84	3
Juice, orange	Nora, Stabburet, Norway	Norway	0.61	3
Juice, orange	Fellesmeieriet, Norway	Norway	0.68	3
Juice, orange	Eldorado	Norway	0.71	3

Category 10 Fruit and fruit juices continued

Product	Manufacturer / product label / country of origin	Procured in	Antioxidant content in mmol/100g	Comment
Juice, orange	Mali	Mali	0.81	3
Juice, orange and carrot	Bræmhults, Sweden	Norway	0.29	3
Juice, orange and pineapple	Bræmhults, Sweden	Norway	0.53	3
Juice, orange and pineapple with pulp	Nora, Stabburet, Norway	Norway	0.51	3
Juice, orange and strawberry	Bræmhults, Sweden	Norway	0.76	3
Juice, orange, from concentrate	Dean	USA	0.58	6
Juice, orange, from concentrate	Minute Maid	USA	0.58	6
Juice, orange, from concentrate, with added vitamin C	Minute Maid	USA	0.76	6
Juice, orange, from Florida, with Omega-3 from plant	Nora, Stabburet, Norway	Norway	0.57	3
Juice, orange, frozen concentrate	Store Brand	USA	2.39	6
Juice, orange, frozen concentrate, country style	Minute Maid	USA	2.36	6
Juice, orange, frozen concentrate, original	Minute Maid	USA	2.51	6
Juice, orange, frozen concentrate, pulp free	Minute Maid	USA	2.35	6
Juice, orange, red	Bræmhults, Sweden	Norway	1.38	3
Juice, orange, refrigerated	Store Brand	USA	0.57	6
Juice, orange, refrigerated, premium	Minute Maid	USA	0.59	6
Juice, orange, refrigerated, pure premium	Tropicana	USA	0.56	6
Juice, orange, with pulp	Nora, Stabburet, Norway	Norway	0.65	3
Juice, orange, with pulp	Hakon, Norway	Norway	0.64	3
Juice, orange, with pulp	Mills, Norway	Norway	0.81	3
Juice, orange, with pulp	Fellesmeieriet, Norway	Norway	0.55	3
Juice, orange, with pulp, from Florida	Nora, Stabburet, Norway	Norway	0.60	3
Juice, pineapple	Fellesmeieriet, Norway	Norway	0.19	3
Juice, pineapple	S&W Fine Food, USA	Norway	0.42	3

Category 10 Fruit and fruit juices continued

Product	Manufacturer / product label / country of origin	Procured in	Antioxidant content in mmol/100g	Comment
Juice, pineapple, canned or bottled, unsweetened	Dole	USA	0.84	6
Juice, pomegranate, freshly squeezed from whole pomegranate	Spain	Norway	2.57	3
Juice, prune	Roche Bros	Norway	1.14	3
Juice, prune	Sunsweet	Norway	0.83	3
Juice, prune, with pulp	Sunsweet	Norway	1.10	3
Juice, Tropisk, original	Meierienes, Norway	Norway	0.22	3
Juice, Vie Shot, apple, carrot, strawberry	Knorr	Denmark	1.96	5
Juice, Vie Shot, banana, pumpkin, kiwi	Knorr	Denmark	1.00	5
Juice, white grape	Welch's	USA	1.14	3
Juice, white grape	Stop&Shop	USA	0.74	3
Juice, with fruits and berries, Noras Antioksidanter med bøåbær og bringebær	Nora, Stabburet, Norway	Norway	1.69	
Kiwano	Delica (NZ) LTD	Norway	0.05	3
Kiwi		USA	1.02	6
Kiwi, Gold		USA	1.63	6
Kiwi, green		Norway	0.43	3
Kiwi, green	Zespri, New Zeland	Norway	1.02	3
Kiwi, yellow	Zespri, New Zeland	Norway	1.29	3
Lemon	Argentina	Norway	0.56	3
Lemon	DANA D`OR, Spain	Norway	1.02	3
Lemon skin	Argentina	Norway	2.74	3
Lemon skin, from lemon ecologically grown	South Africa	Norway	4.00	3
Lemon, ecologically grown	South Africa	Norway	0.70	3
Lemonade syrup, Blackthorn, undiluted	Helios	Norway	2.44	3
Lemonade, grape (ready to drink)	Helios, Germany	Norway	1.27	3
Lime		USA	0.47	6
Lime	Brasil	Norway	0.58	3
Lime	Netherlands	Norway	0.73	3
Lime skin	Brasil	Norway	3.05	3
Mango	Dole	Norway	0.33	3
Mango	Mali	Mali	0.23	3

Category 10 Fruit and fruit juices continued

Product	Manufacturer / product label / country of origin	Procured in	Antioxidant content in mmol/100g	Comment
Mango, dried	Northwest Delights, USA	USA	0.58	3
Mango, dried	India	India	2.82	3
Mango, red	Mexico	Norway	0.37	3
Mango, yellow	Pakistan	Norway	0.36	3
Melon, Cantaloupe	Rose, Spain	Norway	0.19	3
Melon, Cantaloupe, small	Nolem, Brazil	Norway	0.12	3
Melon, pattern	Mali	Mali	0.15	3
Melon, yellow	Mali	Mali	0.29	3
Nectar, apple	Eldorado	Norway	0.11	3
Nectar, apple	Mills, Norway	Norway	0.14	3
Nectar, apple with pear	Nora, Stabburet, Norway	Norway	0.08	3
Nectar, orange	Nora, Stabburet, Norway	Norway	0.34	3
Nectar, Sydhavsnektar	Nora, Stabburet, Norway	Norway	0.40	3
Nectar, Tropical	Nora, Stabburet, Norway	Norway	0.44	3
Nectar, Tropical	Fellesmeieriet, Norway	Norway	0.14	3
Nectar, Tropical	Eldorado	Norway	0.17	3
Nectarines		USA	0.12	6
Olives, black Kalamata, with stone	Regina	Norway	3.25	3
Olives, black, Hojiblanca I, without stone	Mario’s, Spain	Norway	0.35	3
Olives, black, without stone	Euro Shopper, Spain	Norway	0.23	3
Olives, black, without stone	Hutesa, Spain	Norway	0.89	3
Olives, green, Hojiblanca I, without stone	Mario’s, Spain	Norway	0.99	3
Olives, green, with stone	Marmara, Turkey	Norway	2.26	3
Olives, green, without stone	Olymp, Greece	Norway	1.01	3
Olives, Kalamata, with stone	Gaea, Greece	Norway	2.11	3
Olives, Kalamata, with stone	Iliada, Greece	Norway	3.13	3
Orange	Zenta	Norway	0.83	3
Orange	Outspan, Netherlands	Norway	1.08	3
Oranges, navel		USA	0.89	6
Papaya		Norway	0.76	3

Category 10 Fruit and fruit juices continued

Product	Manufacturer / product label / country of origin	Procured in	Antioxidant content in mmol/100g	Comment
Papaya	Mali	Mali	0.36	3
Papaya, dried	Northwest Delights, USA	USA	0.14	3
Passion fruit, wild, handpicked	Malawi	Malawi	0.44	4
Peaches		USA	0.15	6
Peaches		Norway	0.10	3
Peaches, canned in heavy syrup, drained liquid	Store Brand	USA	0.11	6
Peaches, canned in heavy syrup, drained liquid	Libby	USA	0.10	6
Peaches, canned in heavy syrup, drained solids	Store Brand	USA	0.10	6
Peaches, canned in heavy syrup, drained solids	Libby	USA	0.10	6
Peaches, canned in heavy syrup, drained solids	Del Monte	USA	0.09	6
Peaches, canned with syrup	Del Monte	Norway	0.14	3
Peaches, canned with syrup	S&W Fine Food, USA	Norway	0.37	3
Peaches, canned with syrup with spices	S&W Fine Food, USA	Norway	0.17	3
Peaches, canned, in heavy syrup, drained liquid	Del Monte	USA	0.10	6
Pears	Netherlands	Norway	0.18	3
Pears, Bartlett		USA	0.22	6
Pears, Bartlett, selected halve, premium, canned	S&W Fine Food for Oluf Lorentzen A/S	Norway	0.10	3
Pears, Bosc		USA	0.18	6
Pears, composite of Bartlett, Green Anjou, and Bosc		USA	0.23	6
Pears, Green Anjou		USA	0.22	6
Pears, Red Anjou		USA	0.23	6
Physalis (Physalis peruviana), wild	Malawi	Malawi	0.37	4
Pineapple	Mali	Mali	0.29	3
Pineapple		USA	0.60	6
Pineapple, dried	Mariani, USA	USA	0.18	3
Pineapple, Gold	Del Monte	Norway	1.36	3
Pineapples, MDII, Extra Gold	Del Monte	USA	1.05	6

Category 10 Fruit and fruit juices continued

Product	Manufacturer / product label / country of origin	Procured in	Antioxidant content in mmol/100g	Comment
Plantain	Mali	Mali	0.17	3
Plums	Norway	Norway	1.02	3
Plums		USA	0.83	6
Plums, Black Diamond		USA	1.83	6
Plums, dried	Sunsweet, USA	Norway	3.24	3
Plums, red	Forlimpopoli, Italy	Norway	0.73	3
Plums, red	Kitto's	Norway	1.42	3
Plums, red, canned	S&W Fine Food for Oluf Lorentzen A/S	Norway	0.97	3
Pomegranate arils, dried	India	India	7.28	3
Pomegranate, arils		Norway	1.94	3
Pomegranate, arils		Norway	1.76	3
Pomegranate, arils and carpellar membrane		Norway	9.05	3
Pomegranate, arils and juice		Norway	2.26	3
Pomegranate, dried		Norway	5.51	
Pomegranate, freshly squeezed juice		Norway	1.59	3
Pomegranate, only the white kernels		Norway	0.88	3
Pomegranate, only yellow pith	Spain	Norway	55.52	3
Pomegranate, sour, arils and juice	Turkey	Norway	1.59	
Pomegranate, sweet, arils and juice	Spain	Norway	1.00	
Pomegranate, whole	Meypa, Turkey	Norway	2.78	3
Pomegranate, whole		Norway	1.44	3
Pomegranate, whole		Norway	5.57	3
Pomegranate, whole	Spain	Norway	6.54	3
Prune porridge	Nestlé	Norway	1.54	3
Prunes	Eldorado	Norway	3.70	3
Prunes	Sunsweet, USA	Norway	2.17	3
Prunes	Sunsweet, USA	USA	2.31	3
Prunes	Diva	Norway	1.95	3
Prunes	Angas Park, Australia	New Zealand	2.19	3
Prunes		USA	2.10	6
Raisins		USA	0.79	6
Raisins	Sun Maid, USA	USA	1.14	3
Raisins	Sunbeam Foods, Australia	New Zealand	0.79	3

Category 10 Fruit and fruit juices continued

Product	Manufacturer / product label / country of origin	Procured in	Antioxidant content in mmol/100g	Comment
Raisins	Sun Maid, USA	Norway	0.91	3
Raisins, big	Arteche, Spain	Norway	0.92	3
Raisins, green	India	India	0.65	3
Sharon		Norway	0.39	3
Sharon	Italy	Norway	0.79	3
Squash, green (Cucurbita)		Norway	0.11	3
Sweet cherries	USA	Norway	0.62	3
Sweet cherries, dark, canned	S&W Fine Food for Oluf Lorentzen A/S	Norway	1.60	3
Sweet cherries, dried	Safeway, USA	USA	4.05	3
Syrup from canned cherris		Norway	1.68	3
Syrup from canned pears		Norway	0.10	3
Syrup from canned plums		Norway	0.79	3
Tangerines		USA	0.62	6
Tangerines, Honey		USA	0.44	6
Watermelon		USA	0.18	6
Watermelon	Mali	Mali	0.02	3
Watermelon, red, seedless	Bouquet, Spania	Norway	0.06	3
Watermelon, yellow, seedless	Bouquet, Spania	Norway	0.04	3

Category 11 Grains and grain products

Product	Manufacturer / product label / country of origin	Procured in	Antioxidant content in mmol/100g	Comment
Autumn wheat, Bastian	Norwegian University of Life Sciences	Norway	3.24	5
Bagels, frozen	Hatting Bageri, Denmark	Norway	0.23	3
Bagels, plain	Store Bakery	USA	0.11	6
Bagels, plain	Pepperidge Farm	USA	0.10	6
Bagels, plain, frozen	Lenders Original Recipe	USA	0.11	6
Bagels, plain, frozen	Sara Lee	USA	0.15	6
Bagels, plain, frozen, toasted	Sara Lee	USA	0.29	6
Bagels, plain, frozen, toasted	Lenders Original Recipe	USA	0.24	6
Bagels, plain, New York style	Thomas	USA	0.12	6
Bagels, plain, New York style, toasted	Thomas	USA	0.31	6
Bagels, plain, toasted	Store Bakery	USA	0.40	6
Bagels, plain, toasted	Pepperidge Farm	USA	0.23	6
Barley, flour	Regal, Norway	Norway	0.74	3
Barley, flour	Møllerens, Norway	Norway	1.09	3
Barley, pearl barley	Regal, Norway	Norway	0.94	3
Barley, wholemeal flour, crushed	Helios, Norway	Norway	1.19	3
Biscuits, refridgerated, Big Country Buttermilk	Pillsbury	USA	0.01	6
Biscuits, refridgerated, Buttermilk Fluffy, cooked	Pillsbury Hungry Jack	USA	0.27	6
Biscuits, refrigerated, Big Country Butter Tastin' Fluffy Biscuits, cooked	Pillsbury	USA	0.28	6
Biscuits, refrigerated, Golden Layers Butter Tastin' Biscuits	Pillsbury Hungry Jack	USA	0.03	6
Biscuits, refrigerated, Golden Layers Butter Tastin', cooked	Pillsbury Hungry Jack	USA	0.19	6

Category 11 Grains and grain products continued

Product	Manufacturer / product label / country of origin	Procured in	Antioxidant content in mmol/100g	Comment
Biscuits, refrigerated, Golden Layers buttermilk	Pillsbury Hungry Jack	USA	0.08	6
Biscuits, refrigerated, Golden Layers buttermilk, cooked	Pillsbury Hungry Jack	USA	0.33	6
Biscuits, refrigerated, Grands Butter Tastin' Biscuits	Pillsbury	USA	0.08	6
Biscuits, refrigerated, Grands Butter Tastin' Biscuits, cooked	Pillsbury	USA	0.26	6
Biscuits, refrigerated, Grands Buttermilk Biscuits	Pillsbury	USA	0.02	6
Biscuits, refrigerated, Grands Buttermilk Biscuits, cooked	Pillsbury	USA	0.16	6
Bread crumbs, plain	Colonna	USA	0.32	6
Bread crumbs, plain	Store Brand	USA	0.31	6
Bread crumbs, plain	Progresso	USA	0.32	6
Bread crumbs, seasoned	Store Brand	USA	0.40	6
Bread crumbs, seasoned, garlic & herb	Progresso	USA	0.47	6
Bread crumbs, seasoned, italian style	Progresso	USA	0.42	6
Bread crumbs, seasoned, Parmesan cheese	Progresso	USA	0.37	6
Bread, Graham	Bakers, Norway	Norway	0.46	3
Bread, white	Plaza bakeri, Norway	Norway	0.20	3
Bread, white	Bakers, Norway	Norway	0.30	3
Bread, with fibre/wholemeal	Plaza bakeri, Norway	Norway	0.63	3
Bread, with fibre/wholemeal, Birkebeiner	Bakers, Norway	Norway	0.41	3
Bread, with fibre/wholemeal, Panda	Plaza bakeri, Norway	Norway	0.53	3
Bread, with fibre/wholemeal, with walnuts	Åpent bakeri, Norway	Norway	3.11	3
Buckwheat, white flour	Helios, Norway	Norway	1.73	3
Buckwheat, white flour	Nutana, Denmark	Norway	1.08	3
Buckwheat, wholemeal flour	Nutana, Denmark	Norway	2.24	3
Buckwheat, wholemeal flour	Helios, Norway	Norway	1.83	3

Category 11 Grains and grain products continued

Product	Manufacturer / product label / country of origin	Procured in	Antioxidant content in mmol/100g	Comment
Bulgur	Edel	Norway	0.20	3
Bulgur, prepared	Edel	Norway	0.04	3
Colosseo (durum)	Norwegian University of Life Sciences	Norway	2.59	5
Common millet, white flour	Mali	Mali	0.36	3
Common millet, white flour	E. Zwicky, Switzerland	Norway	0.14	3
Common millet, wholemeal flour	Mali	Mali	0.82	3
Corn Grits, white, instant	Store Brand	USA	0.12	6
Corn Grits, white, instant	Quaker	USA	0.18	6
Corn Grits, white, instant, microwave cooked	Store Brand	USA	0.02	6
Corn Grits, white, instant, microwave cooked	Quaker	USA	0.03	6
Corn Grits, white, instant, prepared with boiling water	Quaker	USA	0.05	6
Corn Grits, white, instant, prepared with boiling water	Store Brand	USA	0.03	6
Corn Grits, white, quick	Store Brand	USA	0.14	6
Corn Grits, white, quick	Quaker	USA	0.22	6
Corn Grits, white, quick, cooked on stovetop	Quaker	USA	0.06	6
Corn Grits, white, quick, cooked on stovetop	Store Brand	USA	0.04	6
Corn Grits, white, quick, microwave cooked	Store Brand	USA	0.04	6
Corn Grits, white, quick, microwave cooked	Quaker	USA	0.05	6
Corn Grits, yellow, cooked on stovetop	Quaker	USA	0.28	6
Corn Grits, yellow, quick	Store Brand	USA	0.19	6
Corn Grits, yellow, quick, cooked on stovetop	Quaker	USA	0.08	6
Corn Grits, yellow, quick, cooked on stovetop	Store Brand	USA	0.08	6
Corn Grits, yellow, quick, microwave cooked	Store Brand	USA	0.06	6
Corn Grits, yellow, quick, microwave cooked	Quaker	USA	0.08	6
Corn Meal, degermed	Name Brand	USA	0.40	6

Category 11 Grains and grain products continued

Product	Manufacturer / product label / country of origin	Procured in	Antioxidant content in mmol/100g	Comment
Corn Meal, degermed	Store Brand	USA	0.27	6
Corn tortillas, refrigerated	El Toro	USA	0.48	6
Corn tortillas, refrigerated	Guerrero	USA	0.40	6
Corn tortillas, refrigerated	Don Pancho	USA	0.38	6
Corn, whole kernel, canned, drained liquid	Lakeside Foods	USA	0.17	6
Corn, whole kernel, canned, drained solids	Lakeside Foods	USA	0.19	6
Couscous	Ferrero	Norway	0.06	3
Couscous, swelled	Ferrero	Norway	0.07	3
Cream of Wheat, cooked on stovetop, 1 minute	Nabisco	USA	0.02	6
Cream of Wheat, cooked on stovetop, 1 minute	Store Brand	USA	0.02	6
Cream of Wheat, cooked on stovetop, 2 1/2 minute	Store Brand	USA	0.03	6
Cream of Wheat, cooked on stovetop, 2 1/2 minutes	Nabisco	USA	0.03	6
Cream of Wheat, instant, microwave cooked	Store Brand	USA	0.02	6
Cream of Wheat, instant, microwave cooked	Nabisco	USA	0.02	6
Cream of Wheat, instant, not prepared	Kraft Foods North Amerika, INC	USA	0.17	3
Cream of Wheat, instant, prepared (boiled)	Kraft Foods North Amerika, INC	USA	0.05	3
Cream of Wheat, instant, prepared with boiling water	Nabisco	USA	0.01	6
Cream of Wheat, instant, prepared with boiling water	Store Brand	USA	0.04	6
Cream of Wheat, microwave cooked, 2 1/2 minute	Store Brand	USA	0.03	6
Cream of Wheat, microwave cooked, 2 1/2 minutes	Nabisco	USA	0.03	6
Crispbread, brown	Hakon, Norway	Norway	0.93	3
Crispbread, brown, rye-crisp	Ryvita, England	Norway	1.13	3
Crispbread, brown, Wasa Husmann	Wasabrød AB, Sweden	Norway	1.11	3
Crispbread, white, Frokost	Hakon, Norway	Norway	0.43	3
Crispbread, white, Wasa Frukost	Wasabrød AB, Sweden	Norway	0.43	3
Dinner rolls, brown and serve rolls	Any Brand	USA	0.07	6

Category 11 Grains and grain products continued

Product	Manufacturer / product label / country of origin	Procured in	Antioxidant content in mmol/100g	Comment
Dinner rolls, brown and serve rolls, cooked	Any Brand	USA	0.09	6
Dinner rolls, soft dinner rolls, country style	Pepperidge Farm	USA	0.05	6
Dinner rolls, soft dinner rolls, country style, cooked	Pepperidge Farm	USA	0.22	6
Dinner rolls, soft dinner rolls, Parker House	Pepperidge Farm	USA	0.07	6
Dinner rolls, soft dinner rolls, Parker House, cooked	Pepperidge Farm	USA	0.16	6
Durum wheat	Helios, Norway	Norway	0.14	3
Durum wheat, brown	Libanon	Norway	0.35	3
Durum wheat, white	Turkey	Norway	0.29	3
Egg noodles, wide	Light 'N Fluffy	USA	0.07	6
Egg noodles, wide	Mueller’s	USA	0.07	6
Egg noodles, wide, cooked	Light 'N Fluffy	USA	0.03	6
Egg noodles, wide, cooked	Mueller’s	USA	0.02	6
Egg noodles, wide, cooked	Store Brand	USA	0.04	6
Einkorn wheat, triticum monococcum	Norwegian University of Life Sciences	Norway	0.73	5
Elbow macaroni, cooked	Creamette	USA	0.03	6
Elbow macaroni, cooked	Store Brand	USA	0.03	6
English muffins, cinnamon raisin	Thomas	USA	0.20	6
English muffins, cinnamon raisin	Store Brand	USA	0.21	6
English muffins, cinnamon raisin, toasted	Store Brand	USA	0.36	6
English muffins, cinnamon raisin, toasted	Thomas	USA	0.36	6
English muffins, plain	Thomas	USA	0.04	6
English muffins, plain	Store Brand	USA	0.05	6
English muffins, plain, toasted	Store Brand	USA	0.16	6
English muffins, plain, toasted	Thomas	USA	0.11	6
Flour tortillas, refrigerated	Mex American	USA	0.06	6
Flour tortillas, refrigerated	La Favorita	USA	0.06	6
Flour tortillas, refrigerated	Foods of New Mexico	USA	0.07	6

Category 11 Grains and grain products continued

Product	Manufacturer / product label / country of origin	Procured in	Antioxidant content in mmol/100g	Comment
Fonio, whole grain	Mali	Mali	0.03	3
French bread	Any Brand	USA	0.17	6
French bread, toasted	Store Brand	USA	0.31	6
Hamburger bread, with sesame seeds	Norsk Mat, Norway	Norway	0.34	3
Hamburger/hot dog rolls, hamburger rolls	Store Brand	USA	0.18	6
Hamburger/hot dog rolls, hot dog rolls	Store Brand	USA	0.17	6
Maize cob (Corn cob)	Carmel, Israel	Norway	0.21	3
Maize cob (Corn cob)	Spain	Norway	0.26	3
Maize cob (Corn cob), frozen	Findus, Norway (corn from Hungary)	Norway	0.25	3
Maize cob (Corn cob), frozen	Enghav AS, Norway (corn from Belgium)	Norway	0.29	3
Maize cob (Corn), canned	Diva, Canada	Norway	0.26	3
Maize cob (Corn), dried	India	India	0.40	3
Maize flour, Ufa, made from locally grown white mais	Malawi	Malawi	0.32	3
Maize, white flour	Asia	Norway	0.88	3
Maize, white flour	Moka, Slovenia	Norway	0.47	3
Maize, whole grain	Mali	Mali	0.10	3
Maizena	Bestfoods Nordic A/S	Norway	0.04	3
Millet, white fluor	Jalpur, England	Norway	1.31	3
Oat bran	Møllerens, Norway	Norway	0.27	3
Oat patent flour	Regal, Norway	Norway	0.31	3
Oat, flour	Regal, Norway	Norway	0.50	3
Oat, rolled, Bjørn havregryn	Axa	Norway	0.32	3
Oat, rolled, rough oatmeal, precooked	Møllerens, Norway	Norway	0.70	3
Oatmeal porridge with milk and water, prepared		Norway	0.06	3
Oatmeal porridge with water, Bjørn Havregyn, prepared	Axa	Norway	0.06	3
Oatmeal, instant	Quaker	USA	0.46	6
Oatmeal, instant	Store Brand	USA	0.45	6
Oatmeal, instant, boiled	Quaker	USA	0.11	6
Oatmeal, instant, boiled	Store Brand	USA	0.10	6

Category 11 Grains and grain products continued

Product	Manufacturer / product label / country of origin	Procured in	Antioxidant content in mmol/100g	Comment
Oatmeal, instant, microwave cooked	Store Brand	USA	0.09	6
Oatmeal, instant, microwave cooked	Quaker	USA	0.08	6
Oatmeal, old fashioned	Store Brand	USA	0.34	6
Oatmeal, old fashioned	Quaker	USA	0.37	6
Oatmeal, old fashioned, boiled	Quaker	USA	0.11	6
Oatmeal, old fashioned, boiled	Store Brand	USA	0.10	6
Oatmeal, old fashioned, microwave cooked	Store Brand	USA	0.09	6
Oatmeal, old fashioned, microwave cooked	Quaker	USA	0.09	6
Oatmeal, quick cooking	Store Brand	USA	0.36	6
Oatmeal, quick cooking, 1-Minute Oats	Quaker	USA	0.45	6
Oatmeal, quick cooking, 1-minute oats, boiled	Quaker	USA	0.10	6
Oatmeal, quick cooking, 1-minute oats, microwave cooked	Quaker	USA	0.08	6
Oatmeal, quick cooking, boiled	Store Brand	USA	0.09	6
Oatmeal, quick cooking, microwave cooked	Store Brand	USA	0.08	6
Oats, rolled, rough oatmeal	Helios, Norway	Norway	0.54	3
Rice	Shakati Bhoug Snacks LTD Dehli	India	0.16	3
Rice porridge	Fjordland, Norway	Norway	0.06	3
Rice, brown, ecologically grown	Urtekram, Denmark	Norway	0.33	3
Rice, brown, grain, Basmati	Tilda, England	Norway	0.36	3
Rice, brown, grain, Basmati, cooked	Tilda, England	Norway	0.27	3
Rice, grain, fast	Ming	Norway	0.01	3
Rice, grain, Jasmin	Hakon, Norway	Norway	0.02	3
Rice, long grain, white	Store Brand	USA	0.08	6
Rice, long grain, white, cooked	Store Brand	USA	0.02	6
Rice, white flour	Risenta	Norway	0.06	3
Rice, white flour	Nutana, Denmark	Norway	0.05	3
Rice, white flour	Thailand	Norway	0.02	3

Category 11 Grains and grain products continued

Product	Manufacturer / product label / country of origin	Procured in	Antioxidant content in mmol/100g	Comment
Rice, white, cooked, instant	Store Brand	USA	0.02	6
Rice, white, cooked, instant	Minute	USA	0.03	6
Rice, white, extra long grain, cooked	Canilla	USA	0.03	6
Rice, white, extra long grain, cooked, (tap water)	Canilla	USA	0.03	6
Rice, white, grain, instant	Minute	USA	0.07	6
Rice, white, long grain	Canilla	USA	0.10	6
Rice, white, long grain	Store Brand	USA	0.12	6
Rice, white, long grain	Canilla	USA	0.10	6
Rice, white, long grain, american	Eldorado	Norway	0.14	3
Rice, white, long grain, cooked	Store Brand	USA	0.04	6
Rice, white, long grain, cooked	Canilla	USA	0.04	6
Rice, white, long grain, cooked (tap water)	Store Brand	USA	0.03	6
Rice, white, long grain, cooked, (tap water)	Canilla	USA	0.03	6
Rice, white, long grain, parboiled	Uncle Ben's	USA	0.17	6
Rice, white, long grain, parboiled, cooked	Uncle Ben's	USA	0.12	6
Rice, whole grain	Mali	Mali	0.02	3
Rye, flour, sieved	Regal, Norway	Norway	0.20	3
Rye, flour, sieved	Møllerens, Norway	Norway	0.25	3
Rye, squeezed, wholemeal flour	Helios, Norway	Norway	0.35	3
Rye, white flour	Regal, Norway	Norway	0.20	3
Rye, wholemeal flour	Regal, Norway	Norway	0.50	3
Rye, wholemeal, fine	Regal, Norway	Norway	0.57	3
Salmone (brohvete)	Norwegian University of Life Sciences	Norway	0.84	5
Sinskajae		Japan	0.68	5
Sorghum	Parma	Norway	0.06	3
Sorghum (Sorgam), flour	Jalpur	Norway	0.30	3
Sorghum, whole grain	Mali	Mali	0.19	3
Spaghetti, regular	Store Brand	USA	0.12	6

Category 11 Grains and grain products continued

Product	Manufacturer / product label / country of origin	Procured in	Antioxidant content in mmol/100g	Comment
Spaghetti, regular	Hakon, Norway	Norway	0.12	3
Spaghetti, regular	Buitoni, Italy	Norway	0.13	3
Spaghetti, regular	Mueller's	USA	0.12	6
Spaghetti, regular	Sopps, Norway	Norway	0.03	3
Spaghetti, regular	Ronzoni	USA	0.04	6
Spaghetti, regular, cooked	San Giorgio	USA	0.02	6
Spaghetti, regular, cooked	Mueller's	USA	0.04	6
Spaghetti, regular, cooked	Store Brand	USA	0.06	6
Spaghetti, regular, cooked	Ronzoni	USA	0.02	6
Spelt, sieved	Regal, Norway	Norway	0.21	3
Spelt, wholemeal flour, ecologically grown	Kvelde Mølle, Norway	Norway	0.21	3
Spelt, wholemeal, grov	Regal, Norway	Norway	0.16	3
Spring wheat, Bastian	Norwegian University of Life Sciences	Norway	3.31	5
Taco shells	Dinner Hakon, Norway	Norway	0.20	3
Taco shells, corn, hard, ready-to-eat	Store Brand	USA	0.53	6
Taco shells, corn, hard, ready-to-eat	Ortega	USA	0.58	6
Taco shells, corn, hard, ready-to-eat	Old El Paso	USA	0.37	6
Taco shells, Mariachi	Meadowlands, Netherlands	Norway	0.28	3
Taco shells, Texmex	Santa Maria, Danske krydderier, Denmark	Norway	0.27	3
Wheat bread	Other Brand	USA	0.32	6
Wheat bread	Store Brand	USA	0.34	6
Wheat bread	Wonder	USA	0.31	6
Wheat bread, toasted	Wonder	USA	0.54	6
Wheat bread, toasted	Store Brand	USA	0.52	6
Wheat bread, toasted	Other Brand	USA	0.60	6
Wheat germ	Møllerens, Norway	Norway	3.23	3
Wheat, white flour	Regal, Norway	Norway	0.08	3
Wheat, white flour, sieved	Møllerens, Norway	Norway	0.12	3
Wheat, white fluor, imported	Mali	Mali	0.18	3
Wheat, whole grain		India	0.38	3
Wheat, wholemeal flour		Norway	0.36	3

Category 11 Grains and grain products continued

Product	Manufacturer / product label / country of origin	Procured in	Antioxidant content in mmol/100g	Comment
Wheat, wholemeal flour, Graham flour	Helios, Norway	Norway	0.32	3
White bread, sliced	Wonder	USA	0.15	6
White bread, sliced	Store Brand	USA	0.16	6
Whole wheat bread	Oroweat	USA	0.46	6
Whole wheat bread	Nature's Own	USA	0.51	6
Whole wheat bread	Wonder	USA	0.47	6
Whole wheat bread, toasted	Wonder	USA	1.00	6
Whole wheat bread, toasted	Nature's Own	USA	0.93	6

Category 12 Herbal / traditional plant medicine

Product	Manufacturer / product label / country of origin	Procured in	Antioxidant content in mmol/100g	Comment
Amalaki (Amla), powder in capsule	The Himalaya Herbal Health Care	India	301.14	3
Angelicae Radix	Tsumura Pharmaceutical Company, Japan	Japan	2.96	5
Arjuna, powder in capsule	The Himalaya Herbal Health Care	India	146.95	3
Arnica (Arnica montana), flower and seeds, dried	Mexico	Mexico	36.28	3
Arnica (Arnica montana), leaves	Mexico	Mexico	3.72	3
Astragali Radix	Tsumura Pharmaceutical Company, Japan	Japan	4.87	5
Atractylodis Lanceae Rhizoma	Tsumura Pharmaceutical Company, Japan	Japan	7.37	5
Aurantii Nobilis Pericarpium	Tsumura Pharmaceutical Company, Japan	Japan	17.48	5
Ayur Slim, powder in capsule	The Himalaya Herbal Health Care	India	4.94	3
Blood Purifier, powder in capsule	The Himalaya Herbal Health Care	India	25.42	3
Bordelobo	Mexico	Mexico	17.98	3
Brahmi, powder in capsule	The Himalaya Herbal Health Care	India	10.40	3
Bupleuri Radix	Tsumura Pharmaceutical Company, Japan	Japan	5.66	5
Cancerina	Mexico	Mexico	19.14	3
Cascara Sagrada	Mexico	Mexico	47.15	3
Chyavanprash, Dabur	Dabur India Limited	India	35.70	3
Chyavanprash, Zandu in Asli Ghee	The Zandum Pharmaceutical Works	India	18.32	3

Category 12 Herbal / traditional plant medicine continued

Product	Manufacturer / product label / country of origin	Procured in	Antioxidant content in mmol/100g	Comment
Cimicifugae Rhizoma	Tsumura Pharmaceutical Company, Japan	Japan	64.31	5
Cinnamomi Cortex	Tsumura Pharmaceutical Company, Japan	Japan	120.18	5
Cnidii Rhizoma	Tsumura Pharmaceutical Company, Japan	Japan	6.68	5
Digestiv, powder in capsule	The Himalaya Herbal Health Care	India	7.68	3
Domiana de SanLuis	Mexico	Mexico	10.69	3
Eucalipto	Mexico	Mexico	47.30	3
Ginseng Radix	Tsumura Pharmaceutical Company, Japan	Japan	1.45	5
Glycyrrhizae Radix	Tsumura Pharmaceutical Company, Japan	Japan	11.58	5
Goshuyutou, kampo, traditional Chinese medicine from Japan, powder	Japan	Japan	132.58	3
Hochuekkito	Tsumura Pharmaceutical Company, Japan	Japan	9.67	5
Holelen	Tsumura Pharmaceutical Company, Japan	Japan	2.82	5
Huacharable	Mexico	Mexico	39.18	3
Juzentaihoto	Tsumura Pharmaceutical Company, Japan	Japan	14.18	5
Kampo, traditional Chinese medicine from Japan, powder	Japan	Japan	4.02	3
Karela, bitter gourd, powder in capsule	The Himalaya Herbal Health Care	India	7.57	3
Lasuna, garlic, powder in capsule	The Himalaya Herbal Health Care	India	0.80	3

Category 12 Herbal / traditional plant medicine continued

Product	Manufacturer / product label / country of origin	Procured in	Antioxidant content in mmol/100g	Comment
Neem Guard, powder in capsule		India	89.23	3
Nimba (Neem Tree), powder in capsule	The Himalaya Herbal Health Care	India	19.99	3
Paeoniae Radix	Tsumura Pharmaceutical Company, Japan	Japan	55.13	5
Pinelliae Tuber	Tsumura Pharmaceutical Company, Japan	Japan	0.28	5
Pinguica	Mexico	Mexico	2.31	3
Rhemanniae Radix	Tsumura Pharmaceutical Company, Japan	Japan	3.94	5
Saikokeishito	Tsumura Pharmaceutical Company, Japan	Japan	21.35	5
Sangre de grado (Croton lechleri), liquid solution	Iquitos, Peru	Peru	2897.11	3
Sano Sano	Peru	Peru	0.47	3
Scutellariae Radix	Tsumura Pharmaceutical Company, Japan	Japan	111.33	5
Shallaki, powder in capsule	The Himalaya Herbal Health Care	India	2.58	3
Shuddha guggulu, powder in capsule	The Himalaya Herbal Health Care	India	13.77	3
Stress Guard, anti stress, powder in capsule		India	6.39	3
Tagara, valerian, powder in capsule	The Himalaya Herbal Health Care	India	6.44	3
Tepezcohuite	Mexico	Mexico	64.58	3
Tetzar	Mexico	Mexico	5.88	3
Tila	Mexico	Mexico	19.49	3
Triphala, powder in capsule	The Himalaya Herbal Health Care	India	706.25	3
Hangebyakujutsutemmato	Tsumura Pharmaceutical Company, Japan	Japan	5.15	3

Category 12 Herbal / traditional plant medicine continued

Product	Manufacturer / product label / country of origin	Procured in	Antioxidant content in mmol/100g	Comment
Tulasi, (Holy basil), powder in capsule	The Himalaya Herbal Health Care	India	39.67	3
Un Compuesto, herbal condiment against insomnia	Mexico	Mexico	40.89	3
Uncaria Tomentosa (Uña de gato)	Cusco, Peru	Peru	37.10	3
Zapote	Mexico	Mexico	38.78	3
Zarzaparrilla, root	Cusco, Peru	Peru	13.73	3
Zingiberis Rhizoma	Tsumura Pharmaceutical Company, Japan	Japan	17.52	5
Zizyphi Fructus	Tsumura Pharmaceutical Company, Japan	Japan	5.88	5

Category 13 Infant foods and beverages

Product	Manufacturer / product label / country of origin	Procured in	Antioxidant content in mmol/100g	Comment
Beef	Beechnut Stage 1	USA	0.08	6
Beef	Gerber 2nd Foods	USA	0.05	6
Beef	Heinz	USA	0.06	6
Broccoli and chicken dinner	Gerber 2nd Foods	USA	0.38	6
Broccoli and chicken dinner	Gerber Graduates	USA	0.13	6
Broccoli and chicken dinner	Heinz	USA	0.45	6
Broccoli and chicken dinner, heated	Heinz	USA	0.47	6
Chicken	Heinz	USA	0.02	6
Chicken	Gerber 2nd Foods	USA	0.06	6
Chicken	Beechnut Stage 1	USA	0.10	6
Chicken and vegetable risotto, ecological (from 8 months)	Hipp, Germany	Norway	0.12	3
Chicken and vegetables (from 6 months)	Nestlé, Norway	Norway	0.15	3
Dessert, Solskinnspuré, from 6 months	Nestlé, Norway	Norway	0.86	3
Dog Rose, extract	Weiders Farmasøytiske A/S, Norway	Norway	18.52	3
Dog Rosepurée	Nestlé, Norway	Norway	6.68	3
Fruit salad, ecological (From 8 months)	Hipp, Germany	Norway	0.70	3
Green beans	Heinz	USA	0.05	6
Green beans	Gerber 1st Foods	USA	0.08	6
Human breast milk (mean of 49 samples)	Rikshospitalet University Hospital, Norway	Norway	2.03	5
Infant formula with omega 3, Collett, prepared	Nycomed Pharma	Norway	0.18	3
Infant formula, NAN, prepared	Nestlé	Norway	0.08	3
Jungle dessert	Småfolk Barnemat, Norway	Norway	0.70	3
Lamb	Beechnut Stage 1	USA	0.05	6
Lamb	Gerber 2nd Foods	USA	0.05	6
Oatmeal cereal, unprepared	Gerber	USA	0.56	6

Category 13 Infant foods and beverages continued

Product	Manufacturer / product label / country of origin	Procured in	Antioxidant content in mmol/100g	Comment
Pasta bolognese	Småfolk Barnemat, Norway	Norway	0.20	3
Pasta with beef (from 8 months)	Nestlé, Norway	Norway	0.21	3
Peaches	Beechnut Stage 1	USA	1.17	6
Peaches	Heinz	USA	1.25	6
Peaches	Gerber 1st Foods	USA	1.06	6
Peas	Gerber 1st Foods	USA	0.10	6
Peas	Heinz	USA	0.09	6
Peas	Beechnut Stage 1	USA	0.10	6
Porridge with oat and banana, prepared	Småfolk Barnemat, Norway	Norway	0.06	3
Porridge, wholemeal, with oat and apple, ecological, prepared	Hipp, Germany	Norway	0.15	3
Porridge, wholemeal, without sugar, with banana/apricot and supplements, prepared	Nestlé, Norway	Norway	0.27	3
Porridge, wholemeal, without sugar, with fruit and supplements, prepared	Nestlé, Norway	Norway	0.31	3
Prune juice	Nestlé, Norway	Norway	1.18	3
Spaghetti bolognese, ecological	Hipp, Germany	Norway	0.14	3
Squash	Gerber 1st Foods	USA	0.11	6
Squash	Heinz	USA	0.17	6
Stew with chicken, turkey and herbs	Småfolk Barnemat, Norway	Norway	0.19	3
Turkey	Heinz	USA	0.04	6
Turkey	Gerber 2nd Foods	USA	0.05	6
Turkey	Beechnut Stage 1	USA	0.06	6
Turkey, sticks and diced	Beechnut Table Time	USA	0.05	6
Turkey, sticks and diced	Gerber Graduates	USA	0.06	6
Veal	Beechnut Stage 1	USA	0.07	6
Veal	Gerber 2nd Foods	USA	0.05	6
Vegetable and beef dinner	Heinz	USA	0.09	6
Vegetable and beef dinner	Gerber 2nd Foods	USA	0.12	6
Vegetable and beef dinner	Beechnut Stage 2	USA	0.06	6

Category 14 Legumes

Product	Manufacturer / product label / country of origin	Procured in	Antioxidant content in mmol/100g	Comment
Beans, red, canned, boiled	Conservas Viter, Spain	Norway	0.33	3
Beans, white, large size, haricotes lingots Blanc	Toko-sun, Netherlands	Norway	0.36	3
Berlotti beans	McKenzie’s	New Zealand	1.96	3
Black eye beans, haricot blance	Thailand	Norway	0.75	3
Black eye beans, white, cornille	GFT Darmstadt, Germany	Norway	0.47	3
Black eyes beans, white, medium size, dry	India	India	0.50	3
Blue beans		Norway	0.85	3
Blue beans, cooked		Norway	0.98	3
Broad beans, green		Norway	1.97	3
Broad beans, split	Toko-sun, Netherlands	Norway	1.64	3
Chickpeas	India	India	0.19	3
Chickpeas	Kkorhan, Turkey	Norway	0.22	3
Chickpeas, small size	India	India	0.57	3
Chinese broad bean paste		Japan	0.85	3
Edamame, frozen		USA	0.47	6
Edamame, frozen, prepared		USA	0.43	6
Flageolets beans, green, canned, boiled	Roland, Belgium	Norway	0.20	3
Kidney beans, canned, light red	Store Brand	USA	0.27	6
Kidney beans, canned, light red, cooked	Store Brand	USA	0.26	6
Kidney beans, dry	India	India	0.81	3
Kidney beans, large size	India	India	0.81	3
Kidney beans, medium size, dry	India	India	1.39	3
Kidney beans, striped, large size, dry	India	India	1.61	3
Lentils, black with peel	India	India	0.66	3
Lentils, Cole-Dal, yellow, split	India	India	0.16	3

Category 14 Legumes continued

Product	Manufacturer / product label / country of origin	Procured in	Antioxidant content in mmol/100g	Comment
Lentils, green	Turkey	Norway	1.00	3
Lentils, Masoor-Dal Sabat, dark brown, with peel	India	India	0.34	3
Lentils, Masoor-Dal, pink, without peel	India	India	0.10	3
Lentils, Moong-Dal, yellow, split	India	India	0.15	3
Lentils, Posune	India	India	1.14	3
Lentils, red	Kkorhan, Turkey	Norway	0.23	3
Lentils, Toor Dal, yellow	India	India	0.35	3
Lentils, white, split, Maa-Dal	India	India	0.16	3
Moung Dal, with peel	India	India	0.53	3
Mung beans	Toko-sun, Netherlands	Norway	0.34	3
Mung beans, peeled	Thailand	Norway	0.10	3
Navy beans	Viter	Norway	0.38	3
Navy beans, dry		USA	0.57	6
Navy beans, dry, cooked		USA	0.11	6
Peas	Produced in Norway for Hakon Gruppen A/S	Norway	0.26	3
Peas	Norrek Engros A/S	Norway	0.25	3
Peas, baby, canned, drained liquid	Store Brand	USA	0.10	6
Peas, baby, canned, drained liquid	Le Sueur	USA	0.11	6
Peas, baby, canned, drained solids	Store Brand	USA	0.01	6
Peas, regular, canned, drained liquid	Del Monte	USA	0.15	6
Peas, regular, canned, drained liquid	Store Brand	USA	0.06	6
Peas, regular, canned, drained liquid	Green Giant	USA	0.17	6
Peas, regular, canned, drained solids	Store Brand	USA	0.09	6
Peas, regular, canned, drained solids	Green Giant	USA	0.12	6
Peas, regular, canned, drained solids	Del Monte	USA	0.12	6
Peas, regular, canned, drained solids, heated	Store Brand	USA	0.11	6

Category 14 Legumes continued

Product	Manufacturer / product label / country of origin	Procured in	Antioxidant content in mmol/100g	Comment
Peas, regular, canned, drained solids, heated	Green Giant	USA	0.12	6
Peas, regular, canned, drained solids, heated	Del Monte	USA	0.11	6
Peas, yellow, split	S&W, USA	Norway	0.12	3
Pinto beans	S&W, USA	Norway	1.15	3
Pinto beans, dried		USA	1.18	6
Pinto beans, dried, cooked		USA	0.19	6
Soy beans, Red Bean (Soja rouge)	Thailand	Norway	0.82	3
Soy beans, white, small size, dry	India	India	0.99	3
Soy milk with chocolate, Alpro Soya	Alpro	Norway	0.30	3
Soy milk, chocolate, enriched with vitamins A, D & calsium	Trader Joe's, USA	USA	0.25	3
Soy milk, ecological	Provamel Alpro NV, Belgium	Norway	0.08	3
Soy milk, original, enriched with vitamins A, D & calsium	Trader Joe's, USA	USA	0.05	3
Soy milk, Soy dream original	The Hain Celestial Group, Inc	Norway	0.07	3
Soy milk, vanilla, enriched with vitamins A, D & calsium	Trader Joe's, USA	USA	0.10	3
Soyatein (protein rich soya)	Vital Soya Industy, Sri Goindwal Sahib, District Amritsar	India	1.32	3
Tofu naturell	Scandinavian Soya	Norway	0.09	3
Tofutti, creamy smooth	Tofutti London	Norway	0.05	3
Urid Dal Split	India	Norway	0.36	3

Category 15 Meat and meat products

Product	Manufacturer / product label / country of origin	Procured in	Antioxidant content in mmol/100g	Comment
Bacon, fried	Spis, Norway	Norway	0.85	3
Bacon, Frokostbacon	Spis, Norway	Norway	0.32	3
Beef hot dogs	Kahn's	USA	0.43	6
Beef hot dogs	Oscar Mayer	USA	0.47	6
Beef hot dogs	Ball Park	USA	0.37	6
Beef hot dogs	Store Brand	USA	0.39	6
Beef hot dogs	Nathan's	USA	0.38	6
Beef hot dogs	Farmer John	USA	0.51	6
Beef hot dogs, cooked (boiled)	Ball Park	USA	0.34	6
Beef hot dogs, cooked (boiled)	Oscar Mayer	USA	0.47	6
Hamburger frozen	Produced for Dagens by Danish Prima, Denmark	Norway	0.04	3
Hamburger, frozen	Spis, Norway	Norway	0.12	3
Hamburger, frozen, fried	Norsk Mat, Norway	Norway	0.11	3
Hot dog, barbecue	Gilde, Norway	Norway	0.51	3
Hot dog, barbecue, fried	Gilde, Norway	Norway	0.59	3
Hot dog, frankfurter	Spis, Norway	Norway	0.29	3
Liver, ox, frozen		Norway	0.71	3
Liver, pork, frozen		Norway	0.46	3
Meat franks	Oscar Mayer	USA	0.30	6
Meat franks	Bryan	USA	0.32	6
Meat franks	Hygrade	USA	0.16	6
Meat franks, cooked (boiled)	Hygrade	USA	0.24	6
Meat franks, cooked (boiled)	Oscar Mayer	USA	0.35	6
Meat franks, jumbo	Bar S, Jumbo	USA	0.25	6
Salami	Gilde, Norway	Norway	0.46	3
Salami, Jubel	Grilstad fabrikker AS, Norway	Norway	0.08	3
Steak, calf		Norway	0.02	3
Steak, moose		Norway	0.03	3
Steak, ox		Norway	0.01	3
Steak, pork		Norway	0.00	3
Steak, reindeer		Norway	0.01	3

Category 16 Miscellanous ingredients

Product	Manufacturer / product label / country of origin	Procured in	Antioxidant content in mmol/100g	Comment
Artificial sweetener	Equal	USA	0.02	6
Artificial sweetener, calorie-free	Splenda	USA	0.00	6
Artificial sweetener; calorie-free	Sugar Twin	USA	0.01	6
Aspartame	Monsanto	USA	0.04	6
Barley malt syrup, organic	Sweet Cloud	USA	2.12	6
Brown rice malt syrup, organic	Sweet Cloud	USA	0.72	6
Brown rice syrup, powder, organic	Emperor`s Kitchen	USA	1.04	6
Coffee mate, powder	Nestlé	Norway	0.11	3
Corn syrup, light	Karo	USA	0.01	6
Corn syrup, light	Clements Foods Co	USA	0.00	6
Honey	Store Brand	USA	0.16	6
Honey	Sue Bee	USA	0.14	6
Honey	Golden Blossom	USA	0.19	6
Honey	Dutch Gold	USA	0.14	6
Honey	Any Brand	USA	0.13	6
Honey, clover	FMV	USA	0.16	6
Honey, pure	Best-in, England	Norway	0.16	3
Malt-extract, Moss	Jensen & Co, Norway	Norway	1.69	3
Maple syrup, 100% pure	Spring Tree	USA	0.45	6
Molasses, dark	Brer Raddit	USA	4.90	6
Salt, iodized	Morton	USA	0.00	6
Soumbala	Mali	Mali	1.70	3
Stevia Dryss, powder	www.naturlegemiddel.no	Norway	0.31	
Stevia Dråper, liquid	www.naturlegemiddel.no	Norway	0.40	
Stevia Plus, powder plus fiber	Sweet Leaf	Norway	0.10	
Stevia powder, pluss fiber	Sweet Leaf	USA	0.04	6
Stevia, Max 80, liquid	Stevia Canada, JG Group	Canada	0.02	
Sugar colour, Negro	Toro, Norway	Norway	15.54	3

Category 16 Miscellanous ingredients continued

Product	Manufacturer / product label / country of origin	Procured in	Antioxidant content in mmol/100g	Comment
Sugar, raw cane, organic	Sweet Cloud	USA	0.33	6
Sugar, refined, granulated	Kroger	USA	0.00	6
Sugar, turbinado	Sugar In The Raw	USA	0.21	6
Vinegar, apple cider	Other Brand	USA	0.11	6
Vinegar, apple cider	Heinz	USA	0.11	6
Vinegar, apple cider	Heinz	Norway	0.10	3
Vinegar, apple cider	Store Brand	USA	0.12	6
Vinegar, balsamic	Brand 2	USA	0.74	6
Vinegar, balsamic	Brand 3	USA	0.88	6
Vinegar, balsamic	Brand 1	USA	0.24	6
Vinegar, destilled white	Store Brand	USA	0.00	6
Vinegar, destilled white	Heinz	USA	0.00	6
Vinegar, distilled, white	Other brand	USA	0.00	6
Vinegar, red wine	Other Brand	USA	0.28	6
Vinegar, red wine	Store Brand	USA	0.23	6
Vinegar, red wine	Regina	USA	0.41	6

Category 17 Mixed food entrees

Product	Manufacturer / product label / country of origin	Procured in	Antioxidant content in mmol/100g	Comment
Bacon egg and cheese biscuit	McDonald's	USA	0.10	6
Baked beans, pork and beans in brown sugar sauce	Bush's	USA	0.24	6
Baked beans, pork and beans in brown sugar sauce	Heinz	USA	0.29	6
Baked beans, pork and beans in brown sugar sauce	Campbell's	USA	0.22	6
Baked beans, pork and beans in brown sugar sauce	B & M	USA	0.39	6
Baked beans, pork and beans in brown sugar sauce	Store Brand	USA	0.29	6
Baked beans, pork and beans in brown sugar sauce, heated	Bush's	USA	0.21	6
Baked beans, pork and beans in brown sugar sauce, heated	Store Brand	USA	0.28	6
Baked beans, pork and beans in brown sugar sauce, heated	B & M	USA	0.38	6
Baked beans, pork and beans in tomato sauce	Bush's	USA	0.19	6
Baked beans, pork and beans in tomato sauce	Campbell's	USA	0.21	6
Baked beans, pork and beans in tomato sauce	B & M	USA	0.16	6
Baked beans, pork and beans in tomato sauce	Store Brand	USA	0.14	6
Baked beans, pork and beans in tomato sauce	Heinz	USA	0.18	6
Baked beans, pork and beans in tomato sauce, heated	Campbell's	USA	0.21	6
Baked beans, pork and beans in tomato sauce, heated	Store Brand	USA	0.17	6
Baked beans, vegetarian	B & M	USA	0.29	6

Category 17 Mixed food entrees continued

Product	Manufacturer / product label / country of origin	Procured in	Antioxidant content in mmol/100g	Comment
Baked beans, vegetarian	Store Brand	USA	0.23	6
Baked beans, vegetarian	Bush's	USA	0.25	6
Baked beans, vegetarian	Campbell`s	USA	0.22	6
Baked beans, vegetarian	Heinz	USA	0.26	6
Baked beans, vegetarian, heated	Bush's	USA	0.27	6
Baked beans, vegetarian, heated	Heinz	USA	0.24	6
Bean and cheese burritos, frozen	Las Campanas	USA	0.13	6
Bean and cheese burritos, frozen	Old El Paso	USA	0.08	6
Bean and cheese burritos, frozen	Tina's	USA	0.11	6
Bean and cheese burritos, frozen	Marquez	USA	0.13	6
Bean and cheese burritos, frozen, cooked	Tina's	USA	0.13	6
Bean and cheese burritos, frozen, cooked	Old El Paso	USA	0.09	6
Beef and bean burritos, frozen	Tina's	USA	0.09	6
Beef and bean burritos, frozen	Patio	USA	0.07	6
Beef and bean burritos, frozen	Marquez	USA	0.12	6
Beef and bean burritos, frozen	El Monterey	USA	0.17	6
Beef and bean burritos, frozen, cooked	Tina's	USA	0.11	6
Beef and bean burritos, frozen, cooked	Patio	USA	0.11	6
Beef and bean burritos, frozen, family pack	Tina's	USA	0.17	6
Beef stew, canned	Store Brand	USA	0.14	6
Beef stew, canned	Armour	USA	0.07	6
Beef stew, canned	Castleberry	USA	0.18	6
Beef stew, canned	Dinty Moore	USA	0.12	6
Beef stew, canned	Austex	USA	0.14	6
Beef stew, canned, cooked	Dinty Moore	USA	0.13	6
Big Mac, national	McDonald's	USA	0.08	6
Big Mac, no cheese, national	McDonald's	USA	0.09	6
BK Big Fish with cheese	Burger King	USA	0.09	6

Category 17 Mixed food entrees continued

Product	Manufacturer / product label / country of origin	Procured in	Antioxidant content in mmol/100g	Comment
BK Broiler	Burger King	USA	0.11	6
Breast Filet	Wendy's	USA	0.12	6
Burrito, bean	Taco Bell	USA	0.16	6
Burrito, supreme with beef	Taco Bell	USA	0.12	6
Burrito, supreme with chicken	Taco Bell	USA	0.13	6
Burrito, supreme with steak	Taco Bell	USA	0.12	6
Cheese lasagna, frozen, five cheese	Stouffer's	USA	0.73	6
Cheese lasagna, frozen, five cheese, cooked	Stouffer's	USA	0.53	6
Cheese lasagna, frozen, Mozzarella	Budget Gourmet	USA	0.14	6
Cheese lasagna, frozen, three cheese	Budget Gourmet	USA	0.23	6
Cheese lasagna, frozen, three cheese, cooked	Budget Gourmet	USA	0.18	6
Cheese pizza, frozen, regular thin crust	Tony's	USA	0.13	6
Cheese pizza, frozen, rising crust	Tombstone	USA	0.16	6
Cheeseburger	Burger King	USA	0.15	6
Cheeseburger, national	McDonald's	USA	0.11	6
Chicken pot pie, frozen, cooked	Banquet	USA	0.05	6
Chicken Sandwich	Burger King	USA	0.17	6
Chili with meat and beans, canned	Hormel	USA	0.52	6
Chili with meat and beans, canned	Chili Man	USA	0.41	6
Chili with meat and beans, canned	Wolf	USA	0.40	6
Chili with meat and beans, canned	Bryan	USA	0.42	6
Chili with meat and beans, canned	Dennison's	USA	0.42	6
Chili with meat and beans, canned	Nalley	USA	0.32	6
Chili with meat and beans, canned	Armour	USA	0.40	6
Chili with meat and beans, canned (hot)	Store Brand	USA	0.39	6

Category 17 Mixed food entrees continued

Product	Manufacturer / product label / country of origin	Procured in	Antioxidant content in mmol/100g	Comment
Chili with meat and beans, canned (thick)	Nalley	USA	0.48	6
Chili with meat and beans, canned, heated	Hormel	USA	0.50	6
Chili with meat, no beans, canned	Hormel	USA	0.39	6
Chili with meat, no beans, canned	Armour	USA	0.45	6
Chili with meat, no beans, canned	Bunker Hill	USA	0.46	6
Chili with meat, no beans, canned	Store Brand	USA	0.35	6
Chili with meat, no beans, canned	Wolf	USA	0.49	6
Chili with meat, no beans, canned, heated	Hormel	USA	0.40	6
Classic Double with Cheese	Wendy's	USA	0.07	6
Croissanwich with egg and cheese	Burger King	USA	0.15	6
Croissanwich with sausage and cheese	Burger King	USA	0.21	6
Croissanwich with sausage, egg and cheese	Burger King	USA	0.16	6
Double Whopper	Burger King	USA	0.07	6
Double Whopper with cheese	Burger King	USA	0.04	6
Egg McMuffin	McDonald's	USA	0.09	6
Eggwich with bacon and cheese	Burger King	USA	0.06	6
Eggwich with bacon, egg and cheese	Burger King	USA	0.08	6
Eggwich with egg and cheese	Burger King	USA	0.06	6
Filet-o-Fish	McDonald's	USA	0.13	6
Ham Egg and Cheese Bagel	McDonald's	USA	0.13	6
Hamburger	McDonald's	USA	0.14	6
Hamburger	Burger King	USA	0.18	6
Hamburger with cheese, 1/4 lb Single	Wendy's	USA	0.12	6
Hamburger with cheese, Junior	Wendy's	USA	0.10	6
Hamburger, Junior	Wendy's	USA	0.14	6

Category 17 Mixed food entrees continued

Product	Manufacturer / product label / country of origin	Procured in	Antioxidant content in mmol/100g	Comment
Hamburger, No Cheese, 1/4 lb Single	Wendy's	USA	0.12	6
Hotcakes and sausage	McDonald's	USA	0.18	6
Lasagna with meat, frozen, lower fat	Weight Watchers Smart Ones	USA	0.13	6
Lasagna with meat, frozen, lower fat	Lean Cuisine	USA	0.37	6
Lasagna with meat, frozen, regular, cooked	Stouffer's	USA	0.18	6
Lasagna with meat, frozen, regular, cooked	Michelina's	USA	0.12	6
Lasagna with meat, regular, frozen	Stouffer's	USA	0.48	6
Lasagna with meat, regular, frozen	Michelina's	USA	0.16	6
Macaroni and cheese (microwaveable cans), canned	Hormel Kid's Kitchen	USA	0.12	6
Macaroni and cheese mix (with dry cheese powder)	Kraft	USA	0.06	6
Macaroni and cheese mix (with dry cheese powder)	Store Brand	USA	0.06	6
Macaroni and cheese mix (with dry cheese powder), prepared	Kraft	USA	0.04	6
Macaroni and cheese mix (with prepared cheese sauce), prepared	Kraft Velveeta	USA	0.03	6
Macaroni and cheese mix (with prepared cheese sauce), prepared	Kraft Deluxe	USA	0.03	6
Macaroni and cheese mix (with prepared cheese sauce), prepared	Store Brand	USA	0.05	6
Macaroni and cheese, canned	Chef Boyardee	USA	0.04	6
Macaroni and cheese, canned	Franco American	USA	0.04	6
Nachos	Taco Bell	USA	0.36	6
Nachos supreme	Taco Bell	USA	0.30	6
Pizza, Big One Classic, prepared	Stabburet, Norway	Norway	0.13	3

Category 17 Mixed food entrees continued

Product	Manufacturer / product label / country of origin	Procured in	Antioxidant content in mmol/100g	Comment
Pizza, cheese pizza, frozen, regular thin crust	Celeste pizza for one	USA	0.15	6
Pizza, cheese pizza, frozen, regular thin crust	Mr. P's	USA	0.16	6
Pizza, cheese pizza, frozen, regular thin crust	Totino's	USA	0.10	6
Pizza, cheese pizza, frozen, regular thin crust	McCain Ellio's	USA	0.11	6
Pizza, cheese pizza, frozen, rising crust	Tony's	USA	0.08	6
Pizza, cheese pizza, frozen, rising crust	DiGiorno	USA	0.21	6
Pizza, cheese pizza, frozen, rising crust	Red Baron	USA	0.19	6
Pizza, cheese, classic hand-tossed	Domino's	USA	0.20	6
Pizza, cheese, large deep dish	Little Caesar’s	USA	0.18	6
Pizza, cheese, original crust	Papa John's	USA	0.30	6
Pizza, cheese, regular crust	Pizza Hut	USA	0.18	6
Pizza, cheese, thick crust	Pizza Hut	USA	0.17	6
Pizza, cheese, thin crust	Papa John's	USA	0.26	6
Pizza, cheese, thin crust	Little Caesar’s	USA	0.22	6
Pizza, cheese, thin crust	Pizza Hut	USA	0.20	6
Pizza, cheese, ultimate deep dish	Domino's	USA	0.20	6
Pizza, extravaganza feast, classic hand-tossed	Domino's	USA	0.18	6
Pizza, Grandiosa	Stabburet, Norway	Norway	0.31	3
Pizza, Grandiosa, prepared	Stabburet, Norway	Norway	0.25	3
Pizza, La Mia Pizzeria Speciale, ham and tomato, prepared	Stabburet, Norway	Norway	0.10	3
Pizza, Original Round, cheese	Little Caesar’s	USA	0.21	6
Pizza, Original Round, meal+ vegetab	Little Caesar’s	USA	0.19	6
Pizza, pepperoni, classic hand-tossed	Domino's	USA	0.21	6
Pizza, pepperoni, large deep dish	Little Caesar’s	USA	0.16	6
Pizza, pepperoni, original crust	Papa John's	USA	0.24	6

Category 17 Mixed food entrees continued

Product	Manufacturer / product label / country of origin	Procured in	Antioxidant content in mmol/100g	Comment
Pizza, pepperoni, original round	Little Caesar’s	USA	0.17	6
Pizza, pepperoni, regular crust, prepared	Pizza Hut	USA	0.18	6
Pizza, pepperoni, thick crust	Pizza Hut	USA	0.14	6
Pizza, pepperoni, ultimate deep dish	Domino's	USA	0.19	6
Pizza, Pig's Knuckle, prepared	Peppes Pizza, Norway	Norway	0.24	3
Pizza, Pizza Margherita	Comisal Int.Srl.Italy for Hakon	Norway	0.31	3
Pizza, Pizza Margherita, prepared	Comisal Int.Srl.Italy for Hakon	Norway	0.33	3
Pizza, prepared	First Price	Norway	0.08	3
Pizza, prepared	Eldorado	Norway	0.15	3
Pizza, Ristorante Pizza Speciale, prepared	Dr Oetker	Norway	0.19	3
Pizza, sausage & pepperoni pizza, frozen, cooked	Red Baron	USA	0.18	6
Pizza, Super Suprime, regular crust	Pizza Hut	USA	0.20	6
Pizza, The Works, original crust	Papa John's	USA	0.23	6
Pizza, thin crust	Domino's	USA	0.28	6
Quarter pounder, national	McDonald's	USA	0.08	6
Quarter Pounder, no cheese, national	McDonald's	USA	0.09	6
Sausage & pepperoni pizza, frozen, cooked	Tony's	USA	0.16	6
Sausage biscuit	McDonald's	USA	0.37	6
Sausage biscuit with egg	McDonald's	USA	0.21	6
Sausage breakfast burrito	McDonald's	USA	0.09	6
Sausage McMuffin	McDonald's	USA	0.23	6
Sausage McMuffin with egg	McDonald's	USA	0.16	6
Spaghetti with meat, canned	Chef Boyardee	USA	0.22	6
Spaghetti with meat, canned, heated	Chef Boyardee	USA	0.22	6
Spaghetti with meat, Spaghettios with Meatballs, canned	Franco American	USA	0.17	6
Spaghetti with meat, Spaghettios with Meatballs, canned, heated	Franco American	USA	0.16	6

Category 17 Mixed food entrees continued

Product	Manufacturer / product label / country of origin	Procured in	Antioxidant content in mmol/100g	Comment
Spaghetti, Garfield Spaghettios, no meat, canned	Franco American	USA	0.13	6
Spaghetti, no meat, canned	Franco American	USA	0.12	6
Spaghetti, Spaghettios, no meat, canned	Franco American	USA	0.14	6
Spaghetti, Spaghettios, no meat, canned, heated	Franco American	USA	0.13	6
Spaghetti, Where's Waldo, no meat, canned	Franco American	USA	0.14	6
Spanish Omelet Bagel	McDonald's	USA	0.15	6
Steak egg and cheese bagel	McDonald's	USA	0.08	6
Stew, in brown sauce	Trondhjems, Norway	Norway	0.19	3
Stew, light type	Terina as, Norway	Norway	0.13	3
Taco salad	Taco Bell	USA	0.13	6
Taco, crunchy	Taco Bell	USA	0.24	6
Taco, soft with beef	Taco Bell	USA	0.10	6
Taco, soft with chicken	Taco Bell	USA	0.06	6
Taco, soft with steak	Taco Bell	USA	0.07	6
Turkey pot pie, frozen, cooked	Swanson	USA	0.06	6
Vegatarian burgers, Morningstar Farms Garden Veggie	Morningstar Farms	USA	0.30	6
Vegetarian burger, Morningstar Farms Harvest Burger's	Morningstar Farms	USA	0.17	3
Vegetarian burger, Morningstar Farms Harvest Burger's, fried	Morningstar Farms	USA	0.19	3
Vegetarian burgers, Boca Burgers Chef Max's All American Classic	Boca Burgers	USA	0.11	6
Vegetarian burgers, Morningstar Farms Grillers Hamburger Style	Morningstar Farms	USA	0.20	6
Veggie burgers	Gardenburger Original	USA	0.17	6
Whopper	Burger King	USA	0.06	6
Whopper with cheese	Burger King	USA	0.06	6

Category 18 Nuts and seeds

Product	Manufacturer / product label / country of origin	Procured in	Antioxidant content in mmol/100g	Comment
Almonds, with pellicle	Kjøkkensjefens	Norway	0.23	1, 3
Almonds, with pellicle	Eldorado	Norway	0.37	1, 3
Almonds, with pellicle	Coop Chef's	Norway	0.28	1, 3
Almonds, with pellicle		USA	0.53	1, 6
Almonds, with pellicle, sliced	Blue Diamond, USA	Norway	0.26	1, 3
Almonds, without pellicle (scalded using hot water)	Eldorado	Norway	0.13	1, 3
Almonds, without pellicle (scalded using hot water)	Coop Chef's	Norway	0.22	1, 3
Almonds, without pellicle, sliced	Freia	Norway	0.20	1, 3
Brazil nuts		USA	0.47	1, 6
Brazil nuts, with pellicle (partly)	Den Lille Nøttefabrikken	Norway	0.50	1, 3
Cashews, without pellicle		USA	0.66	1, 6
Cashews, without pellicle, roasted	Den Lille Nøttefabrikken, Norway	Norway	0.40	1, 3
Chest nuts, with pellicle (purchased with shell)		Italy	4.67	3
Chest nuts, without pellicle (purchased with shell)		Italy	0.75	3
Flaxseed	Peru	Peru	0.64	4
Flaxseed, ground		USA	1.13	6
Flaxseed, whole brown		USA	0.80	6
Hazelnuts, roasted with salt and spices, with pellicle	Iran	Iran	0.46	3
Hazelnuts, with pellicle	Den Lille Nøttefabrikken, Norway	Norway	0.49	3
Hazelnuts, with pellicle		Norway	0.50	3
Hazelnuts, with pellicle	Sunport	Norway	0.69	3
Hazelnuts, with pellicle		USA	0.94	6
Hazelnuts, without pellicle	Sunrise Food	Norway	0.08	
Hazelnuts, without pellicle	Sunport	Norway	0.16	3

Category 18 Nuts and seeds continued

Product	Manufacturer / product label / country of origin	Procured in	Antioxidant content in mmol/100g	Comment
Kernel from watermelon, roasted with salt and spices	Iran	Iran	3.27	3
Kernels from pumpkin, roasted with salt and spices	Iran	Iran	0.40	3
Macadamia nuts, without pellicle	Den Lille Nøttefabrikken, Norway	Norway	0.55	1, 3
Macadamia nuts, without pellicle		USA	0.44	1, 6
Peanut butter, coarse type	Mills, Norway	Norway	0.47	1, 3
Peanut butter, creamy	Skippy	USA	0.66	1, 6
Peanut butter, creamy	Jif	USA	0.57	1, 6
Peanut butter, crunchy	Store Brand	USA	0.51	1, 6
Peanut butter, crunchy	Skippy	USA	0.55	1, 6
Peanuts, Malawi nuts, Traditional African Roasted Peanuts	Rab Processors Ltd, Malawi	Malawi	0.89	1, 3
Peanuts, Polly, roasted, with salt, without pellicle	KiMs, Norway	Norway	0.62	1, 3
Peanuts, roasted, with pellicle (purchased with shell)	Food Man	Norway	1.97	1 ,3
Peanuts, without pellicle		USA	0.35	1, 6
Pecans, with pellicle	Den Lille Nøttefabrikken, Norway	Norway	8.24	3
Pecans, with pellicle	San Lázara	Mexico	10.62	3
Pecans, with pellicle	Sunport	Norway	7.31	3
Pecans, with pellicle	The Green Valley	Norway	9.24	3
Pecans, with pellicle		Norway	6.32	3
Pecans, with pellicle	La Pasiega, Mexico	Mexico	7.91	3
Pecans, with pellicle		USA	9.67	6
Pine nuts	Den Lille Nøttefabrikken, Norway	Norway	0.52	1, 3
Pine nuts		USA	0.71	1, 6
Pine nuts	Davy's, Holland	Norway	0.10	3
Pine nuts, ecologically grown	Urtekram, Denmark	Norway	0.07	3
Pistachios	India	Norway	4.98	3
Pistachios	Den Lille Nøttefabrikken, Norway	Norway	0.78	3

Category 18 Nuts and seeds continued

Product	Manufacturer / product label / country of origin	Procured in	Antioxidant content in mmol/100g	Comment
Pistachios	Sunport	Norway	1.08	3
Pistachios	Iran	Iran	1.16	3
Pistachios		Norway	1.00	3
Pistachios		USA	1.43	6
Pistachios (purchased with shell)	Mexico	Mexico	1.41	3
Pistachios, roasted with salt and spices	Iran	Iran	1.38	3
Poppy seeds	Spice Cargo	Mexico	0.44	3
Poppy seeds		USA	0.03	6
Poppy seeds, dried	Black Boy, Rieber og søn	Norway	0.31	3
Poppy seeds, dried	TRS Wholesale CO, England	Norway	0.16	3
Sesame seeds	Nutana, Denmark	Norway	1.21	3
Sesame seeds	Spice Cargo	Mexico	0.95	3
Sesame seeds (ajonjoli)	La Surtidora	Mexico	1.32	3
Sesame seeds, black	India	India	0.26	3
Sesame seeds, hulled		USA	0.06	6
Sesame seeds, white	India	India	0.49	3
Sesame seeds, with shell	Risenta, Finland	Norway	1.16	3
Sesame seeds, without shell	Hakon, Norway	Norway	0.30	3
Sesame seeds, without shell	NatuVit, Denmark	Norway	0.43	3
Sunflower seeds	Hakon, Norway	Norway	7.50	3
Sunflower seeds	NatuVit, Denmark	Norway	5.39	3
Walnuts, with pellicle	USA	Norway	15.16	3
Walnuts, with pellicle	Den Lille Nøttefabrikken, Norway	Norway	14.29	3
Walnuts, with pellicle		Norway	25.41	3
Walnuts, with pellicle	Diamond	Norway	16.02	3
Walnuts, with pellicle		USA	13.13	6
Walnuts, with pellicle	India	India	15.84	3
Walnuts, with pellicle	Demanter, Bio'noix, France	Norway	19.75	3
Walnuts, with pellicle (purchased with shell and cupule)		Italy	18.67	3
Walnuts, with pellicle (purchased with shell)	Shells Bl	Norway	33.09	3
Walnuts, with pellicle (purchased with shell)		Italy	33.04	3

Category 18 Nuts and seeds continued

Product	Manufacturer / product label / country of origin	Procured in	Antioxidant content in mmol/100g	Comment
Walnuts, with pellicle (purchased with shell)	India	India	15.76	3
Walnuts, with pellicle (purchased with shell)	Natural	Norway	31.38	3
Walnuts, with pellicle (purchased with shell)		Norway	33.29	3
Walnuts, without pellicle		Norway	1.81	3
Walnuts, without pellicle (purchased with shell and cupule)		Italy	0.46	3
Walnuts, without pellicle (purchased with shell)	Shells	Norway	0.74	3
Walnuts, without pellicle (purchased with shell)		Italy	1.04	3
Walnuts, without pellicle (purchased with shell)		Norway	0.79	3
Walnuts, without pellicle (purchased with shell)	Natural	Norway	1.27	3

Category 19 Poultry and poultry products

Product	Manufacturer / product label / country of origin	Procured in	Antioxidant content in mmol/100g	Comment
BBQ chicken wings, frozen	Tyson	USA	0.39	6
BBQ chicken wings, frozen	TGIF	USA	0.07	6
BBQ chicken wings, frozen	Simmons	USA	0.21	6
BBQ chicken wings, frozen, cooked in conventional oven	Tyson	USA	0.38	6
BBQ chicken wings, frozen, cooked in conventional oven	Mixed brands	USA	0.33	6
BBQ chicken wings, frozen, cooked in conventional oven	Remarkable	USA	0.12	6
BBQ chicken wings, frozen, cooked in conventional oven	Simmons	USA	0.23	6
BBQ chicken wings, frozen, cooked in conventional oven	TGIF	USA	0.08	6
BBQ chicken wings, frozen, microwave cooked	Tyson	USA	0.34	6
BBQ chicken wings, frozen, microwave cooked	TGIF	USA	0.08	6
BBQ chicken wings, frozen, microwave cooked	Simmons	USA	0.28	6
Chicken hot dogs	Gwaltney	USA	0.15	6
Chicken hot dogs	Shorgood	USA	0.20	6
Chicken hot dogs	Weaver	USA	0.15	6
Chicken hot dogs	Talmadge Farms	USA	0.18	6
Chicken hot dogs	Grillmaster	USA	0.16	6
Chicken hot dogs, cooked	Gwaltney	USA	0.17	6
Chicken liver, frozen	Prior, Norway	Norway	1.00	3
Chicken liver, frozen, fried	Prior, Norway	Norway	0.96	3
Chicken McGrill, national	McDonald's	USA	0.07	6
Chicken McNuggets	McDonald's	USA	0.20	6
Chicken Nuggets	Wendy's	USA	0.25	6
Chicken nuggets, frozen	Weaver	USA	0.12	6
Chicken nuggets, frozen	Store Brand	USA	0.09	6
Chicken nuggets, frozen, cooked	Store Brand	USA	0.14	6
Chicken nuggets, frozen, cooked	Advance Fast Fixin'	USA	0.12	6
Chicken nuggets, frozen, cooked	Weaver	USA	0.16	6

Category 19 Poultry and poultry products continued

Product	Manufacturer / product label / country of origin	Procured in	Antioxidant content in mmol/100g	Comment
Chicken patties, frozen	Kings Delight	USA	0.13	6
Chicken patties, frozen	Tyson	USA	0.13	6
Chicken patties, frozen, cooked	Tyson	USA	0.15	6
Chicken Sandwich, grilled	Wendy's	USA	0.15	6
Chicken tenders	Burger King	USA	0.12	6
Chicken tenders, frozen	Mixed brands	USA	0.13	6
Chicken tenders, frozen	Tyson	USA	0.09	6
Chicken tenders, frozen, cooked in conventional oven	Store Brand	USA	0.16	6
Chicken tenders, frozen, cooked in conventional oven	Tyson	USA	0.12	6
Chicken tenders, frozen, cooked in conventional oven	Banquet	USA	0.12	6
Chicken tenders, frozen, cooked in conventional oven	Weaver	USA	0.11	6
Chicken tenders, frozen, cooked in conventional oven	Butterball	USA	0.12	6
Chicken tenders, frozen, microwave cooked	Mixed brands	USA	0.14	6
Chicken tenders, frozen, southern, cooked in conventional oven	Banquet	USA	0.15	6
Chicken, drumstick, grilled, with skin	Prior, Norway	Norway	0.44	3
Chicken, drumstick, grilled, without skin	Prior, Norway	Norway	0.65	3
Chicken, with skin, frozen	Prior, Norway	Norway	0.06	3
Chicken, with skin, frozen, fried	Prior, Norway	Norway	0.05	3
Chicken, without skin, frozen	Prior, Norway	Norway	0.05	3
Chicken, without skin, frozen, fried	Prior, Norway	Norway	0.06	3
Crispy chicken sandwich, national	McDonald's	USA	0.18	6
Turkey hot dog, Frankfurter	Prior, Norway	Norway	0.61	3
Turkey hot dogs (barbecue)	Prior, Norway	Norway	0.76	3

Category 20 Snacks

Product	Manufacturer / product label / country of origin	Procured in	Antioxidant content in mmol/100g	Comment
Biscuits, Bixit	Sætre AS, Norway	Norway	0.66	3
Biscuits, crunch cream croustillants	StMichael for Marks and Spencer	Norway	0.36	3
Biscuits, oat, Kornmo	Sætre AS, Norway	Norway	0.86	3
Biscuits, RITZ crackers	Oxford Biscuits A/S, Denmark	Norway	0.41	3
Cheese crackers with cheese filling	Austin	USA	0.77	1, 6
Cheese crackers with cheese filling	Frito Lay	USA	0.73	1, 6
Cheese crackers with cheese filling	Lance	USA	0.82	1, 6
Cheese crackers with cheese filling	Store Brand	USA	0.76	1, 6
Cheese crackers with peanut butter filling	Little Debbie	USA	0.95	1, 6
Cheese crackers with peanut butter filling	Austin	USA	0.90	1, 6
Cheese crackers with peanut butter filling	Lance	USA	0.86	1, 6
Cheese puff-type cheese snacks	Cheetos	USA	0.50	1, 6
Cheese puff-type cheese snacks, crunchy	Cheetos	USA	0.63	1, 6
Kernel popcorn, air popped	Orville Redenbacher	USA	0.83	6
Kernel popcorn, air popped	Jolly Time	USA	0.86	6
Kernel popcorn, air popped	Store Brand	USA	0.82	6
Kernel popcorn, oil popped	Jolly Time	USA	0.75	6
Kernel popcorn, oil popped	Store Brand	USA	0.74	6
Kernel popcorn, oil popped	Orville Redenbacher	USA	0.72	6
Multigrain snack chips	Sun Chips	USA	0.75	1, 6
Popcorn	Maarud, Norway	Norway	0.56	3
Popcorn, microwave, 94% fat free butter flavor	Pop Secret	USA	0.46	6

Category 20 Snacks continued

Product	Manufacturer / product label / country of origin	Procured in	Antioxidant content in mmol/100g	Comment
Popcorn, microwave, 94% fat free butter flavor	Orville Redenbacher Smart Pop	USA	0.59	6
Popcorn, microwave, 94% fat free, butter flavor	Store Brand	USA	0.59	6
Popcorn, microwave, 94% fat free, butter flavor	Jolly Time Healthy Pop	USA	0.62	6
Popcorn, microwave, butter flavor	Store Brand	USA	0.47	6
Popcorn, microwave, butter flavor	Pop Secret	USA	0.48	6
Popcorn, microwave, butter flavor	Orville Redenbacher	USA	0.37	6
Popcorn, microwave, butter flavor	Jolly Time Butterlicious	USA	0.52	6
Potato chips	Lays Classic	USA	0.78	1, 6
Potato chips	Lays	USA	0.74	1, 6
Potato chips	Store Brand	USA	0.47	1, 6
Potato chips	Ruffles	USA	0.59	1, 6
Potato chips, fat free, made with olean (olestra), original flavor	Pringles	USA	0.44	1, 6
Potato chips, made with olestra, original flavor	Lays Wow	USA	0.15	1, 6
Potato chips, Potetgull, classic, salt	Maarud, Norway	Norway	0.66	1, 3
Pretzels	Store Brand	USA	0.77	6
Pretzels	Frito Lay Rold Gold	USA	1.10	6
Pretzels	Snyders of Hanover/Utz	USA	0.98	6
Saltine crackers	Store Brand	USA	0.28	6
Saltine crackers	Nabisco Premium Original	USA	0.37	6
Saltine crackers	Sunshine Krispy Original	USA	0.43	6
Saltine crackers	Sunshine Keebler Zesty Original	USA	0.28	6
Toasty peanut butter filled crackers	Keebler	USA	1.06	1, 6
Toasty peanut butter filled crackers	Golden Flake	USA	0.97	1, 6

Category 20 Snacks continued

Product	Manufacturer / product label / country of origin	Procured in	Antioxidant content in mmol/100g	Comment
Toasty peanut butter filled crackers	Ritz Bits	USA	1.07	1, 6
Toasty peanut butter filled crackers	Austin	USA	0.97	1, 6
Toasty peanut butter filled crackers	Little Debbie	USA	1.17	1, 6
Tortilla chips	Mission	USA	0.77	1, 6
Tortilla chips, 100% white corn	Tostitos	USA	0.77	1, 6
Tortilla chips, cheese	Maarud, Norway	Norway	0.48	1, 3
Tortilla chips, cooler ranch flavor	Doritos	USA	0.74	1, 6
Tortilla chips, made with olestra, nacho cheesier tortilla flavor	Doritos Wow	USA	0.22	1, 6
Tortilla chips, made with olestra, restaurant style	Tostitos Wow	USA	0.16	1, 6
Tortilla chips, nacho cheesier flavor	Doritos	USA	0.72	1, 6

Category 21 Soups, sauces, dressings and salsa

Product	Manufacturer / product label / country of origin	Procured in	Antioxidant content in mmol/100g	Comment
Barbecue sauce	Heinz, Netherlands	Norway	0.55	3
Barbeque ketchup	Idun, Norway	Norway	0.36	3
Barbeque oil, alround	Santa Maria, Sweden	Norway	0.81	3
Barbeque oil, alround	Black Boy, Rieber og søn	Norway	0.97	3
Barbeque sauce	Heinz	Norway	0.53	3
BBQ classic	HP	Norway	0.80	3
BBQ sauce orginal	Santa Maria	Norway	0.37	3
Bruschetta	Le ricette di MONTANINI, Italy	Norway	0.82	3
Chicken broth, 99% fat free	Swanson	USA	0.00	6
Concentrato di pomedoro	La BioIdea	Norway	0.89	3
Doppio concentrato di pomodoro	Mutti	Norway	1.18	3
Dressing, Caesar, salad dressing, max 3% fat	Idun, Norway	Norway	0.08	1, 3
Dressing, French ,salad dressing, fat free	Kraft Free	USA	0.04	1, 6
Dressing, French, salad dressing	Idun, Norway	Norway	0.02	1, 3
Dressing, French, salad dressing, lite	Western	USA	0.42	1, 6
Dressing, French, salad dressing, regular	Store Brand	USA	0.44	1, 6
Dressing, French, salad dressing, regular	Kraft Creamy	USA	0.46	1, 6
Dressing, French, salad dressing, regular	Western	USA	0.44	1, 6
Dressing, French, salad dressing, regular	Henri's	USA	0.42	1, 6
Dressing, Italian, salad dressing, fat free	Seven Seas	USA	0.08	1, 6
Dressing, Italian, salad dressing, fat free	Kraft Free	USA	0.08	1, 6
Dressing, Italian, salad dressing, lite	Wishbone	USA	0.14	1, 6

Category 21 Soups, sauces, dressings and salsa continued

Product	Manufacturer / product label / country of origin	Procured in	Antioxidant content in mmol/100g	Comment
Dressing, Italian, salad dressing, regular	Wishbone	USA	0.32	1, 6
Dressing, Italian, salad dressing, regular	Store Brand	USA	0.41	1, 6
Dressing, Ranch, salad dressing	Kraft	USA	0.46	1, 6
Dressing, Ranch, salad dressing	Wishbone	USA	0.41	1, 6
Dressing, Ranch, salad dressing	Hidden Valley	USA	0.37	1, 6
Dressing, Ranch, salad dressing, fat free	Kraft	USA	0.02	1, 6
Dressing, Ranch, salad dressing, fat free	Hidden Valley	USA	0.22	1, 6
Dressing, Ranch, salad dressing, fat free	Wishbone	USA	0.03	1, 6
Dressing, Ranch, salad dressing, light	Wishbone	USA	0.18	1, 6
Dressing, Ranch, salad dressing, light	Kraft	USA	0.14	1, 6
Dressing, Ranch, salad dressing, light	Hidden Valley	USA	0.19	1, 6
Dressing, Soltørket tomat (Sundried Tomato), salad dressing	Idun, Norway	Norway	0.65	1, 3
Dressing, Thousand Island, salad dressing	Idun, Norway	Norway	0.11	1, 3
Dressing, Thousand Island, salad dressing, fat free	Kraft	USA	0.07	1, 6
Dressing, Thousand Island, salad dressing, lite	Kraft	USA	0.32	1, 6
Gravy, beef, Savory	Heinz, Home Style	USA	0.06	6
Gravy, turkey, canned	Franco American	USA	0.06	6
Gravy, turkey, canned	Store Brand	USA	0.04	6
Gravy, turkey, canned, Home Style	Heinz	USA	0.05	6
Hot chili sauce	Heinz	Norway	0.36	3
Jalfrezi cooking sauce	Santa Maria	Norway	0.59	3
Mashed potatoe, powder	Mills, Norway	Norway	1.20	3
Mashed potatoes, prepared	Maggi	Norway	0.23	3
Mashed potatoes, prepared	Mills, Norway	Norway	0.28	3

Category 21 Soups, sauces, dressings and salsa continued

Product	Manufacturer / product label / country of origin	Procured in	Antioxidant content in mmol/100g	Comment
Mashed potatoes, with milk powder, prepared	Maggi	Norway	0.17	3
Mashed potatoes, with milk powder, prepared	Mills, Norway	Norway	0.22	3
Mayonnaise, original	Mills, Norway	Norway	1.08	1, 3
Meditarranean olive sauce	Heinz	Norway	0.28	3
Mexican sauce	Heinz	Norway	0.36	3
Mustard, Bodsennep, prepared	Stabburet, Norway	Norway	1.70	3
Mustard, Dijon originale, prepared	Maille, France	Norway	2.48	3
Mustard, hot, prepared	Stabburet, Norway	Norway	1.76	3
Mustard, Original, prepared	Slotts, Sweden	Norway	2.09	3
Mustard, paste, prepared	Japan	Japan	2.61	3
Mustard, Premium Americana, prepared	S&W, USA	Norway	1.43	3
Mustard, Savora, prepared	Colman's	Norway	0.80	3
Mustard, yellow, Classic Yellow Mustard, prepared	French`s	USA	1.57	6
Mustard, yellow, prepared	Store Brand	USA	1.52	6
Mustard, yellow, prepared	Other Brand	USA	1.42	6
Nali Ginger Chilie Sauce	Nali, Malawi	Malawi	0.70	3
Nali Hot Peri-Peri Sauce	Nali, Malawi	Malawi	1.42	3
Passata di pomodoro con basilico	La BioIdea	Norway	0.36	3
Paste, canned tomato	Contadina	USA	1.03	6
Paste, canned tomato	Hunt's	USA	0.98	6
Paste, canned tomato	Store Brand	USA	0.88	6
Pelali mediterranei	Mutti	Norway	0.22	3
Pesto Rosso	Le ricette di MONTANINI, Italy	Norway	0.91	3
Pesto Rosso Piccante	HGL Gourmet Line's middelhavskjøkken	Norway	0.49	3
Pesto Rosso Piccante, sundried tomato	HGL Gourmet Line's middelhavskjøkken	Norway	1.20	3
Pesto Rosso, sundried tomato	Paradiso, Italy	Norway	0.68	3
Pesto Rosso, sundried tomato	Le ricette di MONTANINI, Italy	Norway	0.86	3
Pesto, alla Genovese con Basilico fresco	Barilla, Italy	Norway	2.55	3

Category 21 Soups, sauces, dressings and salsa continued

Product	Manufacturer / product label / country of origin	Procured in	Antioxidant content in mmol/100g	Comment
Pesto, basil	Le ricette di MONTANINI, Italy	Norway	2.48	3
Pesto, basil, Italia	ICA, Sweden	Norway	4.36	3
Piccante sauce, medium	Pace	Norway	0.37	3
Pizza filling	Mesterkokkens, Norway	Norway	0.48	3
Pizza filling, with edible mushroom (Agaricus bisporus) and bell pepper, Casa di Italia, prepared	Toro, Norway	Norway	0.71	3
Pizza filling, with tomatoes and herbs, Casa di Italia, prepared	Toro, Norway	Norway	0.89	3
Pizza filling, with tomatoes and onion, Casa di Italia, prepared	Toro, Norway	Norway	0.62	3
Polpa pomodori in finissimini pezzi	Mutti	Norway	0.34	3
Polpapezzi di pomodori italiani	Mutti	Norway	0.38	3
Pomodori capperi acciughi	Azienda Terra Shardana	Norway	0.47	3
Pomodori del sole	Cicina Antica	Norway	0.82	3
Pomodori secchi	Le ricette di MONTANINI, Italy	Norway	0.72	3
Pomodorini di collini	Mutti	Norway	0.49	3
Pomodoro al basilico	Andalini	Norway	0.56	3
Pomodoro mousse	Azienda Terra Shardana	Norway	0.80	3
Ragu alla bolognese	Barilla, Italy	Norway	0.37	3
Ricotta	Barilla, Italy	Norway	0.41	3
Salsa dip, medium	Old El Paso	Norway	0.51	3
Salsa dip, medium	First Price	Norway	0.31	3
Salsa, All natural mild, bottled	Tostitos	USA	0.33	6
Salsa, Chunky salsa, medium	Santa Maria	Norway	0.34	3
Salsa, Chunky salsa, medium	ICA	Norway	3.92	3
Salsa, Garlic salsa	Old El Paso	Norway	0.43	3
Salsa, garlic, TEXMEX, medium	Santa Maria, Sweden	Norway	0.44	3
Salsa, Habanero and tequila salsa, extra hot	Santa Maria	Norway	0.27	3

Category 21 Soups, sauces, dressings and salsa continued

Product	Manufacturer / product label / country of origin	Procured in	Antioxidant content in mmol/100g	Comment
Salsa, Kutbil-ik de Chile Habanero	Mexico	Mexico	1.59	3
Salsa, Rio grande, medium	Santa Maria	Norway	0.28	3
Salsa, Roasted garlic, bottled	Tostitos	USA	0.31	6
Salsa, Salsa picante verde de Chile Habanero	Mexico	Mexico	0.89	3
Salsa, Sweet red chili salsa, medium	Santa Maria	Norway	0.46	3
Salsa, Thick & Chunky mild, bottled	Pace	USA	0.36	6
Salsa, Thick 'n' Chunky mild, bottled	Old El Paso	USA	0.31	6
Salsa, Thick 'n Chunky, medium, bottled	Old El Paso	USA	0.30	6
Salsa, Thick 'n Chunky, medium, bottled	Old El Paso	Norway	0.49	3
Salsa, Tropical salsa, medium	Santa Maria, Sweden	Norway	0.29	3
Sauce for pasta, tomato, oregano, onion and garlic	Knorr	Norway	0.63	3
Sauce for pasta, with sundried tomato and shallot, Casa di Italia	Toro, Norway	Norway	0.34	3
Sauce, BBQ	HP Foods, England	Norway	0.75	3
Sauce, Bearnaise, prepared	Toro, Norway	Norway	0.03	3
Sauce, brown, for meatballs, prepared	Knorr	Norway	0.09	3
Sauce, canned tomato	Hunt's	USA	0.34	6
Sauce, canned tomato	Store Brand	USA	0.29	6
Sauce, Cayenne pepper, Red Devil	Trappeyey's Foods Inc.	Norway	0.26	3
Sauce, chili, Oriental	Uncle Ben's	Norway	0.36	3
Sauce, chilli sauce, hot	Heinz, Portugal	Norway	0.53	3
Sauce, chilli, hot	Blue Dragon, Malaysia	Norway	0.20	3
Sauce, for pasta	First Price	Norway	0.31	3
Sauce, for pasta with parmesan and creem, Al forno	Dolmio	Norway	0.20	3
Sauce, for pasta, classic	Knorr	Norway	0.55	3
Sauce, for pasta, creamed, Casa d`Italia	Toro, Norway	Norway	0.24	3

Category 21 Soups, sauces, dressings and salsa continued

Product	Manufacturer / product label / country of origin	Procured in	Antioxidant content in mmol/100g	Comment
Sauce, for pasta, no meat, chunky garden combination	Ragu	USA	0.37	6
Sauce, for pasta, no meat, mushroom flavor	Hunt's	USA	0.51	6
Sauce, for pasta, no meat, mushroom flavor	Prego	USA	0.51	6
Sauce, for pasta, no meat, old world style	Ragu	USA	0.40	6
Sauce, for pasta, no meat, three-cheese flavor	Prego	USA	0.37	6
Sauce, for pasta, spanish olives	Meridian	Norway	0.61	3
Sauce, for pasta, tomato and mushrooms	Meridian	Norway	0.49	3
Sauce, for pasta, with basil	Barilla, Italy	Norway	0.57	3
Sauce, for pasta, with basil, heated	Barilla, Italy	Norway	0.54	3
Sauce, for pasta, with chilli	ICA	Norway	0.35	3
Sauce, for pasta, with chilli and onion	Knorr	Norway	0.39	3
Sauce, for pasta, with chilli and onion, heated	Knorr	Norway	0.57	3
Sauce, for pasta, with chilli, Arrabbiata	Barilla, Italy	Norway	0.39	3
Sauce, for pasta, with extra garlic	Dolmio	Norway	0.61	3
Sauce, for pasta, with extra garlic, heated	Dolmio	Norway	0.68	3
Sauce, for pasta, with extra spices	Dolmio	Norway	0.50	3
Sauce, for pasta, with extra vegetables	Dolmio	Norway	0.43	3
Sauce, for pasta, with garlic	ICA	Norway	0.50	3
Sauce, for pasta, with garlic	Eldorado	Norway	0.33	3
Sauce, for pasta, with sundried tomatoes	Dolmio	Norway	0.79	3
Sauce, for pizza	Idun, Norway	Norway	0.63	3
Sauce, for pizza, Casa di Italia	Toro, Norway	Norway	0.77	3
Sauce, for pizza, Orginal	Dolmio	Norway	0.38	3
Sauce, for pizza, Spicy	Mutti	Norway	0.57	3

Category 21 Soups, sauces, dressings and salsa continued

Product	Manufacturer / product label / country of origin	Procured in	Antioxidant content in mmol/100g	Comment
Sauce, for spagetti, Italian tomato sauce with spices	Knorr	Norway	0.31	3
Sauce, for spaghetti	Sopps, Norway	Norway	0.27	3
Sauce, for spaghetti	Toro, Norway	Norway	0.26	3
Sauce, Hollandaise, prepared	Knorr	Norway	0.01	3
Sauce, Indian Korma	Uncle Ben's	Norway	0.21	3
Sauce, Indian Tandori	Toro, Norway	Norway	0.52	3
Sauce, orginal	HP	Norway	1.48	3
Sauce, slices of tomato, with basil, garlic and oregano	SW	Norway	0.18	3
Sauce, slices of tomato, with chilli and mexican spices, canned	SW	Norway	0.26	3
Sauce, slices of tomato, with garlic, oregano and basil, canned	SW	Norway	0.31	3
Sauce, slices of tomato, with pepper, onion and creole spices, canned	SW	Norway	0.27	3
Sauce, soya sauce	Kikkoman Foods, Netherlands	Norway	2.68	3
Sauce, soya sauce, dark	Amoy Food LTD, Hong Kong	Norway	0.68	3
Sauce, soya sauce, light	Amoy Food LTD, Hong Kong	Norway	0.54	3
Sauce, sun dried tomato	Heinz	Norway	0.52	3
Sauce, taco, hot	Landlord	Norway	4.25	3
Sauce, taco, medium	ICA	Norway	0.88	3
Sauce, taco, medium	Santa Maria	Norway	0.36	3
Sauce, taco, medium	First Price	Norway	4.66	3
Sauce, taco, medium	Casa Fiesta	Norway	3.50	3
Sauce, taco, medium spiced, TexMex	Santa Maria, Sweden	Norway	0.20	3
Sauce, taco, mildly spiced	ICA, Belgium	Norway	3.17	3
Sauce, taco, smooth, medium spiced	Old El Paso	Norway	0.28	3
Sauce, taco, smooth, strong spiced	Old El Paso	Norway	0.18	3
Sauce, taco, strong spiced	ICA, Belgium	Norway	3.50	3
Sauce, taco, strong spiced, TexMex	Santa Maria, Sweden	Norway	0.22	3

Category 21 Soups, sauces, dressings and salsa continued

Product	Manufacturer / product label / country of origin	Procured in	Antioxidant content in mmol/100g	Comment
Sauce, taco, TexMex, hot	Santa Maria, Sweden	Norway	0.48	3
Sauce, taco, TexMex, mild	Santa Maria, Sweden	Norway	0.44	3
Sauce, Tikka Masala, Indian	Uncle Ben's	Norway	0.36	3
Sauce, tomato, Tomatensauce, Toskana	Zwergwnwiese	Norway	0.42	3
Sauce, tomato, with basalmicovinegar, basil and oil, canned	Hunt's	Norway	0.25	3
Sauce, tomato, with mushroom, canned	Hunt's	Norway	0.24	3
Sauce, with chili	HP Foods, England	Norway	0.24	3
Sauce, wrap, medium spiced	Casa Fiesta	Norway	3.70	3
Soup stok, japanese (bonito,tangl), powder		Japan	0.93	3
Soup stok, japanese (small dried sardine), powder	Japan	Japan	0.07	3
Soup, chicken noodle cup a soup, dry	Knorr	USA	0.45	6
Soup, chicken noodle, canned, ready-to-eat	Progresso Traditional	USA	0.02	6
Soup, chicken noodle, canned, ready-to-eat, Kitchen Classics	Campbell's	USA	0.02	6
Soup, chinese chicken soup stock, powder	Japan	Japan	0.41	3
Soup, clam chowder condensed	Campbell`s	USA	0.05	3
Soup, condensed chicken noodle soup	Campbell's	USA	0.03	6
Soup, condensed chicken noodle soup	Store Brand	USA	0.01	6
Soup, condensed cream of chicken soup	Store Brand	USA	0.03	6
Soup, condensed cream of chicken soup	Campbell's	USA	0.04	6
Soup, condensed cream of mushroom soup	Campbell's	USA	0.04	6
Soup, condensed tomato	Store Brand	USA	0.66	6
Soup, curry chicken, cup a soup	Knorr	Denmark	0.14	5

Category 21 Soups, sauces, dressings and salsa continued

Product	Manufacturer / product label / country of origin	Procured in	Antioxidant content in mmol/100g	Comment
Soup, Minestrone	Campbell`s	Norway	0.25	3
Soup, Pea soup, Svensk/Swedish type, prepared	Toro, Norway	Norway	0.52	3
Soup, Pea soup, yellow, prepared	Toro, Norway	Norway	0.63	3
Soup, potato and leek, cup a soup	Knorr	Denmark	0.07	5
Soup, Ramen noodle soup, beef flavor, dry	Nissin Top Ramen Noodles	USA	0.41	6
Soup, tomato, ABC kremet tomatsuppe, prepared	Toro, Norway	Norway	0.14	3
Soup, tomato, canned, prepared	Campbell`s	Norway	0.29	3
Soup, tomato, canned, prepared	Heinz	Norway	0.24	3
Soup, tomato, condensed	Campbell's	USA	0.24	6
Soup, tomato, cup a soup, prepared	Knorr	Denmark	0.18	5
Soup, tomato, prepared	Confecta, Norway	Norway	0.07	3
Soup, tomato, prepared	Knorr	Norway	0.16	3
Soup, tomato, prepared	Toro, Norway	Norway	0.15	3
Soup, tomato, Rett i koppen, prepared	Toro, Norway	Norway	0.17	3
Soup, tomato, Sunny tomato extra, prepared	Knorr	Norway	0.34	3
Soup, tomato, with macaroni, prepared	First Price	Norway	0.13	3
Soup, tomato, with mozzarella and herbs, Rett i Koppen, prepared	Toro, Norway	Norway	0.12	3
Soup, tomtato, Meksikansk tomatsuppe fra guerroro, World menus, prepared	Toro, Norway	Norway	0.16	3
Spaghetti sauce, prepared	Toro, Norway	Norway	0.31	3
Sundried tomato paste	Sacla, Italy	Norway	0.75	3
Sundried tomatoes, in oil	Eldorado	Norway	1.22	3
Sundried tomatoes, in oil, chopped	Paradisi	Norway	1.69	3
Tabasco Brand	Mcllhenny Company	Norway	0.56	3

Category 21 Soups, sauces, dressings and salsa continued

Product	Manufacturer / product label / country of origin	Procured in	Antioxidant content in mmol/100g	Comment
Tiptree organic tomato chutney	Wilkin&Son Ltd, England	Norway	0.35	3
Tomato ketchup	Idun, Norway	Norway	0.37	3
Tomato ketchup	Heinz	Norway	0.33	3
Tomato ketchup	Heinz	USA	0.41	6
Tomato ketchup	First Price	Norway	0.19	3
Tomato ketchup	Store Brand	USA	0.37	6
Tomato ketchup	Mutti	Norway	0.43	3
Tomato ketchup	Pølsemaker Bergby	Norway	0.26	3
Tomato ketchup	Eldorado	Norway	0.77	3
Tomato ketchup, classico	La BioIdea	Norway	0.53	3
Tomato ketchup, organic	Heinz	Norway	0.24	3
Tomato ketchup, orginal	Gourmet Compagniet	Norway	0.14	3
Tomato ketchup, Red Hot Chili	Idun, Norway	Norway	0.25	3
Tomato purée	Diva, Turkey	Norway	1.25	3
Tomato purée	ICA	Norway	1.25	3
Tomato purée	Starlands	Norway	1.19	3
Tomato purée	Landlord	Norway	0.53	3
Tomato purée	Eldorado	Norway	1.27	3
Tomato purée	Heinz	Norway	0.96	3
Tomato purée, canned	Pope	USA	0.42	6
Tomato purée, canned	Tuttoroso	USA	0.43	6
Tomato purée, canned	Progresso	USA	0.49	6
Tomato purée, canned	Contadina	USA	0.40	6
Tomato purée, canned	Redpack	USA	0.42	6
Tomato purée, organic	Biona	Norway	0.97	3
Tomato sauce	Toro, Norway	Norway	0.20	3
Tomatoes, with chili, chopped, canned	Eldorado	Norway	0.25	3
Tomatoes, with garlic, chopped, canned	Eldorado	Norway	0.38	3
Tomatoes, with sweet basil and oregano, chopped, canned	Eldorado	Norway	0.45	3
Vinaigrette with sundried tomato	Crown	Norway	0.41	3
Wok sauce, Indian Curry	Toro, Norway	Norway	0.46	3
Wok sauce, Red Hot Curry Thailand	Santa Maria, Sweden	Norway	0.41	3

Category 22 Spices and herbs

Product	Manufacturer / product label / country of origin	Procured in	Antioxidant content in mmol/100g	Comment
A condiment with red pepper and six other spices, dried ground	Japan	Japan	6.08	3
Ajwain fruit pods, whole		Iran	0.94	3
Ajwain fruit pods, dried	India	India	28.42	3
Allspice, dried ground	Hindu, Norway	Norway	99.28	3
Allspice, dried ground	Black Boy, Rieber og søn	Norway	101.52	3
Alpine lady's-mantle, leaves, dried	The Norwegian Crop Research Institute, Norway	Norway	130.36	5
Angelica, fresh	The Norwegian Crop Research Institute, Norway	Norway	0.66	5
Angelica, leaves, dried	Norsk Øko-Urt AB, Norway	Norway	25.25	5
Angelica, seeds, dried	Norsk Øko-Urt AB, Norway	Norway	8.66	5
Anisisop, leaves, dried	Norsk Øko-Urt AB, Norway	Norway	33.14	5
Ash, young leaves, dried	The Norwegian Crop Research Institute, Norway	Norway	47.78	5
Bacon and eggs, flower, dried	The Norwegian Crop Research Institute, Norway	Norway	56.10	5
Barbeque spicemix	Santa Maria, Sweden	Norway	1.65	3
Barbeque spicemix	Engebretsen AS, Norway	Norway	2.13	3
Barberry, bark	The Norwegian Crop Research Institute, Norway	Norway	55.63	5
Basil, dried	Gökqehan ,Turkey	Norway	9.86	3
Basil, dried	Norsk Øko-Urt AB, Norway	Norway	28.10	5
Basil, dried		USA	12.31	6

Category 22 Spices and herbs continued

Product	Manufacturer / product label / country of origin	Procured in	Antioxidant content in mmol/100g	Comment
Basil, dried	Black Boy, Rieber og søn	Norway	30.86	3
Basil, dried	Spice Cargo	Mexico	18.24	3
Basil, dried	Natures Treats Australia PTY LTD, Australia	New Zealand	0.49	3
Basil, fresh	Norway	Norway	1.12	3
Basil, fresh		Norway	0.67	3
Basil, fresh		USA	0.82	6
Bay leaves, dried	Santa Maria	Norway	31.29	3
Bay leaves, dried	Black Boy, Rieber og søn	Norway	24.29	3
Bay leaves, fresh	Natures Treats Australia PTY LTD, Australia	New Zealand	15.05	3
Bearberry (Arctostaphylos uva-ursi), leaves, dried	The Norwegian Crop Research Institute, Norway	Norway	182.10	5
Bee balm (Monarda didyma), flower, dried	Norsk Øko-Urt AB, Norway	Norway	46.56	5
Betonica officinalis, dried	The Norwegian Crop Research Institute, Norway	Norway	9.41	5
Betterave	Mali	Mali	2.34	3
Birch, leaves, dried	Norsk Øko-Urt AB, Norway	Norway	30.44	5
Birch, leaves, fresh	Norway	Norway	26.23	4
Birdcherry, flower, dried	The Norwegian Crop Research Institute, Norway	Norway	23.08	5
Biting stonecrop, dried	The Norwegian Crop Research Institute, Norway	Norway	11.89	5
Blackberry, leaves, dried	The Norwegian Crop Research Institute, Norway	Norway	23.31	5
Blackcurrant, leaves, dried	Norsk Øko-Urt AB, Norway	Norway	97.83	5
Calamus root (Acorus calamus), rhizome	The Norwegian Crop Research Institute, Norway	Norway	6.65	5
Caper, flower	Turkey	Norway	1.00	3

Category 22 Spices and herbs continued

Product	Manufacturer / product label / country of origin	Procured in	Antioxidant content in mmol/100g	Comment
Caper, fruits and stem, Caprons Finos	Cervera, Denmark	Norway	0.84	3
Caper, small	Turkey	Norway	0.94	3
Car Magaz, whole kernels	Asian Bazaar	Mexico	0.77	3
Caraway seeds, dried	Norsk Øko-Urt AB, Norway	Norway	3.35	5
Caraway seeds, dried	Black Boy, Rieber og søn	Norway	4.48	3
Cardamom pod, green, whole	India	India	1.85	3
Cardamom seeds (from green pod)	Roopaks, Ajmal Khan, N. Dehli	India	1.64	3
Cardamom seeds, dried	India	India	1.13	3
Cardamom seeds, dried	Black Boy, Rieber og søn	Norway	0.48	3
Cardamom, dried ground	Engebretsen AS, Norway	Norway	2.35	3
Cardamom, dried ground	Santa Maria, Sweden	Norway	1.65	3
Cardamom, whole fruit, dried	India	India	1.64	3
Cayenne pepper, dried ground	Spice Cargo	Mexico	5.38	3
Cayenne pepper, dried ground	Santa Maria	Norway	4.18	3
Cayenne pepper, dried ground	Black Boy, Rieber og søn	Norway	5.90	3
Celery seeds, whole	Spice Cargo	Mexico	8.17	3
Celery, leaves, dried	Norsk Øko-Urt AB, Norway	Norway	16.91	5
Chervil, dried	The Norwegian Crop Research Institute, Norway	Norway	17.67	5
Chili, Chile Ancho, dark, whole, dried	Mexico	Mexico	5.09	3
Chili, Chile de Arcbol, small red, whole, dried	Mexico	Mexico	3.11	3
Chili, Chile Don Piquin, with seeds, crushed, dried	Mexico	Mexico	4.20	3
Chili, Chile Guajillo, dark, whole, dried	Mexico	Mexico	2.25	3
Chili, Chile Pasilla, dark, whole, dried	Verde Valle, Mexico	Mexico	7.54	3

Category 22 Spices and herbs continued

Product	Manufacturer / product label / country of origin	Procured in	Antioxidant content in mmol/100g	Comment
Chili, Chile Pasilla, dark, whole, dried	La Merced, Mexico	Mexico	3.01	3
Chili, Chile Piquin, dried ground		Mexico	1.40	3
Chili, Chile, dried ground	La Anita, Mexico	Mexico	7.15	3
Chili, dried	Santa Maria, Sweden	Norway	7.63	3
Chili, dried ground	Spice Cargo	Mexico	12.21	3
Chili, dried ground	Rajah	Norway	7.87	3
Chili, dried ground	Engebretsen AS, Norway	Norway	12.15	3
Chili, dried ground	USA	USA	8.37	6
Chili, dried ground, hot	Hindu, Norway	Norway	5.96	3
Chili, dried ground, mexican	Hindu, Norway	Norway	11.86	3
Chili, green, whole	Spain	Norway	2.33	3
Chili, red with seeds, dried	India	India	2.52	3
Chili, red, whole	Spain	Norway	2.92	3
Chili, red, whole		Norway	2.08	3
Chili, without seeds, dried	India	India	3.74	3
Chives, chopped, dried	Spice Cargo	Mexico	7.80	3
Chives, dried	Black Boy, Rieber og søn	Norway	7.11	3
Chives, dried	Natures Treats Australia PTY LTD, Australia	New Zealand	2.56	3
Chives, dried	Norsk Øko-Urt AB, Norway	Norway	11.14	5
Chives, fresh	BAMA gruppen, Norway	Norway	0.59	3
Chives, fresh		Norway	0.60	3
Cinnamon sticks, Cassia vera indo	Natures Treats Australia PTY LTD, Australia	New Zealand	6.84	3
Cinnamon, bark, whole	Mexico	Mexico	40.14	3
Cinnamon, bark, whole	Asian Bazaar	Mexico	32.61	3
Cinnamon, dried ground	India	India	31.64	3
Cinnamon, dried ground	Spice Cargo	Mexico	139.89	3
Cinnamon, dried ground		USA	17.65	6
Cinnamon, dried ground	Black Boy, Rieber og søn	Norway	53.04	3
Cinnamon, dried ground	Engebretsen AS, Norway	Norway	63.27	3

Category 22 Spices and herbs continued

Product	Manufacturer / product label / country of origin	Procured in	Antioxidant content in mmol/100g	Comment
Cinnamon, dried ground	Santa Maria, Sweden	Norway	118.69	3
Cinnamon, dried ground	Canela Molida	Mexico	114.98	3
Cirsium heterohpyllum, leaves, dried	The Norwegian Crop Research Institute, Norway	Norway	38.18	5
Clove, dried ground		USA	125.55	6
Clove, dried ground	Black Boy, Rieber og søn	Norway	465.32	3
Clove, whole, dried	La Surtidora	Mexico	175.31	3
Clove, whole, dried	TRS Wholesale CO, England	Norway	317.96	3
Clove, whole, dried	Escosa, Mexico	Mexico	327.77	3
Clove, whole, dried	India	India	252.04	3
Club-moss, dried	The Norwegian Crop Research Institute, Norway	Norway	4.56	5
Coltsfoot, leaves, dried	The Norwegian Crop Research Institute, Norway	Norway	61.32	5
Columbine, Granny's bonnet, dried	The Norwegian Crop Research Institute, Norway	Norway	3.96	5
Common alkanet, dried	The Norwegian Crop Research Institute, Norway	Norway	18.37	5
Common butterwort, leaves, dried	The Norwegian Crop Research Institute, Norway	Norway	41.93	5
Common chickweed, dried	The Norwegian Crop Research Institute, Norway	Norway	5.17	5
Common elder, flower, dried	The Norwegian Crop Research Institute, Norway	Norway	24.13	5
Common elder, leaves, dried	The Norwegian Crop Research Institute, Norway	Norway	20.36	5
Common fumitory, dried	The Norwegian Crop Research Institute, Norway	Norway	25.06	5

Category 22 Spices and herbs continued

Product	Manufacturer / product label / country of origin	Procured in	Antioxidant content in mmol/100g	Comment
Common horsetail, dried	The Norwegian Crop Research Institute, Norway	Norway	12.17	5
Common mallow, flower and leaves, dried	The Norwegian Crop Research Institute, Norway	Norway	9.06	5
Common mallow, flower, dried	Norsk Øko-Urt AB, Norway	Norway	24.63	5
Common mallow, leaves, dried	Norsk Øko-Urt AB, Norway	Norway	9.20	5
Common nettle, stinging nettle, leaves, dried	Norsk Øko-Urt AB, Norway	Norway	35.23	5
Common polypody, rhizome	The Norwegian Crop Research Institute, Norway	Norway	35.42	5
Common silver birch, leaves, dried	The Norwegian Crop Research Institute, Norway	Norway	22.07	5
Common valerian, flower and leaves, dried	The Norwegian Crop Research Institute, Norway	Norway	24.03	5
Condiment with red pepper, dried ground	Japan	Japan	5.23	3
Coriander (Dhaniya), dried ground	Rajah	Norway	4.66	3
Coriander seeds	Onena Spices, Spain	Norway	0.28	3
Coriander seeds	Asian Bazaar	Mexico	1.26	3
Coriander, leaves, dried	Santa Maria, Sweden	Norway	2.84	3
Coriander, leaves, dried	Black Boy, Rieber og søn	Norway	2.10	3
Coriander, leaves, fresh	BAMA gruppen, Norway	Norway	1.20	3
Coriander, leaves, fresh	Norway	Norway	0.41	3
Coriander, seeds, green, dried	India	India	3.49	3
Cornflower, dried	The Norwegian Crop Research Institute, Norway	Norway	11.96	5
Cornflower, flower, dried	Norsk Øko-Urt AB, Norway	Norway	8.84	5

Category 22 Spices and herbs continued

Product	Manufacturer / product label / country of origin	Procured in	Antioxidant content in mmol/100g	Comment
Creeping jenny (Lysimachia nummularia), leaves, dried	The Norwegian Crop Research Institute, Norway	Norway	31.31	5
Cumin, Comino, dried ground	Mexico	Mexico	8.23	3
Cumin, dried ground	Black Boy, Rieber og søn	Norway	6.82	3
Cumin, dried ground	Spice Cargo	Mexico	9.14	3
Cumin, dried ground	Comino Molido	Mexico	10.30	3
Cumin, Jerra, dried ground	Rajah	Norway	11.88	3
Cumin, seeds, whole, dried	Santa Maria, Sweden	Norway	2.54	3
Cumin, whole	Hindu, Norway	Norway	2.45	3
Curled parsley, fresh	Hafskjold Gartneri, Norway	Norway	0.34	3
Curry, powder	Japan	Japan	10.47	3
Curry, powder	Spice Cargo	Mexico	14.92	3
Curry, powder	USA	USA	9.98	6
Curry, powder	TRS Wholesale CO, England	Norway	10.93	3
Curry, powder	Black Boy, Rieber og søn	Norway	13.02	3
Curry, powder, Madras, hot, dried ground	Rajah	Norway	6.65	3
Curry, powder, Madras, mild, dried ground	Rajah	Norway	7.43	3
Curry, powder, Premium, mild, dried ground	Rajah	Norway	4.17	3
Dame's violet, dried	The Norwegian Crop Research Institute, Norway	Norway	22.63	5
Dandelion, flower, dried	Norsk Øko-Urt AB, Norway	Norway	12.72	5
Dandelion, leaves	Norway	Norway	6.89	4
Dandelion, leaves, dried	The Norwegian Crop Research Institute, Norway	Norway	21.07	5
Dandelion, root	The Norwegian Crop Research Institute, Norway	Norway	4.91	5
Devil's-bit, dried	The Norwegian Crop Research Institute, Norway	Norway	30.18	5

Category 22 Spices and herbs continued

Product	Manufacturer / product label / country of origin	Procured in	Antioxidant content in mmol/100g	Comment
Dill, dried	Norsk Øko-Urt AB, Norway	Norway	20.23	5
Dill, dried	Goutess, GmbH	Norway	24.47	3
Dill, dried	Black Boy, Rieber og søn	Norway	15.94	3
Dill, dried	Hindu, Norway	Norway	2.79	
Dill, dried	Santa Maria	Norway	2.68	
Dill, fresh	Norway	Norway	1.39	3
Dill, fresh		Norway	2.18	3
Dill, seeds	The Norwegian Crop Research Institute, Norway	Norway	3.37	5
Dwarf birch, leaves, dried	The Norwegian Crop Research Institute, Norway	Norway	86.22	5
English ivy, leaves, dried	The Norwegian Crop Research Institute, Norway	Norway	27.98	5
Estragon, dried	Black Boy, Rieber og søn	Norway	43.31	3
Estragon, dried	Santa Maria	Norway	13.63	
Estragon, french, leaves, dried	Norsk Øko-Urt AB, Norway	Norway	43.22	5
Estragon, russian, leaves, dried	Norsk Øko-Urt AB, Norway	Norway	44.75	5
European golden rod, dried	The Norwegian Crop Research Institute, Norway	Norway	28.43	5
Fakouhoye leaves, dried	Mali	Mali	10.20	3
Fennel, leaves, dried	Norsk Øko-Urt AB, Norway	Norway	18.91	5
Fennel, whole seeds, dried	TRS Wholesale CO, England	Norway	5.84	3
Fenugreek, seeds	India	India	2.09	3
Fenugreek, whole	Asian Bazaar	Mexico	1.67	3
Field bindweed, dried	The Norwegian Crop Research Institute, Norway	Norway	17.51	5
Field forget-me-not, dried	The Norwegian Crop Research Institute, Norway	Norway	28.15	5

Category 22 Spices and herbs continued

Product	Manufacturer / product label / country of origin	Procured in	Antioxidant content in mmol/100g	Comment
Field horsetail (Equisetum arvense), leaves, dried	Norsk Øko-Urt AB, Norway	Norway	9.41	5
Field restharrow (Ononis arvensis), root	The Norwegian Crop Research Institute, Norway	Norway	10.15	5
Figwort, dried	The Norwegian Crop Research Institute, Norway	Norway	8.69	5
Fir clubmoss, dried	The Norwegian Crop Research Institute, Norway	Norway	10.58	5
Garden Cat-mint (Nepeta x faassenii), dried	The Norwegian Crop Research Institute, Norway	Norway	14.18	5
Garlic, dried ground		USA	0.80	6
Garlic, dried ground	Black Boy, Rieber og søn	Norway	2.13	3
Garlic, dried ground	Rajah	Norway	1.61	3
Garlic, dried ground	Spice Cargo	Mexico	0.78	3
Garlic, raw paste	Japan	Japan	0.08	3
Ginger		Norway	2.79	3
Ginger	Mali	Mali	3.93	3
Ginger (jengibre molido), dried ground		Mexico	22.19	3
Ginger, dried	India	India	11.31	3
Ginger, dried ground	Santa Maria, Sweden	Norway	22.12	3
Ginger, dried ground	Northwest Delights, USA	Norway	0.86	3
Ginger, dried ground	Spice Cargo	Mexico	24.37	3
Ginger, dried ground		USA	21.57	6
Ginger, raw paste	Japan	Japan	5.33	3
Grass-of-Parnasuss (Parnassia palustris), dried	The Norwegian Crop Research Institute, Norway	Norway	52.27	5
Greater burdock, root	The Norwegian Crop Research Institute, Norway	Norway	14.26	5
Greater plantain, leaves, dried	The Norwegian Crop Research Institute, Norway	Norway	22.03	5

Category 22 Spices and herbs continued

Product	Manufacturer / product label / country of origin	Procured in	Antioxidant content in mmol/100g	Comment
Green mint, leaves, dried	Norsk Øko-Urt AB, Norway	Norway	142.58	5
Grey alder (Alnus incana), leaves, dried	The Norwegian Crop Research Institute, Norway	Norway	59.27	5
Ground-ivy (Glechoma hederacea), dried	The Norwegian Crop Research Institute, Norway	Norway	31.72	5
Hazel, leaves, dried	The Norwegian Crop Research Institute, Norway	Norway	35.51	5
Heather, flower, dried	The Norwegian Crop Research Institute, Norway	Norway	56.98	5
Hoary plantain, leaves, dried	The Norwegian Crop Research Institute, Norway	Norway	29.35	5
Hollyhock, flower and leaves, dried	The Norwegian Crop Research Institute, Norway	Norway	10.16	5
Hop, cone	The Norwegian Crop Research Institute, Norway	Norway	30.96	5
Hops, leaves, dried	Norsk Øko-Urt AB, Norway	Norway	35.28	5
Horehound (Marrubium vulgare), dried	The Norwegian Crop Research Institute, Norway	Norway	12.49	5
Hound's tongue, leaves, dried	The Norwegian Crop Research Institute, Norway	Norway	32.65	5
Houseleek, dried	The Norwegian Crop Research Institute, Norway	Norway	5.24	5
Hyssop, flower, dried	Norsk Øko-Urt AB, Norway	Norway	52.29	5
Hyssop, leaves, dried	Norsk Øko-Urt AB, Norway	Norway	44.90	5
Iceland moss (Cetraria islandica), dried	The Norwegian Crop Research Institute, Norway	Norway	0.71	5

Category 22 Spices and herbs continued

Product	Manufacturer / product label / country of origin	Procured in	Antioxidant content in mmol/100g	Comment
Imperatoria ostruthium, rhizome	The Norwegian Crop Research Institute, Norway	Norway	27.56	5
Jalapeño Pepper, dried	Black Boy, Rieber og søn	Norway	8.25	3
Japanese pepper, dried ground	Japan	Japan	36.92	3
Japanese rose, Ramanas rose, fruit shell, dried	The Norwegian Crop Research Institute, Norway	Norway	58.66	5
Juniper berries, blue, dried	The Norwegian Crop Research Institute, Norway	Norway	19.29	5
Juniper berries, coniferous litter, dried	The Norwegian Crop Research Institute, Norway	Norway	76.77	5
Juniper berries, dried	Norsk Øko-Urt AB, Norway	Norway	8.89	5
Juniper berries, dried	Black Boy, Rieber og søn	Norway	9.27	3
Juniper berries, green, dried	Norsk Øko-Urt BA, Norway	Norway	8.42	5
Juniper berries, green, dried	The Norwegian Crop Research Institute, Norway	Norway	80.26	5
Kaloonji, whole seeds, dried	Ashiq Cash&Carry, England	Norway	1.02	3
Knotgrass, dried	The Norwegian Crop Research Institute, Norway	Norway	16.62	5
Lady's bedstraw, dried	The Norwegian Crop Research Institute, Norway	Norway	9.90	5
Lady's mantle, leaves, dried	Norsk Øko-Urt AB, Norway	Norway	43.31	5
Lady's mantle, leaves, dried	The Norwegian Crop Research Institute, Norway	Norway	24.99	5
Lavender, leaves and flower, dried	Norsk Øko-Urt AB, Norway	Norway	29.61	5
Lemon balm (Melissa officinalis), leaves, fresh	Norway	Norway	1.32	3

Category 22 Spices and herbs continued

Product	Manufacturer / product label / country of origin	Procured in	Antioxidant content in mmol/100g	Comment
Lemon balm, leaves, dried	Norsk Øko-Urt AB, Norway	Norway	125.33	5
Lemon pepper	Santa Maria	Norway	0.66	
Lemon pepper	Hindu, Norway	Norway	1.00	
Lemon thyme, leaves and flower, dried	Norsk Øko-Urt AB, Norway	Norway	92.18	5
Lemon thyme, leaves, dried	Norsk Øko-Urt AB, Norway	Norway	9.22	5
Liquorice, sweet-root, root and rhizome	The Norwegian Crop Research Institute, Norway	Norway	2.71	5
Lovage (Levisticum officinale), leaves, dried	The Norwegian Crop Research Institute, Norway	Norway	23.70	5
Lovage (Levisticum officinale), leaves, dried	Norsk Øko-Urt AB, Norway	Norway	36.17	5
Maghaj, dried	India	India	0.27	3
Maral Root (Leuzea carthamoides), leaves, dried	The Norwegian Crop Research Institute, Norway	Norway	69.57	5
Marigold (Calendula officinalis), flower and leaves, dried	The Norwegian Crop Research Institute, Norway	Norway	9.83	5
Meadowsweet (Filipendula ulmaria), dried	The Norwegian Crop Research Institute, Norway	Norway	154.05	5
Meadowsweet (Filipendula ulmaria), flower and leaves, dried	The Norwegian Crop Research Institute, Norway	Norway	117.77	5
Meadowsweet (Filipendula ulmaria), flower, dried	Norsk Øko-Urt AB, Norway	Norway	167.82	5
Meadowsweet (Filipendula ulmaria), leaves, dried	Norsk Øko-Urt AB, Norway	Norway	111.30	5
Merian, dried	Santa Maria, Sweden	Norway	53.92	3
Mint, dried	Onena Spices, Spain	Norway	71.95	3
Mint, Mentha spicata, fresh		Norway	1.27	3
Motherworth (Leonurus cardiaca), dried	The Norwegian Crop Research Institute, Norway	Norway	13.19	5
Mugwort, dried	The Norwegian Crop Research Institute, Norway	Norway	23.79	5

Category 22 Spices and herbs continued

Product	Manufacturer / product label / country of origin	Procured in	Antioxidant content in mmol/100g	Comment
Mullein, flower, dried	The Norwegian Crop Research Institute, Norway	Norway	37.71	5
Mustard powder	Spice Cargo	Mexico	10.30	3
Mustard powder	Colman's	Norway	10.39	3
Mustard seed, yellow, ground		USA	10.53	6
Mustard seeds	India	India	3.78	3
Mustard seeds, brown, whole	Asian Bazaar	Mexico	6.70	3
Mustard seeds, ground	Mexico	Mexico	9.44	3
Mustard seeds, yellow, whole	Asian Bazaar	Mexico	7.52	3
Nettle, White Deaed, dried	The Norwegian Crop Research Institute, Norway	Norway	18.21	5
Northern dock, dried	The Norwegian Crop Research Institute, Norway	Norway	43.61	5
Northern dock, root	The Norwegian Crop Research Institute, Norway	Norway	56.69	5
Nutmeg (Jalwatri), dried	Roopaks, Ajmal Khan, N. Dehli	India	19.42	3
Nutmeg, dried	India	India	33.00	3
Nutmeg, dried ground	Black Boy, Rieber og søn	Norway	20.32	3
Nutmeg, dried ground	Mexico	Mexico	43.52	3
Nutmeg, whole, dried	Jaifal, England	Norway	15.83	3
Onion, dried ground		USA	0.95	6
Oregano (oregano entero), dried	La Surtidora	Mexico	73.77	3
Oregano, dried		USA	40.30	6
Oregano, dried	Greece	Norway	45.58	3
Oregano, dried	Norsk Øko-Urt AB, Norway	Norway	89.51	5
Oregano, dried	Mexico	Mexico	47.64	3
Oregano, dried	Gökqehan, Turkey	Norway	96.64	3
Oregano, dried	Hindu, Norway	Norway	48.02	3
Oregano, dried	Black Boy, Rieber og søn	Norway	44.99	3
Oregano, dried	Santa Maria	Norway	21.42	
Oregano, dried	McCormick	Mexico	82.61	3

Category 22 Spices and herbs continued

Product	Manufacturer / product label / country of origin	Procured in	Antioxidant content in mmol/100g	Comment
Oregano, fresh	Norway	Norway	3.75	3
Oregano, fresh	Gartner, BAMA, Norway	Norway	3.81	3
Orpine (Sedum telephium), rhizome	The Norwegian Crop Research Institute, Norway	Norway	57.83	5
Paprika (powder), dried ground	Rajah	Norway	6.78	3
Paprika (powder), dried ground		USA	8.60	6
Paprika (powder), dried ground	Santa Maria, Sweden	Norway	5.93	3
Paprika (powder), dried ground	Paprika Molido	Mexico	7.44	3
Paprika (powder), dried ground	Engebretsen AS, Norway	Norway	8.08	3
Paprika, (powder), dried ground	Black Boy, Rieber og søn	Norway	5.59	3
Paprika, (powder), red, dried ground	Spice Cargo	Mexico	5.75	3
Parsely, big leaves, fresh	Linnes gård, Norway	Norway	1.93	3
Parsley	Mali	Mali	1.12	3
Parsley	Norway	Norway	0.86	3
Parsley	Sweden	Norway	1.22	5
Parsley	Lier	Norway	2.00	3
Parsley, big leaves, fresh	Sweden	Norway	2.03	5
Parsley, dried	Hindu, Norway	Norway	3.72	
Parsley, dried		USA	7.43	6
Parsley, dried	Norsk Øko-Urt AB, Norway	Norway	10.09	5
Parsley, dried	Black Boy, Rieber og søn	Norway	3.64	3
Parsley, dried	Santa Maria	Norway	3.81	
Parsley, dried	Spice Cargo	Mexico	8.23	3
Pepper (pimenta dulce molida), dried ground	McCormick	Mexico	50.96	3
Pepper, black (pimenta negra molida), dried ground	La Surtidora	Mexico	5.08	3
Pepper, black, dried ground	Spice Cargo	Mexico	6.68	3
Pepper, black, dried ground	Rajah	Norway	6.65	3
Pepper, black, dried ground	Black Boy, Rieber og søn	Norway	8.71	3

Category 22 Spices and herbs continued

Product	Manufacturer / product label / country of origin	Procured in	Antioxidant content in mmol/100g	Comment
Pepper, black, dried ground		USA	4.54	6
Pepper, black, whole, dried	India	India	4.15	3
Pepper, black, whole, dried		USA	4.34	6
Pepper, dark green "berries" on the stem, fresh	Thailand	Norway	0.26	3
Pepper, green "berries" on the stem, fresh	Thailand	Norway	0.46	3
Pepper, white, dried ground	Santa Maria	Norway	3.92	3
Pepper, white, dried ground	Rajah	Norway	5.02	3
Pepper, white, whole	Roopaks, Ajmal Khan, N. Dehli	India	3.49	3
Peppermint, leaves, dried	Norsk Øko-Urt AB, Norway	Norway	160.82	5
Pepperwort, garden cress, fresh		Norway	2.42	3
Perforate St. John's wort, flower and leaves, dried	The Norwegian Crop Research Institute, Norway	Norway	54.37	5
Piffi, dried ground	Engebretsen AS, Norway	Norway	0.61	3
Piri Piri, dried ground	Santa Maria	Norway	9.39	3
Piri-piri, dried	Black Boy, Rieber og søn	Norway	6.51	3
Pot marigold, flower, dried	Norsk Øko-Urt AB, Norway	Norway	11.47	5
Purple Coneflower, flower and leaves, dried	Norsk Øko-Urt AB, Norway	Norway	16.09	5
Purple Loosestrife (Lythrum salicaria), flower and leaves, dried	The Norwegian Crop Research Institute, Norway	Norway	111.04	5
Quack grass, rootstock (Elytrigia repens, rhizome)	The Norwegian Crop Research Institute, Norway	Norway	0.88	5
Rai, dried	India	India	2.84	3
Raspberry, leaves, dried	Norsk Øko-Urt AB, Norway	Norway	46.89	5
Raspberry, leaves, dried	The Norwegian Crop Research Institute, Norway	Norway	32.56	5
Raspberry, leaves, fresh	Norway	Norway	21.36	4
Red clover, flower, dried	Norsk Øko-Urt AB, Norway	Norway	39.92	5

Category 22 Spices and herbs continued

Product	Manufacturer / product label / country of origin	Procured in	Antioxidant content in mmol/100g	Comment
Red wortleberries, leaves, dried	The Norwegian Crop Research Institute, Norway	Norway	102.07	5
Red-berried elder, leaves, dried	The Norwegian Crop Research Institute, Norway	Norway	56.66	5
Ribwort, leaves, dried	The Norwegian Crop Research Institute, Norway	Norway	34.81	5
Rose, flower, dried	Norsk Øko-Urt AB, Norway	Norway	153.90	5
Rose-bay, leaves, dried	Norsk Øko-Urt AB, Norway	Norway	101.33	5
Rose-bay, willow herb flower, dried	Norsk Øko-Urt AB, Norway	Norway	93.48	5
Rose-bay, willow herb, flower and leaves, dried	The Norwegian Crop Research Institute, Norway	Norway	120.99	5
Rosemary, dried	Hindu, Norway	Norway	35.81	3
Rosemary, dried	Black Boy, Rieber og søn	Norway	66.92	3
Rosemary, fresh	Norway	Norway	5.64	3
Rosemary, fresh leaves	Gartner, BAMA, Norway	Norway	11.07	3
Rosemary, fresh leaves	BAMA gruppen, Norway	Norway	6.34	3
Rosemary, leaves, dried	Norsk Øko-Urt AB, Norway	Norway	56.95	5
Rosemary, leaves, dried	Spice Cargo	Mexico	39.99	3
Rosemary, leaves, dried	Gökqehan, Turkey	Norway	24.34	3
Roseroot, fresh	The Norwegian Crop Research Institute, Norway	Norway	5.63	5
Saffron, Balaji, dried ground	Delhi Keshar co.	India	23.83	3
Saffron, dried ground	Roopaks, Ajmal Khan, N. Dehli	India	61.72	3
Saffron, dried ground	Gaea, Greece	Norway	47.83	3
Saffron, stigma	Mexico	Mexico	7.02	3
Saffron, stigma	Carmencita, Spain	Norway	24.83	3
Saffron, stigma	Bahraman Saffron, Iran	Iran	20.58	3
Sage, dried	Hindu, Norway	Norway	34.88	3
Sage, dried	Spice Cargo	Mexico	58.80	3

Category 22 Spices and herbs continued

Product	Manufacturer / product label / country of origin	Procured in	Antioxidant content in mmol/100g	Comment
Sage, leaves, dried	Norsk Øko-Urt AB, Norway	Norway	39.36	5
Sanguisorba officinalis, dried	The Norwegian Crop Research Institute, Norway	Norway	33.37	5
Saunf, big, dried	India	India	7.09	3
Saunf, small, dried	India	India	6.46	3
Scented mayweed, flower, dried	Norsk Øko-Urt AB, Norway	Norway	16.63	5
Shah jerra, dried	Roopaks, Ajmal Khan, N. Dehli	India	5.34	3
Shepherd's purse, dried	The Norwegian Crop Research Institute, Norway	Norway	5.52	5
Silverweed, dried	The Norwegian Crop Research Institute, Norway	Norway	35.79	5
Small-leaved lime, flower, dried	The Norwegian Crop Research Institute, Norway	Norway	34.83	5
Somage, dried ground	Iran	Iran	85.58	3
Sorrel, leaves, dried	The Norwegian Crop Research Institute, Norway	Norway	19.52	5
Sorrel, Wood (Oxalis acetosella), dried	The Norwegian Crop Research Institute, Norway	Norway	6.54	5
Southernwood, flower, stem and leaves, dried	The Norwegian Crop Research Institute, Norway	Norway	34.88	5
Spanish chervil, leaves, dried	Norsk Øko-Urt AB, Norway	Norway	54.96	5
Speedwell, dried	The Norwegian Crop Research Institute, Norway	Norway	94.79	5
Spice mix, Kjøkkensjef Natvigs	Black Boy, Rieber og søn	Norway	0.39	
Spice mix, Aromat	Knorr	Norway	0.27	
Spice mix, Gastromat	Gastromat A/S, Norway	Norway	0.60	
Spicemix, taco	ICA, Sweden	Norway	2.63	3
Spicemix, taco, original, TexMex	Santa Maria, Sweden	Norway	4.11	3

Category 22 Spices and herbs continued

Product	Manufacturer / product label / country of origin	Procured in	Antioxidant content in mmol/100g	Comment
Spicemix, tacos	Old El Paso	Norway	3.80	3
Spruce, leaves, dried	Norsk Øko-Urt AB, Norway	Norway	29.31	5
St. John's wort, flower and leaves, dried	The Norwegian Crop Research Institute, Norway	Norway	72.16	5
Star anise, dried	India	India	11.30	3
Stevia Drypp, juice from fermented leaves	Medurt Pharma	Norway	3.28	
Stevia rebaudiana, dried leaves	Medurt Pharma	Norway	63.55	
Stevia rebaudiana, fermented leaves	Medurt Pharma	Norway	3.17	
Stevia rebaudiana, leaves	Medurt Pharma	Norway	6.27	
Stevia rebaudiana, leaves		Norway	14.25	4
Stinging nettle, dried	The Norwegian Crop Research Institute, Norway	Norway	13.09	5
Stinging nettle, leaves	Norway	Norway	3.66	4
Sugar kelp, dried, Kombu Royal	Iceland	Iceland	0.26	
Summac, dried ground	Khater Spice, Lebanon	Norway	42.36	3
Summer savory, leaves and flower, dried	Norsk Øko-Urt AB, Norway	Norway	59.66	5
Sundew (Drosera angelica), dried	The Norwegian Crop Research Institute, Norway	Norway	79.02	5
Sundew (Drosera rotundifolia), dried	The Norwegian Crop Research Institute, Norway	Norway	85.97	5
Sweet marjoram, leaves, dried	Norsk Øko-Urt AB, Norway	Norway	92.31	5
Tamarind	India	India	3.50	3
Tansy, flower, dried	The Norwegian Crop Research Institute, Norway	Norway	30.71	5
Tej Pata (bay leaves), dried	India	India	18.54	3
Thribi, dried	Greece	Norway	42.56	3
Thyme, dried	Black Boy, Rieber og søn	Norway	63.75	3
Thyme, dried	Greece	Norway	42.00	3

Category 22 Spices and herbs continued

Product	Manufacturer / product label / country of origin	Procured in	Antioxidant content in mmol/100g	Comment
Thyme, dried	Norsk Øko-Urt AB, Norway	Norway	63.13	5
Thyme, fresh	Norway	Norway	2.16	3
Thyme, fresh leaves	Gartner, BAMA, Norway	Norway	2.65	3
Thyme, fresh leaves	BAMA gruppen, Norway	Norway	1.46	3
Trembling poplar, Aspen, leaves, dried	The Norwegian Crop Research Institute, Norway	Norway	26.65	5
Turmeric, dried ground		USA	15.68	6
Turmeric, dried ground	Black Boy, Rieber og søn	Norway	10.25	3
Turmeric, dried ground	Rajah	Norway	10.92	3
Turmeric, dried ground	Unifood, India	Norway	10.55	3
Turmeric, dried ground	Spice Cargo	Mexico	15.63	3
Turmeric, Haldi, dried ground	Rajah	Norway	11.83	3
Turmeric, whole, dried	India	India	13.60	3
Vanilla pod, seeds from pod	Onena Spices, Spain	Norway	3.73	3
Vanilla pod, seeds from pod	Tørsleffs, Haugen-gruppen	Norway	5.15	3
Vanilla pod, whole with seeds	Onena Spices, Spain	Norway	7.13	3
Vanilla pod, whole with seeds	Tørsleffs, Haugen-gruppen	Norway	7.38	3
Vanilla pod, whole with seeds	Black Boy, Rieber og søn	Norway	7.16	3
Vanilla pod, without seeds	Black Boy, Rieber og søn	Norway	10.09	3
Vanilla pod, without seeds	Onena Spices, Spain	Norway	8.69	3
Vanilla pod, without seeds	Tørsleffs, Haugen-gruppen	Norway	8.50	3
Vanilla, seeds from pod	Black Boy, Rieber og søn	Norway	2.59	3
Viola canina, leaves, dried	The Norwegian Crop Research Institute, Norway	Norway	12.90	5
Wall germander (Teucrium chamaedrys), dried	The Norwegian Crop Research Institute, Norway	Norway	48.14	5
Wasabi, paste	Japan	Japan	0.11	3

Category 22 Spices and herbs continued

Product	Manufacturer / product label / country of origin	Procured in	Antioxidant content in mmol/100g	Comment
Wild marjoram, leaves, dried	Norsk Øko-Urt AB, Norway	Norway	131.92	5
Wild marjoram, leaves, dried	The Norwegian Crop Research Institute, Norway	Norway	142.86	5
Woodland geranium (Geranium sylvaticum), dried	The Norwegian Crop Research Institute, Norway	Norway	113.27	5
Wormwood, absinth, dried	The Norwegian Crop Research Institute, Norway	Norway	10.42	5
Wych elm, leaves, dried	The Norwegian Crop Research Institute, Norway	Norway	15.65	5
Yarrow, flower and leaves, dried	The Norwegian Crop Research Institute, Norway	Norway	31.66	5
Yarrow, flower, dried	Norsk Øko-Urt AB, Norway	Norway	18.61	5
Yellow Loosestrife (Lysimacha vulgaris), leaves, dried	The Norwegian Crop Research Institute, Norway	Norway	72.96	5
Yellow sweet clover, flower and leaves, dried	The Norwegian Crop Research Institute, Norway	Norway	5.14	5

Category 23 Vegetables

Product	Manufacturer / product label / country of origin	Procured in	Antioxidant content in mmol/100g	Comment
Alfa sprouts	Natural Spirer	Norway	0.14	3
Artichoke	Italy	Norway	0.69	3
Artichoke, boiled	Other Brand	USA	3.89	6
Artichoke, boiled	Ocean Mist	USA	4.54	6
Artichoke, brine pack	Delallo	USA	3.36	6
Artichoke, canned	Mario's, Spain	Norway	4.76	3
Artichoke, leaves	Italy	Norway	1.66	3
Artichoke, microwaved	Ocean Mist	USA	4.69	6
Artichoke, water pack	Progresso	USA	4.32	6
Asparagus		USA	0.36	6
Asparagus	Agro Paracas, Peru	Norway	0.85	3
Asparagus bean, frozen	Hakon, Norway	Norway	0.34	3
Asparagus bean, frozen, cooked	Hakon, Norway	Norway	0.24	3
Asparagus, cooked		USA	0.75	6
Aubergine	Netherlands	Norway	0.25	3
Aubergine	Mali	Mali	0.07	3
Aubergine	Italy	Norway	0.18	3
Aubergine, native, red	Mali	Mali	0.17	3
Aubergine, native, white	Mali	Mali	0.07	3
Avocado		USA	0.41	1, 6
Avocado		Norway	0.44	1, 3
Baby carrots		USA	0.04	6
Beans with tomato sauce, canned	Diva, Italy	Norway	0.16	3
Beet (beetroot)	Norway	Norway	1.68	3
Beet (beetroot)	Finstad Gård, Sande, Norway	Norway	1.41	3
Broccoli		Norway	0.85	3
Broccoli		USA	0.25	6
Broccoli	Spain	Norway	0.68	3
Broccoli	Sweden	Norway	0.30	3
Broccoli raab		USA	0.65	6
Broccoli raab, cooked		USA	0.97	6
Broccoli, cooked		Norway	0.91	3

Category 23 Vegetables continued

Product	Manufacturer / product label / country of origin	Procured in	Antioxidant content in mmol/100g	Comment
Broccoli, cooked		USA	1.00	6
Brussels sprout, Content	Norwegian University of Life Sciences	Norway	0.74	5
Brussels sprouts	BAMA gruppen, Holland	Norway	0.89	3
Brussels sprouts		Norway	1.33	3
Cabbage	Norway	Norway	0.15	3
Cabbage		USA	0.10	6
Cabbage	Mali	Mali	0.02	3
Cabbage, cooked		USA	0.45	6
Cabbage, Lady	Norwegian University of Life Sciences	Norway	0.10	5
Cabbage, red	Norway	Norway	1.78	3
Cabbage, red		USA	0.80	6
Cabbage, red	Dole	Norway	1.61	5
Cabbage, red, Autoro	Norwegian University of Life Sciences	Norway	2.09	5
Cabbage, red, cooked		USA	2.15	6
Cabbage, red, from packaged Classic Iceberg salad, chopped	Dole	Norway	1.05	5
Cantaloupe		USA	0.25	6
Carrot drink	Eckes-Granini	Norway	0.28	3
Carrot juice	Bræmhults, Sweden	Norway	0.06	3
Carrots	Dole	Norway	0.06	5
Carrots	Sweden	Sweden	0.09	5
Carrots	Mali	Mali	0.02	3
Carrots		USA	0.03	6
Carrots, cooked		USA	0.10	6
Carrots, cut, frozen	Birds Eye	USA	0.05	6
Carrots, cut, frozen	Store Brand	USA	0.06	6
Carrots, cut, frozen, boiled	Store Brand	USA	0.07	6
Carrots, cut, frozen, microwaved	Store Brand	USA	0.08	6
Carrots, from packaged Classic Iceberg salad, chopped	Dole	Norway	0.02	5

Category 23 Vegetables continued

Product	Manufacturer / product label / country of origin	Procured in	Antioxidant content in mmol/100g	Comment
Carrots, frozen	Birds Eye	USA	0.07	6
Carrots, frozen	Store Brand	USA	0.07	6
Carrots, frozen, boiled	Store Brand	USA	0.07	6
Carrots, frozen, boiled	Birds Eye	USA	0.08	6
Carrots, frozen, microwaved	Store Brand	USA	0.08	6
Carrots, frozen, microwaved	Birds Eye	USA	0.09	6
Carrots, Nantes Duke	Norwegian University of Life Sciences	Norway	0.05	5
Carrots, red, in syrup	Roopaks, Ajmal Khan, N. Dehli	India	0.15	3
Carrots, Yukon	Norwegian University of Life Sciences	Norway	0.06	5
Cassava	Mali	Mali	0.17	3
Cauliflower	Latorre, Spain	Norway	0.35	3
Cauliflower		Norway	0.33	3
Cauliflower, Alverda	Norwegian University of Life Sciences	Norway	0.22	5
Cauliflower, blue	Grafitti	Norway	3.33	3
Cauliflower, blue, cooked	Grafitti	Norway	3.52	3
Cauliflower, boiled		Norway	0.80	3
Cauliflower, Freemont	Norwegian University of Life Sciences	Norway	0.13	5
Celeriac, turnip-rooted celery	Sweden	Norway	0.10	3
Celery		USA	0.06	6
Celery	Mali	Mali	0.81	3
Celery, blanched	Carmel, Israel	Norway	0.00	3
Chinese cabbage	FRUPOR, Portugal	Norway	0.33	3
Chinese cabbage	Norway	Norway	0.47	3
Chinese cabbage	Norgesfrukt, Norway	Norway	0.56	3
Courgettes	Mali	Mali	0.03	3
Cucumber	Mali	Mali	0.02	3
Cucumber		USA	0.04	6

Category 23 Vegetables continued

Product	Manufacturer / product label / country of origin	Procured in	Antioxidant content in mmol/100g	Comment
Cucumber (Cucumis sativus)	Norwegian University of Life Sciences	Norway	0.04	5
Cucumber (Cucumis sativus)	Norway	Norway	0.04	3
Cucumber pickles	Sweets Favorite	USA	0.28	3
Cucumber pickles, whole	Nora, Stabburet, Norway	Norway	0.08	3
Cucumber, small, russian	Øivind Sten, Norway	Norway	0.09	3
Cucumber, without peel		USA	0.02	6
Curly kale		Norway	1.62	3
Curly kale	Sweden	Sweden	2.68	5
Curly kale, Bornick	Norwegian University of Life Sciences	Norway	2.65	5
Curly kale, red	Sweden	Sweden	4.09	5
Edible mushroom (Agaricus bisporus/champignon)		Norway	0.90	3
Edible mushroom (Agaricus bisporus/champignon), canned	Diva	Norway	0.23	3
Fennel	Netherlands	Norway	0.07	3
French fried potatoes, frozen, cooked, steak fries	Ore Ida	USA	0.27	6
French fried potatoes, frozen, crinkle cut	Ore Ida	USA	0.26	6
French fried potatoes, frozen, crinkle cut	Store or Other Brand	USA	0.27	6
French fried potatoes, frozen, crinkle cut, cooked	Ore Ida	USA	0.22	6
French fried potatoes, frozen, crinkle cut, cooked	Store or Other Brand	USA	0.25	6
French fried potatoes, frozen, shoestring	Store or Other Brand	USA	0.26	6
French fried potatoes, frozen, shoestring	Ore Ida	USA	0.38	6
French fried potatoes, frozen, shoestring , cooked	Ore Ida	USA	0.34	6
French fried potatoes, frozen, shoestring, cooked	Store or Other Brand	USA	0.31	6
French fried potatoes, frozen, steak fries	Store or Other Brand	USA	0.23	6

Category 23 Vegetables continued

Product	Manufacturer / product label / country of origin	Procured in	Antioxidant content in mmol/100g	Comment
French fried potatoes, frozen, steak fries, cooked	Store or Other Brand	USA	0.21	6
French fried potatoes, frozen, tater tots	Store or Other Brand	USA	0.28	6
French fried potatoes, frozen, tater tots, cooked	Store or Other Brand	USA	0.22	6
French fried potatoes, frozen, tater tots, seasoned shredded potatoes	Ore Ida	USA	0.10	6
French fried potatoes, frozen, tater tots, seasoned shredded potatoes, cooked	Ore Ida	USA	0.13	6
French fried potatoes, steak fries	Ore Ida	USA	0.26	6
French fries	McDonald's	Norway	0.43	3
French fries	Hakon, Norway	Norway	0.20	3
French fries	McDonald's	USA	0.33	6
French fries	Burger King	USA	0.34	6
French frites, light	Hoff Norske Potetindustrier, Norway	Norway	0.16	3
French toast sticks	Burger King	USA	0.19	6
Garlic		Norway	0.22	3
Garlic	Sweden	Norway	0.06	3
Garlic	Mali	Mali	0.21	3
Garlic	Netherlands	Norway	0.21	3
Hash brown rounds	Burger King	USA	0.24	6
Hash browns	McDonald's	USA	0.35	6
Horse radish		Norway	0.49	3
Horse radish		Norway	0.68	3
Leaves from the African Baobab tree, dry, crushed	Mali	Mali	48.07	3
Leaves of the Pumpkin plant	Malawi	Malawi	1.02	4
Leaves of the Sweet Potato plant	Malawi	Malawi	0.48	4
Leek	Mali	Mali	0.90	3
Leek	Sweden	Norway	0.16	3
Leek	France	Norway	0.25	3
Lettuce, Butterhead		USA	0.13	6

Category 23 Vegetables continued

Product	Manufacturer / product label / country of origin	Procured in	Antioxidant content in mmol/100g	Comment
Lettuce, Crispheaded	Norwegian University of Life Sciences	Norway	0.07	5
Lettuce, Endevie	France	Norway	0.10	3
Lettuce, Green leaves		USA	0.24	6
Lettuce, Iceberg	Dole	Norway	0.02	5
Lettuce, Iceberg		USA	0.17	6
Lettuce, Iceberg, from packaged Classic Iceberg salad, chopped	Dole	Norway	0.01	5
Lettuce, Lollo rosso	Norwegian University of Life Sciences	Norway	0.60	5
Lettuce, Red leaves		USA	0.23	6
Lettuce, Romaine		USA	0.21	6
Mixed vegetables	Landlord, Norway	Norway	0.31	3
Mixed vegetables	Hakon, Norway	Norway	0.29	3
Mixed vegetables	Produced in Norway for Coop Norge	Norway	0.29	3
Moringa Stenopetala, dried leaves and stem	Ethiopia	Ethiopia	11.90	4
Moringa Stenopetala, fresh green leaves and stem	Ethiopia	Ethiopia	3.70	4
Mushroom (Pholiota mutabilis)	Norway	Norway	0.50	4
Mushroom, Chanterelle, wild	Norway	Norway	0.30	4
Mushroom, Crimini		USA	0.25	6
Mushroom, Edible bolete	Norway	Norway	0.61	4
Mushroom, Enoki	Phillips Mushrooms	USA	0.31	6
Mushroom, Enoki		USA	0.25	6
Mushroom, Enoki	Monterrey Mushrooms	USA	0.33	6
Mushroom, Funnel chantarelle, wild	Norway	Norway	0.68	4
Mushroom, Hedgehog fungus	Norway	Norway	0.27	4
Mushroom, Maitake	Monterrey Mushrooms	USA	0.23	6
Mushroom, Maitake	Phillips Mushrooms	USA	0.11	6
Mushroom, Matriske (Russulaceae)	Norway	Norway	0.80	4

Category 23 Vegetables continued

Product	Manufacturer / product label / country of origin	Procured in	Antioxidant content in mmol/100g	Comment
Mushroom, Oyster		USA	0.06	6
Mushroom, Portabella		USA	0.22	6
Mushroom, Portabella, grilled		USA	0.34	6
Mushroom, Sheep polypore	Norway	Norway	3.85	4
Mushroom, Shiitake, stir-fried		USA	0.33	6
Mushroom, white		USA	0.38	6
Mushroom, white, microwave cooked		USA	0.43	6
Mushroom, white, stir-fried		USA	0.23	6
Okra (Abelmoschus esculentus)	Mali	Mali	0.42	3
Okra / gumbo, dry, fluor	Mali	Mali	4.24	3
Onion	Mali	Mali	0.33	3
Onion	Mali	Mali	0.22	3
Onion flakes	Natures Treats Australia PTY LTD, Australia	New Zealand	0.11	3
Onion, boiled		Norway	0.34	3
Onion, red	Italy	Norway	0.71	3
Onion, red, Red Baron	Norwegian University of Life Sciences	Norway	0.67	5
Onion, small	Mali	Mali	0.18	3
Onion, white	Italy	Norway	0.12	3
Onion, yellow		USA	0.24	6
Onion, yellow	Norway	Norway	0.64	3
Onion, yellow, cooked		USA	0.26	6
Parsnip	France	Norway	0.11	3
Parsnip	Netherlands	Norway	0.08	3
Pepper, bell-, green		Norway	1.56	3
Pepper, bell-, green	Mali	Mali	0.44	3
Pepper, bell-, orange		Norway	1.94	3
Pepper, bell-, red		Norway	1.81	3
Pepper, bell-, yellow		Norway	1.79	3
Pepper, green		USA	0.26	6

Category 23 Vegetables continued

Product	Manufacturer / product label / country of origin	Procured in	Antioxidant content in mmol/100g	Comment
Pepper, green, cooked		USA	1.22	6
Pepper, red		USA	0.91	6
Pepper, red, cooked		USA	1.64	6
Pickle, just the solution from cucumber pickles	Sweets Favorite	USA	0.15	3
Pickle, just the solution from cucumber pickles (whole)	Nora, Stabburet, Norway	Norway	0.07	3
Potato, blue, Congo	France	Norway	0.93	3
Potatoes	Mali	Mali	0.13	3
Potatoes, Beate	Norway	Norway	0.06	3
Potatoes, red		USA	0.22	6
Potatoes, red, cooked		USA	0.45	6
Potatoes, Roseval	France	Norway	0.08	3
Potatoes, Russet		USA	0.42	6
Potatoes, Russet, cooked		USA	0.50	6
Potatoes, white		USA	0.19	6
Potatoes, white, cooked		USA	0.43	6
Pumpkin		Norway	0.05	3
Pumpkins	Mali	Mali	0.02	3
Radishes		USA	0.12	6
Radishes	Primaflor, Spain	Norway	0.39	3
Radishes	France	Norway	0.40	3
Rhubarb	Norway	Norway	0.62	4
Savoy cabbage		Norway	0.42	3
Savoy cabbage, Taler	Norwegian University of Life Sciences	Norway	0.40	5
Spinach	Italy	Norway	0.89	3
Spinach, chopped, frozen	Store Brand	USA	0.97	6
Spinach, chopped, frozen	Birds Eye	USA	1.00	6
Spinach, chopped, frozen	Green Giant	USA	1.01	6
Spinach, chopped, frozen, boiled	Store Brand	USA	0.99	6
Spinach, chopped, frozen, boiled	Birds Eye	USA	1.13	6
Spinach, chopped, frozen, microvawe cooked	Store Brand	USA	1.10	6
Spinach, chopped, frozen, microvawe cooked	Birds Eye	USA	1.21	6
Spinach, frozen	Birds Eye	USA	1.31	6

Category 23 Vegetables continued

Product	Manufacturer / product label / country of origin	Procured in	Antioxidant content in mmol/100g	Comment
Spinach, frozen	Store Brand	USA	1.17	6
Spinach, frozen, boiled	Store Brand	USA	1.10	6
Spinach, frozen, boiled	Birds Eye	USA	1.11	6
Spinach, frozen, microwave cooked	Store Brand	USA	1.31	6
Spinach, frozen, microwave cooked	Birds Eye	USA	1.35	6
Sugar peas	Safar, Kenya	Norway	0.64	3
Summer squash, green	Spain	Norway	0.08	3
Summer squash, green	Norway	Norway	0.11	3
Summer squash, yellow	Spain	Norway	0.06	3
Swede (rutabaga)		Norway	0.43	3
Swede (rutabaga), Vige	Norwegian University of Life Sciences	Norway	0.37	5
Sweet onions		USA	0.15	6
Sweet potato, blue (Yam), peeled		Norway	0.54	3
Sweet potatoe	Mexico	Norway	0.24	3
Sweet potatoe		USA	0.08	6
Sweet potatoe, baked		USA	0.79	6
Sweet potatoe, boiled		USA	0.33	6
Sweet potatoe, pale	New Zealand	New Zealand	0.20	3
Sweet potatoe, red/white	Mali	Mali	0.16	3
Sweet potatoe, yellow	Mali	Mali	0.12	3
Syrup from red carrot	Roopaks, Ajmal Khan, N. Dehli	India	0.07	3
Tomato juice	Welch's	USA	0.19	3
Tomato juice	Cadiso, Denmark	Norway	0.41	3
Tomato juice	S&W Fine Food, USA	Norway	0.81	3
Tomato juice	Maroc	Norway	0.25	3
Tomato juice	Campbell's	USA	1.06	3
Tomato juice	Eckes-Granini	Norway	0.26	3
Tomato juice	Cofrutos	Norway	0.34	3
Tomato juice	Granini	Norway	0.23	3
Tomato juice	Campbell's	Norway	0.90	3
Tomato juice	Molinera	Norway	0.41	3
Tomato juice	Stop&Shop	USA	0.70	3

Category 23 Vegetables continued

Product	Manufacturer / product label / country of origin	Procured in	Antioxidant content in mmol/100g	Comment
Tomato juice, ecological	Svanes	Norway	0.44	3
Tomato juice, Premium	Del Monte	Norway	0.25	3
Tomato juice, Sun-C	Cadiso, Denmark	Norway	0.44	3
Tomatoes	Norway	Norway	0.22	3
Tomatoes		USA	0.16	6
Tomatoes	Mali	Mali	0.30	3
Tomatoes in tomato juice, canned, whole	Store Brand	USA	0.24	6
Tomatoes in tomato juice, canned, whole	Hunt's	USA	0.26	6
Tomatoes in tomato juice, canned, whole	Red Gold	USA	0.23	6
Tomatoes, canned, chopped, heated	ICA	Norway	0.49	3
Tomatoes, cherry-	The Greenery, Netherlands	Norway	0.62	3
Tomatoes, cherry-	Netherlands	Norway	0.34	3
Tomatoes, cherry-	Jone Wiig, Norway	Norway	0.39	3
Tomatoes, chopped	Del Monte	Norway	0.33	3
Tomatoes, chopped, canned	ICA	Norway	0.42	3
Tomatoes, chopped, canned	Biona	Norway	0.31	3
Tomatoes, chopped, canned	Euro Shopper	Norway	0.39	3
Tomatoes, chopped, canned, heated	Euro Shopper	Norway	0.48	3
Tomatoes, cluster tomatoes	Netherlands	Norway	0.29	3
Tomatoes, cluster tomatoes	Norway	Norway	0.17	3
Tomatoes, cluster tomatoes, small	Vereijken KwekerijenBV, Netherlands	Norway	0.39	3
Tomatoes, cluster tomatoes, small	Jone Wiig, Norway	Norway	0.33	3
Tomatoes, coarsely chopped, canned	Eldorado	Norway	0.25	3
Tomatoes, coarsely chopped, canned	Landlord	Norway	0.38	3
Tomatoes, cooked		USA	0.22	6
Tomatoes, crushed, passed, canned	Mutti	Norway	0.37	3
Tomatoes, organic peeled tomatoes, canned	Biona	Norway	0.28	3
Tomatoes, plum-	Netherlands	Norway	0.35	3

Category 23 Vegetables continued

Product	Manufacturer / product label / country of origin	Procured in	Antioxidant content in mmol/100g	Comment
Tomatoes, plum-	Spain	Norway	0.24	3
Tomatoes, steak-	Netherlands	Norway	0.18	3
Tomatoes, sundried	Norgesfrukt	Norway	1.30	3
Tomatoes, sundried, in oil	HGL Gourmet Line's middelhavskjøkken	Norway	1.95	3
Tomatoes, sundried, in oil	Santa Maria	Norway	1.36	3
Tomatoes, sundried, in oil	ICA	Norway	0.66	3
Tomatoes, whole, canned	SW	Norway	0.20	3
Tomatoes, whole, canned	Eldorado	Norway	0.28	3
Tomatoes, whole, canned	Euro Shopper	Norway	0.41	3
Tomatoes, whole, canned	Landlord	Norway	0.31	3
Tomatoes, with chili, extra rich, crushed, without skin, canned	ICA	Norway	0.56	3
Tomatoes, with garlic, extra rich, crushed, without skin, canned	ICA	Norway	0.61	3
Tomatoes, with roasted garlic, canned	SW	Norway	0.22	3
Tomatoes, with sweet basil, crushed, without skin, canned	ICA	Norway	0.77	3
Tomatos, sundried, in canola oil	Sandhurst Fine Foods, Australia	New Zealand	3.59	3
Turnip	Mali	Mali	0.29	3
Vegetable juice	V8	Norway	0.50	3
Vegetable juice	Eckes-Granini	Norway	0.27	3
Yam	Mali	Mali	0.22	3

Category 24 Vitamin and dietary supplements

Product	Manufacturer / product label / country of origin	Procured in	Antioxidant content in mmol/100g	Comment
Almond oil	Apotekproduksjon, Norway	Norway	1.13	1, 3
Aloe Vera Gel		Norway	0.13	3
Alpha Lipoic Acid, 50mg	Nature Made	USA	2.28	3
Amway Nutrilite Double X, Bronze	Quixtar	USA	30.81	3
Amway Nutrilite Double X, Gold	Quixtar	USA	35.68	3
Amway Nutrilite Double X, Silver	Quixtar	USA	29.72	3
Antioxidant capsules, Medox	MedPalett Pharmaceuticals AS	Norway	444.20	5
Antocyanin ascorbates, Aronia	Polyphenols Laboratories AS	Norway	725.35	5
Antocyanin capsules, Cherry	Polyphenols Laboratories AS	Norway	329.54	5
Apricot kernel oil	Aqua Oleum	Norway	0.98	1, 3
Balance, diluted	Pharmanex, Netherlands	Norway	0.64	5
Balance, powder	Pharmanex, Netherlands	Norway	7.13	5
Bausch & Lomb Ocuvite	Bausch & Lomb, USA	USA	293.78	3
Bayer One A Day Essential	Bayer HealthCare, USA	USA	222.32	3
B-carotene, capsules	GNC	USA	2.03	1, 3
Body Wise Right Choice AM	Body Wise International	USA	530.63	3
Body Wise Right Choice PM	Body Wise International	USA	0.88	3
Brewer's Yeast, 7.5 grain tablet	Puritan's Pride	USA	1.74	3
Bronson Garlic Oil, softgel 1 mg	Bronson	USA	0.95	2, 3
Catechin 100, Green-tea capsules		Norway	536.05	1, 3
Centrum	Wyeth Consumer Healthcare	USA	44.80	3

Category 24 Vitamin and dietary supplements continued

Product	Manufacturer / product label / country of origin	Procured in	Antioxidant content in mmol/100g	Comment
Centrum Silver	Wyeth Consumer Healthcare	USA	52.51	3
Centrum Silver	Whitehall-robins Healthcare, USA	USA	40.51	3
Centrum with lutein	Whitehall-robins Healthcare, USA	USA	43.56	3
Chinese chili oil		Japan	2.29	2, 3
Chondroitin Sulfate, 400 mg	GNC	USA	0.05	3
Citrosept, extract from grapefruit kernels	Citamani Europe AS	Norway	12.58	3
Coconut oil	KTC(Edibles)Ltd, England	Norway	1.07	1, 3
Cod liver oil	Cumberland Swan	USA	0.19	2, 3
Cod liver oil	Peter Møller, Norway	Norway	0.34	3
Coenzyme Q10, 10 mg	Bronson	USA	0.08	3
Complete One	Puritan's Pride	USA	165.90	3
Coneflower solution		Norway	0.42	3
CVS Daily Vitamin (no minerals)	CVS	USA	249.56	3
CVS Iron Slow release, 50 mg	CVS	USA	0.77	3
CVS Multivitamin with minerals	CVS	USA	12.35	3
CVS plus iron	CVS	USA	260.97	3
CVS Vitamin B 12, 250 mcg	CVS	USA	0.19	3
CVS Vitamin C (as ascorbic acid), 500 mg	CVS	USA	1019.69	3
CVS Vitamin C with Rose Hips, 500 mg	CVS	USA	796.59	3
CVS Vitamin E (d alpha tocopherol) 400 IU	CVS	USA	320.70	2, 3
Dog Rose oil	Nature Drogeriet A/S	Norway	0.55	3
Drink Mix vanilla flavored, diluted	Carb safe	USA	0.04	3
Drink Mix vanilla flavored, powder	Carb safe	USA	0.67	3
Echinacea cocktail	Bræmhults, Sweden	Norway	0.66	3
Folat pills		Norway	0.05	3
Forward Multi-Nutrient Oacket	Healthy Directions Corp	USA	138.54	3
Gamma E capsules, oil	Sunkost	Norway	39.97	1, 3

Category 24 Vitamin and dietary supplements continued

Product	Manufacturer / product label / country of origin	Procured in	Antioxidant content in mmol/100g	Comment
Geritol Liquid	SmithKline Beecham	USA	0.33	3
Ginkgo Biloba, 60 mg	Bronson	USA	35.85	3
Ginseng cocktail	Bræmhults, Sweden	Norway	1.20	3
Ginseng, Panax, solution	Ortis	Norway	0.20	3
Glucosamine sulfate, 500 mg	Walgreen	USA	0.67	3
GNC brewer's yeast powder, 100 g	GNC	USA	2.45	3
GNC Spirulina, 500 mg capsules	GNC	USA	5.97	3
GNC Ultra Mega Gold	GNC	USA	235.55	3
Grape Seed Extract, 50 mg	Bronson	USA	108.13	3
Jungamals Life Pak for Kids	Pharmanex, Netherlands	Norway	36.27	5
Kirkland high energy pak	Leiner Health Products	USA	0.01	3
Kirkland high energy pak (Chromium Picolinate)	Leiner Health Products	USA	0.02	3
Kirkland high energy pak (Ginseng Concentrate)	Leiner Health Products	USA	1.78	2, 3
Kirkland high energy pak (Multivitamin/mineral)	Leiner Health Products	USA	0.69	3
Kirkland high energy pak (Vitamin C)	Leiner Health Products	USA	1052.44	3
Kirkland high energy pak (Vitamin E)	Leiner Health Products	USA	6.05	2, 3
Korean Ginseng, extract	Bando Korea	Norway	1.48	3
Lecithin, 1200 mg	Nature's Bounty	USA	3.71	2, 3
Lederle Protegra		USA	288.68	3
Lederle Stresstabs		USA	613.49	3
Life Extension High Potency Antioxidant	Life Extension Foundation	USA	281.20	3
Life Pak Essentials	Pharmanex, Netherlands	Norway	49.62	5
LifePak	Pharmanex, USA	Norway	62.16	5
Marine Omega	Pharmanex, Netherlands	Norway	4.26	2, 5
Metamucil, dierary fiber suplement, orange, powder		USA	0.57	3
Metamucil, dierary fiber suplement, powder	Procter& Gamble	USA	0.34	3
Multiminerals, supplements	Nycomed Pharma	Norway	42.83	3

Category 24 Vitamin and dietary supplements continued

Product	Manufacturer / product label / country of origin	Procured in	Antioxidant content in mmol/100g	Comment
Myadec	Pfizer Consumer Healthcare	USA	51.38	3
Natrol DHEA, 25 mg	Natrol	USA	0.15	3
Natto extract, capsules	Nattkin Co, Japan	Norway	2.29	1, 3
Natto extract, capsules		Norway	3.11	1, 3
Nature Made Balanced B-50	Nature Made	USA	0.07	3
Nature Made cod liver oil, capsules	Nature Made	USA	0.62	2, 3
Nature Made folic acid, 400 mcg	Nature Made	USA	0.12	3
Nature Made Magnesium, 250 mg	Nature Made	USA	0.01	3
Nature Made Potassium Gluconate, 90 mg	Nature Made	USA	0.04	3
Nature made Vitamin B6, 100 mg	Nature Made	USA	0.08	3
Nature's Bounty Beta Carotene Pro Vitamin A, 25000 IU	Nature's Bounty	USA	2.56	2, 3
Nature's Bounty Calcium Citate, 200 mg	Nature's Bounty	USA	0.07	3
Nature's Bounty Chromium Picolinate, 500 mcg	Nature's Bounty	USA	0.14	3
Nature's Bounty Ginseng Royal Jelly plus	Nature's Bounty	USA	2.13	3
Nature's Bounty Melatonin, 3 mg	Nature's Bounty	USA	0.69	3
Nature's Bounty Niacin, 250 mg	Nature's Bounty	USA	0.15	3
Nature's Bounty Salmon oil, 1000 mg softgels	Nature's Bounty	USA	0.32	2, 3
Nature's Bounty Vitamin A, 10000IU	Nature's Bounty	USA	3.47	2, 3
Nature's Bounty Vitamin D 400 IU	Nature's Bounty	USA	0.12	3
Nature's Resource St.John's Wort	Nature's Resource	USA	118.54	3
Noni, capsules	Fitness Pharma	USA	14.76	3
Ocuvite ekstra, pill	Bausch & Lomb, USA	USA	281.14	3
One A Day 50 Plus	Bayer HealthCare, USA	USA	23.25	3

Category 24 Vitamin and dietary supplements continued

Product	Manufacturer / product label / country of origin	Procured in	Antioxidant content in mmol/100g	Comment
One A Day Maximum	Bayer HealthCare, USA	USA	4.48	3
One a day Men`s Health Formula, pill	Bayer HealthCare, USA	Norway	56.69	3
One A Day Women's Formula	Bayer HealthCare, USA	USA	32.48	3
Over drive	Pharmanex, Netherlands	Norway	301.83	5
Purple Coneflower, Echinagard	Madaus AG, Germany	Norway	0.11	3
Pycogenol	Bronson	USA	49.58	3
Rexall Lycopene, 10 mg	Rexall	USA	3.27	2, 3
Safflower oil	Naturata Spielberger, Germany	Norway	1.38	1, 3
Sambucol-C	Medtech Pharma	USA	65.81	3
Saw Palmetto, 20 mg	Bronson	USA	0.49	3
Schiff Vegetarian Multiple	Schiff	USA	185.74	3
Selenium, 100 mcg	CVS	USA	0.95	3
Shaklee Vita-Lea, with Iron	Shaklee Corp	USA	19.36	3
Shaw's Calcium Hi Cal (oyster shell), 500 mg	Shaw's	USA	0.01	3
Solotron (includes iron)	GNC	USA	140.08	3
Super Antioxidant	Pharmanex, Netherlands	Norway	449.98	5
Tahitian Noni juice		Norway	0.94	3
Tegreen	Pharmanex, USA	Norway	731.18	5
Theragran M	Bristol Myers Squibb	USA	197.60	3
Theragran, pill	Manufactured in China for Bristol-Meyers products, USA	Norway	29.97	3
Triente plus	Pathway	USA	77.48	3
Tums (calcium 200 mg)		USA	0.04	3
VIACTIV Chocolate (calcium 500 mg)	Viactiv	USA	1.99	3
Vita amino nopal (capsules)	Mexico	Mexico	4.70	3
Vitamine A capsules, fish liver oil, 10000IU		Norway	2.53	1, 3
Vitamine E, (dl alpha-tocopherol), 400 IU	CVS	USA	2.95	2, 3

Category 24 Vitamin and dietary supplements continued

Product	Manufacturer / product label / country of origin	Procured in	Antioxidant content in mmol/100g	Comment
Walgreen Super Aytinal Active Adults	Walgreen	USA	87.70	3
Walnut oil	Leon Frenkel Ltd, England	Norway	1.40	1, 3
Wheat germ oil	Apotekproduksjon, Oslo	Norway	3.04	1, 3
Women's Ultra mega	GNC	USA	11.29	3
Z-BEC	Inverness Medical	USA	701.93	3
Zinc, 50 mg	CVS	USA	0.04	3
AARP Formula 196, no iron		USA	3.88	3

Comments:

1 Analyzed / extracted in water/2-propanol (1+9, v/v).

2 Analyzed /extracted in 2-propanol.

3 Purchased in grocery store, restaurant, cafe, bakery or marketplace.

4 Handpicked or received directly from supplier.

5 Previously published in Halvorsen et al. 2002 or Dragland et al. 2003

6 Previously published in Halvorsen et al. 2006.
